# Checklist of the ichthyofauna of the Rio Negro basin in the Brazilian Amazon

**DOI:** 10.3897/zookeys.881.32055

**Published:** 2019-10-17

**Authors:** Hélio Beltrão, Jansen Zuanon, Efrem Ferreira

**Affiliations:** 1 Universidade Federal do Amazonas – UFAM; Pós-Graduação em Ciências Pesqueiras nos Trópicos PPG-CIPET; Av. Rodrigo Otávio Jordão Ramos, 6200, Coroado I, Manaus-AM, Brazil Universidade Federal do Amazonas Manaus Brazil; 2 Instituto Nacional de Pesquisas da Amazônia – INPA; Coordenação de Biodiversidade; Av. André Araújo, 2936, Caixa Postal 478, CEP 69067-375, Manaus, Amazonas, Brazil Instituto Nacional de Pesquisas da Amazônia Manaus Brazil

**Keywords:** Blackwater, conservation, diversity, freshwater fish, ichthyofaunal survey

## Abstract

This study presents an extensive review of published and unpublished occurrence records of fish species in the Rio Negro drainage system within the Brazilian territory. The data was gathered from two main sources: 1) litterature compilations of species occurrence records, including original descriptions and revisionary studies; and 2) specimens verification at the INPA fish collection. The results reveal a rich and diversified ichthyofauna, with 1,165 species distributed in 17 orders (+ two incertae sedis), 56 families, and 389 genera. A large portion of the fish fauna (54.3% of the species) is composed of small-sized fishes < 10 cm in standard length. The main groups are Characiformes (454 species; 39.0%), Siluriformes (416; 35.7%), Gymnotiformes (105; 9.0%), and Cichliformes (102; 8.8%). The species composition differs between the main aquatic environments, such as: main channel (159 species), lakes (296), tributary rivers (596), small streams (234), seasonal beaches (186), and rapids (41). Part of the ichthyofauna is shared with adjacent basins, such as the Orinoco, rivers of the Guiana Shield, lower Solimões/Amazonas and upper Amazonas, which contributes to the remarkable ichthyofaunal diversity of the basin. A high rate of species endemism was observed in Characidae (24), Loricariidae (18), Cichlidae (18) and Callichthyidae (18), totalling 156 species (13.4%) endemic to the basin. An estimation of the species richness for the Rio Negro basin, considering 23 published references, resulted in 1,466 and 1,759 species (Jackknife 1 and 2, respectively), which seems reasonable when considering the large number of morphotypes left out of the present list and the low sampling effort in many areas of the basin. The results presented herein provide an additional tool for environmental managers and decision makers for conservation purposes of one of the richest and most well-preserved sub-basins of the Rio Amazonas system.

## Introduction

The Amazon basin is the largest hydrographic system in the world, with a total of 6,869,000 km^2^ (Goulding et al. 2003), and harbors the richest freshwater ichthyofauna of the planet, predominantly composed of species of the Superorder Ostariophysi (85%) (Roberts 1972; Lowe-McConnell 1987; Reis et al. 2003, 2016). This richness comprises numerous evolutionary lineages, resulting from the interaction of multi-layered geological patterns associated with vicariant and dispersal agents (Lundberg et al. 1998; Dagosta and de Pinna 2017). The distribution of the fish species in the Amazon sub-basins and adjacent drainage systems is complex, and amounts to numerous distributional overlaps and superlative degrees of biogeographic congruence (Dagosta and de Pinna 2017). Currently, more than 2,700 fish species have been formally described for the Amazon basin (Reis et al. 2016; Dagosta and de Pinna 2019), but recent estimates suggest that this number exceeds 3,000 species (Carvalho et al. 2007). However, this number possibly will increase significantly with the addition of ichthyofaunal inventories in poorly sampled areas; with the accumulation of taxonomic reviews; and with the use of novel information derived from molecular and other analytical tools. The true species richness in the Amazon basin may rise to approximately 4,000–5,000 species in the next 40 to 70 years, if the description rate remains at the current level (see Ota et al. (2015) for an estimative for Siluriformes; and Birindelli and Sidlauskas (2018) for freshwater fishes in Neotropical region).

The Rio Negro is one of the main tributaries of the Amazon basin, with a drainage area of approximately 696,808 km^2^ that is mostly (80%) contained in Brazilian territory (Goulding et al. 2003). Its extension from the headwaters located in Colombia and Venezuela and its mouth on the Rio Amazonas/Solimões is of approximately 2,250 km (Goulding et al. 2003). The Rio Negro has an average discharge of 28,000 m^3^ per second, corresponding to ca. 15% of the discharge of the Rio Amazonas at its mouth, being the fifth largest river in volume of water of the world (Goulding et al. 2003). The water level of the Rio Negro follows the rainfall regime of the region and shows a great variation throughout the year, with a difference of 10 - 12 m between the peak of flood period and the lowest level at the end of the dry period (as measured in Manaus Municipality; Irion et al. 1997). This sub-basin is characterized by geologically ancient (Paleozoic) and quite leached sediments, in which sandy, acidic and nutrient-poor soils prevail (Sioli 1950; Fittkau et al. 1975; Küchlera et al. 2000). Moreover, the Rio Negro waters are characterized by the presence of large amounts of dissolved organic carbon in the form of humic and fulvic acids, contributing to the acidity of the water and to its reddish color (that looks black in deep waters), despite its high transparency (Fittkau et al. 1975; Junk and Furch 1985; Goulding et al. 1988). Consequently, the Rio Negro presents highly acidic waters (pH < 5.0), with low suspended sediment load (corresponding to approximately only 2–3% of the amount of suspended solids transported by Rio Solimões, for instance Fisher 1978), remarkably poor in nutrients (especially cations) and low values of electrical conductivity (as low as 8–13 μS/cm), which may represent extreme ecological conditions for many species of animals and plants (Fittkau et al. 1975; Küchlera et al. 2000).

Those supposedly harsh limnological conditions, however, do not constitute a limiting factor for the diversity of fishes, and the Rio Negro waters harbor one of the most diversified ichthyofaunas in the world. So far, Goulding et al. (1988) carried out the most complete inventory of the ichthyofauna of that basin, recording approximately 450 fish species, but pointing out the possible presence of up to 700 species. At the time of that publication the estimated fish richness for the Rio Negro was superior to the encountered in all the European rivers (233 species), in the Mississippi/Missouri River in the USA (375 species) (Lévêque et al. 2008), or in all the Argentinian territory (339 species) (López et al. 2002), and being comparable to that of the entire Congo/Zaire River basin in Equatorial Africa (Skelton 2001).

Despite this outstanding richness, the fish fauna inhabiting blackwater rivers is poorly studied when compared to that of white-water rivers in the Amazon (Kramer et al. 1978; Goulding 1980; Bayley 1998; Junk et al. 1997; Barthem and Goulding 1997; Saint Paul et al. 2000; Junk et al. 2007; Zuanon et al. 2007; Queiroz et al. 2013; Ohara et al. 2015). One of the first surveys of the Rio Negro ichthyofauna was performed by the British naturalist Alfred Russel Wallace between 1850 and 1852. However, the preserved specimens were destroyed by a fire and the consequent shipwreck of the ship carrying the sampled material when on the way to Europe, leaving only the illustrations of ca. 180 fish species that were donated to the Museum of Natural History of London; such pictorial records were later found in the museum archives and published in a book by Toledo-Piza Ragazzo (2002). In addition to the seminal work of Goulding et al. (1988), more recent studies have provided complementary information regarding the Rio Negro fish fauna, which include ecological studies of the main river channel (Chao et al. 2001; Thomé-Souza and Chao 2004; Ferreira et al. 2007; Rapp Py-Daniel et al. 2017), lakes and flooded forest areas (igapós) (Garcia 1993; Saint Paul et al. 2000; Soares and Yamamoto 2005; Ferreira et al. 2007; Noveras et al. 2012; Yamamoto et al. 2014; Farias et al. 2017; Beltrão and Soares 2018), seasonal beaches and rapids (Lima et al. 2005; Ferreira et al. 2007), streams and small marginal ponds (Knöppel 1970; Silva 1993, 1995; Mendonça et al. 2005; Pazin et al. 2006; Anjos and Zuanon 2007; Zuanon et al. 2015; Beltrão and Soares 2018), interfluvial swamps (Chao and Prada-Pedreros 1995), flooded Savannah-like areas (Ferreira et al. 2007), as well as ichthyofaunal inventories of specific tributaries (Henderson and Walker 1986; Chao and Prada-Pedreros 1995; Zuanon et al. 1998; Lima et al. 2005; Ferreira et al. 2007; Zuanon et al. 2008; Kemenes and Forsberg 2014; Rapp Py-Daniel et al. 2017). This study aimed to make a comprehensive survey of the fish species present in the Rio Negro basin, as well as to analyze the fish diversity associated to the different aquatic environments present in the basin (Goulding et al. 1988). The compilation of the available information (both published and in databanks) presented herein provides a general picture of the distribution of fish species in the Rio Negro basin by habitat types; allows an appreciation of the species description rate in the basin along time; and provides estimates of the total fish species richness for the basin.

## Materials and methods

Fish species occurrence records for the Rio Negro basin were obtained from two main sources of information. First, a compilation of the records of species originally described based on specimens collected in the Rio Negro basin or that included that basin in their distribution range was made based in the catalogues of Reis et al. (2003), Buckup et al. (2007), Ferraris-Jr (2007), and Ferraris-Jr et al. (2017). Additional information from taxonomic revisions and species descriptions from 2003 to 2019 were also included: Armbruster (2003), Britto and Lima (2003), Costa (2003a, 2003b), Vari and Lima (2003), Costa (2004a, 2004b), Crampton et al. (2004), Hrbek et al. (2004), Lehmann and Reis (2004), Malabarba (2004), Zanata and Toledo-Piza (2004), Zarske et al. (2004), Ferraris-Jr et al. (2005), Kullander and Ferreira (2005), Lundberg and Akama (2005), Reis et al. (2005), Sabaj (2005), Sousa and Rapp Py-Daniel (2005), Vari et al. (2005), Zanata and Lima (2005), Armbruster and Page (2006), Bührnheim and Malabarba (2006), Ferreira and Lima (2006), Kullander and Ferreira (2006), Mautari and Menezes (2006), Mattox et al. (2006), Menezes (2006), de Santana and Nogueira (2006), Zarske and Géry (2006), Parisi et al. (2006), Lundberg and Cox-Fernandes (2007), Bührnheim and Malabarba (2007), Buitrago–Suárez and Burr (2007), Ferraris-Jr (2007), Lucena (2007), Rodriguez and Reis (2007), Staeck and Schindler (2007), Zarske and Géry (2007), Armbruster (2008), Birindelli et al. (2008), Orti et al. (2008), Sabaj and Birindelli (2008), Sabaj et al. (2008), Sidlauskas and Vari (2008), Rapp Py-Daniel and Fichberg (2008), Rocha et al. (2008a, 2008b), Thomas and Rapp Py-Daniel (2008), Birindelli and Britski (2009), de Santana and Vari (2009), Lima and Sousa (2009), Lima et al. (2009), Marinho (2009), Marinho and Lima (2009), Oyakawa and Mattox (2009), Paixão and Toledo-Piza (2009), Sullivan (2009), Vari and Ferraris-Jr (2009), Bertaco and Carvalho (2010), Carvalho et al. (2010), de Santana and Vari (2010), Lucena and Malabarba (2010), Marinho (2010), Marinho and Langeani (2010), Peixoto and Wosiacki (2010), Ribeiro and Rapp Py-Daniel (2010), Römer et al. (2010), Arbour and López-Fernández (2011), Birindelli et al. (2011), Schindler and Valdesalici (2011), Kullander (2011), Marshall et al. (2011), Maxime et al. (2011), Melo et al. (2011), Netto-Ferreira et al. (2011), de Pinna and Kirovsky (2011), de Santana and Crampton (2011), Sousa and Birindelli (2011), Birindelli and Zuanon (2012), Birindelli et al. (2012), Loeb (2012), Rocha et al. (2012), Andrade et al. (2013), Costa et al. (2013), Costa and Bragança (2013), Marinho et al. (2013), Mattox et al. (2013), Peixoto et al. (2013), Roberts (2013), Birindelli (2014), Britzki et al. (2014), Burns et al. (2014), Calegari et al. (2014), Coutinho and Wosiacki (2014), Cox-Fernandes et al. (2014), Ivanyisky and Albert (2014), Melo and Vari (2014), Menezes and Lucena (2014), Ota et al. (2014), Toledo-Piza et al. (2014), Vari and Calegari (2014), Zarske (2014), Cox-Fernandes et al. (2015), de Oliveira et al. (2015), Kullander and Varella (2015), Marinho et al. (2015), Ottoni (2015), Silva-Oliveira et al. (2015), Hernández-Acevedo et al. (2015), Peixoto et al. (2015), Walsh et al. (2015), Carvalho et al. (2016), Henschel (2016), Ota et al. (2016), Ray and Armbruster (2016), Tan and Armbruster (2016), Tan et al. (2016), Tencatt and Britto (2016), Tencatt and Ohara (2016), Britski and Birindelli (2016), Bernt and Albert (2017), Burns et al. (2017), Evans et al. (2017), Ferraris-Jr et al. (2017), Fontenelle and Carvalho (2017), Sabaj and Hernández (2017), Ribeiro et al. (2017), Marinho and Menezes (2017), Melo and Oliveira (2017), de Pinna et al. (2017), Bernt et al. (2018), Bragança (2018), Caires and Toledo-Piza (2018), Henschel et al. (2018), Mateussi et al. (2018), Silva and Rapp Py-Daniel (2018), Urbanski et al. (2018), and Reia and Benine (2019). Information was also collected from publications focusing on biogeographic analyses and large taxonomic fish inventories in specific areas or sub-basins of the Rio Negro drainage system: Knöppel (1970), Henderson and Walker (1986), Goulding et al. (1988), Barletta (1995), Chao and Prada-Pedreros (1995), Silva (1995), Zuanon et al. (1998), Saint-Paul et al. (2000), Chao (2001), Mortati (2004), Thomé-Souza and Chao (2004), Soares and Yamamoto (2005), Lima et al. (2005), Anjos and Zuanon (2007), Ferreira et al. (2007), Galuch (2007), Zuanon et al. (2008), Noveras et al. (2012), Kemenes and Forsberg (2014), Zuanon et al. (2015), Farias et al. (2017), Rapp Py-Daniel et al. (2017), Beltrão and Soares (2018), and Beltrão et al. (2018).

The second main source of information was composed by a thorough survey for all species that were collected in the Rio Negro basin and that have lots deposited in the INPA Fish Collection, which were personally verified by the authors. Morphotypes of nearly 40 new, undescribed species that were considered unequivocally distinct from the known species in their respective genera (mostly based on recently reviewed genera) were also included in the list. An update of the taxonomic nomenclature and geographic distribution of the species (at river-basin scale) was made using the published studies of Reis et al. (2003), Ferraris-Jr (2007), Ferraris-Jr et al. (2017), Dagosta and de Pinna (2019), and the FishBase online catalog (www.fishbase.org).

Exotic species that were introduced purposely or accidentally by fish farming or aquarium releases in impacted urban streams in Manaus, which have vouchers in the fish collections of INPA and the Federal University of Amazonas (UFAM), were also included in the list.

To provide an overview of the amount of described species originating from the Rio Negro basin, a species accumulation curve was constructed based on published information since the year 1821. Additionally, based on the 23 aforementioned ichthyofaunistic published studies, an estimative of total species richness in the Rio Negro basin was done using Jackknife 1 and 2 (Magurran 1988) using Past 3.0 statistical package (Hammer et al. 2001).

**Figure 1.**
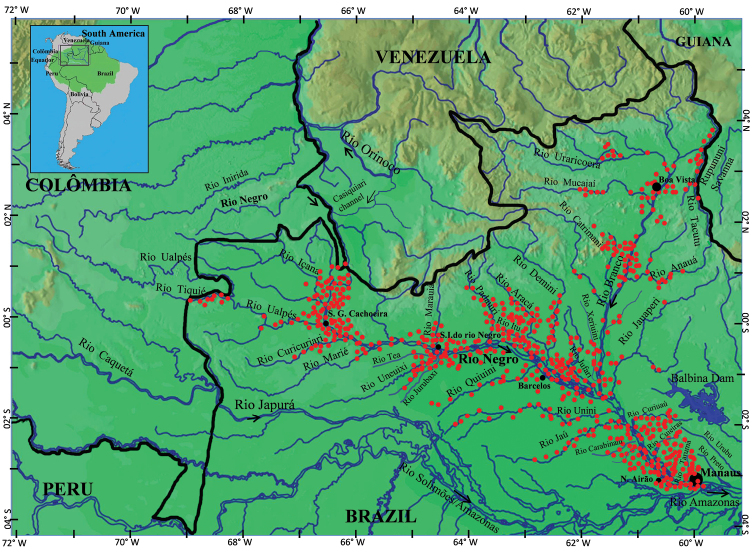
Map of the Rio Negro basin in the northwestern portion of Brazil and adjacent countries, depicting its main tributaries and sampling localities (red dots) obtained from the descriptions of new species 2003–2019, fish collection records, and published ichthyofaunal inventories.

The species list presented herein follows the taxonomic classification of Betancur et al. (2017), with the orders arranged following the systematic/phylogenetic organization of the latter, whereas families, genera and species are presented in alphabetical order. Regarding the distribution of each species, the information regarding habitat use/preference was obtained from the literature and/or from labels of preserved voucher specimens in fish collections. Finally, a map of the Rio Negro basin with the sampling localities mentioned in the bibliographic and collection sources mentioned above is presented (Figure 1).

## Results

### Ichthyofauna of the Rio Negro basin

Overall, the present compilation includes the records of 1,165 fish species for the Rio Negro basin, distributed in 17 orders (+ 2 groups considered incertae sedis), 56 families and 389 genera (Table 1, Suppl. material 1). The ichthyofauna was predominantly represented by members of the Ostariophysi (83.7% of the species) with the most diverse orders being the Characiformes (454 species; 39.0%), Siluriformes (416; 35.7%), Gymnotiformes (105; 9.0%) and Cichliformes (102; 8.8%). The other 13 orders (+ 2 groups incertae sedis) were represented by 88 species (7.5%) (Suppl. material 1). The most species-rich families were the Characidae (195 species), Loricariidae (125), Cichlidae (102), Doradidae (54), Auchenipteridae (48), Pimelodidae (44), Serrasalmidae (44), Apteronotidae (41) and Curimatidae (38). Together, these families account for 59.3% of the total richness found herein (Suppl. material 1).

**Table 1.** Number of families, genera and species for each order of fishes recorded in the Rio Negro basin.

Order	Families	Genera	Species
Myliobatiformes	1	3	9
Osteoglossiformes	2	2	3
Clupeiformes	2	7	13
Cypriniformes (not native)	1	1	1
Characiformes	20	127	454
Siluriformes	12	165	416
Gymnotiformes	5	27	105
Batrachoidiformes	1	1	1
Gobiiformes	1	2	4
Anabantiformes (not native)	1	1	1
Synbrachiformes	1	1	3
Pleuronectiformes	1	4	5
Beloniformes	1	3	6
Cyprinodontiformes	2	7	26
Cichliformes	1	30	102
incertae sedis: Ovalentaria (Polycentridae)	1	2	2
Tetraodontiformes	1	1	1
incertae sedis: Eupercaria (Sciaenidae)	1	4	12
Lepidosireniformes	1	1	1
**17 (+2 incertae sedis)**	**56**	**389**	**1165**

**Figure 2.**
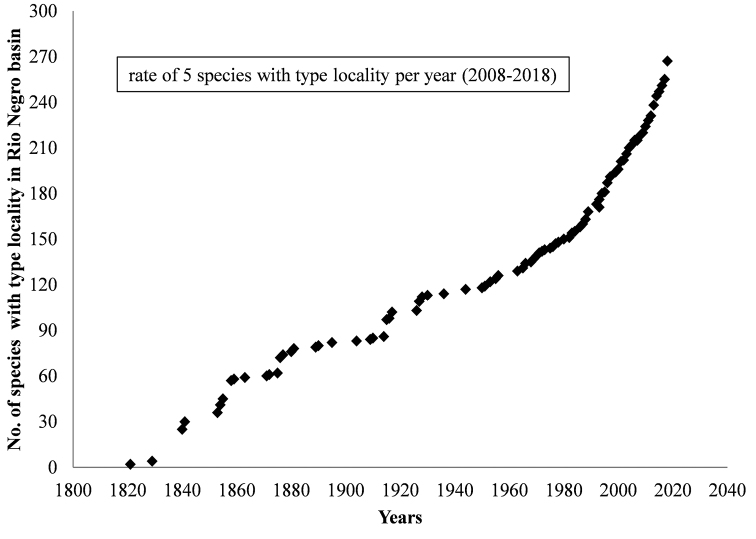
Cumulative curve of valid fish species numbers described from type localities in the Rio Negro basin between 1821 and 2019, based on the catalogs of Reis et al. (2003) and Buckup et al. (2007), and species descriptions published after those compilations.

So far, 271 valid species were originally described from the Rio Negro. The species accumulation curve (Figure 2) shows a high increase in the rate of species descriptions in the last three decades (1988 to the present date), with 112 species described (compared to just 36 species described between 1958 and 1987). The sharp increase in the species descriptions rate with time lends support to the provided estimate of total fish richness for the Rio Negro basin lying between 1,466 and 1,759 species (Jackknife 1 and 2, respectively).

The composition of fish species in the Rio Negro ranges from very large species to miniature forms (sensu Weitzman and Vari 1988). Among the largest fishes are the giant or goliath catfishes *Brachyplatystoma filamentosum* and *B. capapretum* (Siluriformes: Pimelodidae), which can reach approximately 2.8 m TL and more than 140 kg, the pirarucu *Arapaima gigas* (Osteoglossiformes: Arapaimidae) with approximately 3.0 m ST and 200 kg. On the other hand, miniature species include those recently described *Leptophilypnion fittkaui* (Gobiiformes: Eleotridae) with the maximum length of 9.2 mm SL, *Priocharax nanus* (13.0 mm SL) and *Cyanogaster noctivaga* (20.0 mm SL) (Characiformes: Characidae) and *Scoloplax baskini* (Siluriformes: Scoloplacidae) with 14.4 mm SL. In fact, a large portion of the Rio Negro fauna (634 species, 54.4%) have small or very small maximum sizes (< 10 cm of SL). Most these species are Characiformes of the families Characidae, Crenuchidae, Lebiasinidae, Iguandectidae and Curimatidae (265 species), catfishes (Loricariidae, Callichthyidae, Heptapteridae, Trichomycteridae, Doradidae, Auchenipteridae, Aspredinidae, and Cetopsidae, 224 species), dwarf cichlids Cichliformes (41 species), and Cyprinodontiformes (Poeciliidae and Rivulidae, 26 species) (Suppl. material 1).

Two genera of small Characidae (*Cyanogaster* and *Tucanoichthys*), two genera of Hypopomidae (*Procerusternarchus* and *Racenisia*), one genus each of Tarumaniidae (*Tarumania*), Trichomycteridae (*Glanapteryx*), and Loricariidae (*Niobichthys*) are only found in the Rio Negro basin. Small-sized endemic species belonging to genera with wide distribution in the Amazon and Orinoco basins were also recorded, e.g., *Astyanax ajuricaba*, *Creagrutus tuyuka*, *Jupiaba poekotero*, *Knodus tiquiensis*, *Hyphessobrycon paepkei*, *Priocharax nanus*, *Tyttobrycon xeruini* (Characidae), *Microsternarchus brevis* (Hypopomidae), *Hypopygus cryptogenes* (Rhamphichthyidae), *Brachyglanis nocturnus* (Heptapteridae), *Scoloplax baileyi*, *S. dolicholophia* (Scoloplacidae), and *Polycentrus jundia* (Polycentridae) (Suppl. material 1).

This compilation shows that 156 species (13.4%) are probably endemic to the Rio Negro drainage, for example, *Potamotrygon wallacei* (Potamotrygonidae), *Osteoglossum ferreirai* (Osteoglossiformes), *Brittanichthys myersi*, *Tucanoichthys tucano* (Characidae), *Corydoras adolfoi*, *C. tukano*, *C. burgessi*, *C. crimmeni*, *C. imitador* (Callichthyidae), *Physopyxis cristata* (Doradidae), *Brachyglanis nocturnus*, *Brachyrhamdia rambarrani* (Heptapteridae), *Laimosemion kirovskyi*, *L. amanapira*, and *L. uakti* (Rivulidae) (Suppl. material 1).

Among the species found in the Rio Negro, 436 (37.4%) have a wide distribution in the Amazon basin and in the adjacent watersheds, such as the Rio Orinoco basin, rivers of the Guianas shield (including the Essequibo, rivers of the Suriname and French Guiana, and the headwaters of rivers draining to the Rio Amazonas basin), and in Rio Tocantins basin, and inhabit a wide range of environments (cf. Dagosta and de Pinna 2019). This group is represented mainly by medium to large-sized species of the genera *Pellona* (Pristigasteridae), *Leporinus* (Anostomidae), *Acestrorhynchus* (Acestrorhynchidae), *Boulengerella* (Ctenoluciidae), *Curimata*, *Curimatella* (Curimatidae), *Cynodon*, *Hydrolycus* (Cynodontidae), *Hemiodus* (Hemiodontidae), *Serrasalmus* (Serrasalmidae), *Brachyplatystoma*, *Hypophthalmus*, *Pimelodus*, *Pseudoplatystoma* (Pimelodidae), *Apteronotus*, *Sternarchogiton*, *Sternarchorhamphus* (Apteronotidae), *Gymnotus* (Gymnotidae), *Brachyhypopomus* (Hypopomidae), and *Eigenmannia* (Sternopygidae) (Suppl. material 1). Some of these species carry out long-distance migrations (mainly in the reproductive period) that may reach thousands of kilometers, like the giant catfishes of the genus *Brachyplatystoma* (Pimelodidae).

At least 118 species (10.1%) are exclusively shared by the Negro and Orinoco basins, such as *Potamotrygon schroederi* (Potamotrygonidae), *Acestrorhynchus grandoculis* and *Heterocharax leptogrammus* (Acestrorhynchidae), *Pseudanos varii* (Anostomidae), *Creagrutus ephippiatus*, *C. phasma*, *C. runa*, *Hemigrammus bleheri*, *Hyphessobrycon diancistrus*, *H. epicharis*, *Paracheirodon axelrodi*, *P. simulans* (Characidae), *Elachocharax geryi* and *E. mitopterus* (Crenuchidae), *Curimatopsis evelynae* (Curimatidae), *Copella eigenmanni*, *Nannostomus anduzei* and *N. marilynae* (Lebiasinidae), *Corydoras crypticus*, *C. melini* (Callichthyidae), *Apistogramma uaupesi*, and *Dicrossus filamentosus* (Cichlidae) (Suppl. material 1).

A portion of the fish fauna recorded for the Rio Negro (89 species; 7.6%) is also known to occur in the clear water rivers of the Guiana Shield (Guyana, Suriname and French Guiana). Many were originally described for this region, and some also occur in the clear water rivers of the Brazilian Central Plateau. This group of species includes *Leporinus desmotes*, *L. nigrotaeniatus* (Anostomidae) *Moenkhausia hemigrammoides*, *M. lata* (Characidae), *Nannostomus beckfordi* (Lebiasinidae), *Corydoras potaroensis* (Callichthyidae) and *Mesonauta guyanae* (Cichlidae) (Suppl. material 1). Another 223 species (19.1%) are common inhabitants of Central Amazonian white water lowlands (várzeas), among them *Ilisha amazonica*, *Pellona castelnaeana* (Pristigasteridae), *Laemolyta proxima*, *Leporinus klausewitzi*, *Rhytiodus agenteofuscus*, *R. microlepis* (Anostomidae), *Brycon melanopterus* (Bryconidae), *Potamorhina altamazonica*, *P. latior*, *P. pristigaster*, *Psectrogaster rutiloides* (Curimatidae), *Semaprochilodus insignis*, *S. taeniurus*, *Prochilodus nigricans* (Prochilodontidae), *Anodus elongatus* (Hemiodontidae), *Mylossoma albiscopum*, *M. aureum*, *Metynnis luna* (Serrasalmidae), *Triportheus albus* (Triportheidae), *Aequidens pallidus*, *Geophagus altifrons*, *Satanoperca acuticeps*, *Uaru amphiacanthoides* (Cichlidae), and *Plagioscion montei* (Sciaenidae) (Suppl. material 1).

At least 61 species (5.2%) found in the Rio Negro are also distributed in tributaries of upper Amazonas near the Peruvian and Colombian borders with Brazil, indicating a possible zoogeographiocal relationship between these two parts of the Amazon Basin (cf. Ruokolainen et al. 2018), for example, *Hyphessobrycon erythrostigma*, *Petitella georgiae*, *Priocharax pygmaeus*, *Copeina guttata*, *Corydoras arcuatus*, *C. rabauti*, *Cetopsis parma*, *Myoglanis koepckei*, *Nemuroglanis lanceolatus*, and *Rhabdolichops nigrimans* (Suppl. material 1). Another 36 species (3.1%) have very wide geographic distribution, occurring also in the Paraná-Paraguay, La Plata and even the Rio São Francisco basins, i.e., *Pygocentrus nattereri*, *Serrasalmus maculatus* (Serrasalmidae), *Ossancora punctata*, *Oxydoras niger*, *Pterodoras granulosus* (Doradidae), *Sorubim lima*, *Sorubimichthys planiceps* (Pimelodidae), *Scoloplax dicra* (Scoloplacidae), *Pseudotylosurus angusticeps* (Belonidae), *Astronotus crassipinnis*, *Mesonauta festivus* (Cichlidae), and *Hoplerythrinus unitaeniatus* (Erytrinidae) also occurs in coastal drainages in southeastern Brazil and *Callichthys callichthys* (Callichthyidae) widespread in South America (Suppl. material 1).

Six species (0.5%) are exotic and invasive, possibly introduced by fish farmers, ornamental fish breeders, or even discarded by amateur aquarists in currently polluted streams draining the urban area of Manaus, in the lowermost stretch of Rio Negro near its confluence with Rio Solimões: *Danio rerio* (Southeast Asia), *Oreochromis niloticus* (Africa), *Poecilia reticulata* (northern South America), *Trichopodus trichopterus* (Southeast Asia), *Xiphophorus hellerii*, and *X. maculatus* (Central American countries).

Despite the presence of dozens of species identified only to genus-level in the surveyed lists, those could not be unambiguously considered as different taxonomic entities. Conversely, during this survey, representatives of 40 species representing unequivocally undescribed species were discovered among collection specimens and included in the present list, representing 3.4% of the listed species richness and probably do not represent an exact amount of undescribed species in the Rio Negro basin.

### Aquatic habitats and their fish faunas

Main river channel

**Figure 3.**
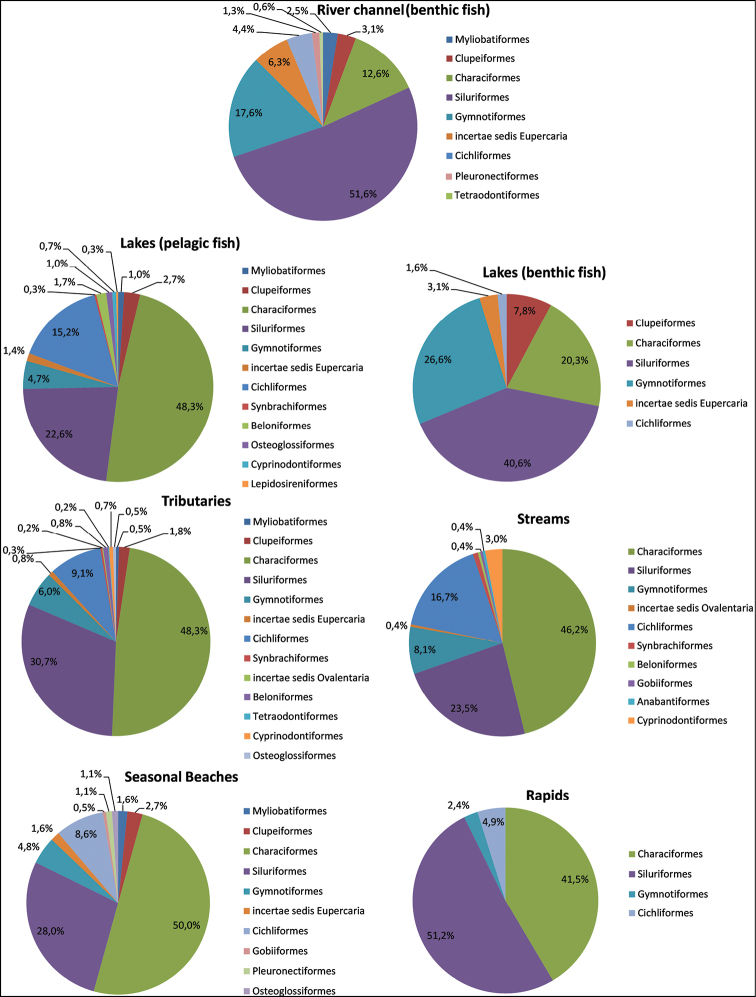
Taxonomic characterization (proportions of species by order) of fish assemblages found in different aquatic habitats of Rio Negro, Amazon Basin, Brazil.

The ichthyofauna of the main channel of the Rio Negro was accessed mostly by bottom trawl net sampling in six areas, including the mouth bay immediately upstream from Manaus, Rio Cuieiras, Rio Jufari, the confluence with Rio Branco, the lower Rio Branco, and nearby the city of Barcelos. The fish fauna is represented by at least 159 species distributed in 8 orders (+ the incertae sedis Eupercaria) and 24 families, mostly represented by benthic fishes. Siluriformes was dominant with 82 species (51.6%) in seven families, followed by Gymnotiformes with 28 species (17.6%) in three families, Characiformes with 20 species (12.6%) in six families, Eupercaria (Sciaenidae) with ten species (6.3%), and Cichliformes with seven species (4.4%) in one family. Four other orders were represented by 12 species (7.5%) (Figure 3, Suppl. material 1).

The most diverse genera in main channel of the Rio Negro were *Leptodoras* (7 species), *Eigenmannia*, *Rineloricaria*, *Trachydoras* (5 spp. each) *Pachyurus*, *Rabdolichops* (4 spp. each), *Hypophthalmus*, *Microschemobrycon*, *Plagioscion*, *Potamotrygon*, and *Sternarchella* (3 spp. each) (Suppl. material 1). Among the 159 species captured in the main channel, 18 were present in at least three of the six sampled areas: *Brachyplatystoma filamentosum*, *Hypophthalmus edentatus*, *Pimelodus blochii*, *Pimelodina flavipinnis*, *Platystomatichthys sturio*, *Pinirampus pirinampu* (Pimelodidae), *Pimelodella cristata* (Heptapteridae), *Pseudoloricaria laeviuscula*, *Pecklotia vittata* (Loricariidae), *Trachydoras microstomus* and *Opsodoras morei* (Doradidae), *Adontosternarchus clarkae*, *Sternarchogiton nattereri* and *S. porcinum* (Apteronotidae), *Steatogenys elegans* (Rhamphichthyidae), *Distocyclus conirostris* (Sternopygidae), *Pachypops fourcroi*, and *Pachyurus schomburgkii* (Sciaenidae).

Floodplain lakes

A total of 296 fish species (eleven orders + Eupercaria), distributed in 40 families, was sampled in five lakes of the lower Rio Negro, four lakes in the middle Rio Negro, and five lakes in the middle and lower Rio Branco. Representatives of the Characiformes were the richest with the largest number of families and species (17 families, 143 species, 48.3%), followed by Siluriformes (7 families, 67 species, 22.6%), Cichliformes (one family, 45 species, 15.2%), and Gymnotiformes (5 families, 14 species, 4.7%). Another seven orders (+ Eupercaria) were represented by nine families and 27 species (9.1%) (Figure 3, Suppl. material 1).

The lake fish fauna was generally sampled by gill nets that selectively catch medium- to large -sized fishes of both migratory and sedentary habits. However, small-sized fishes (88 species of up to 10 cm SL adult size) were sampled in the margins of the lakes, including representatives of the genera *Moenkhausia* (7 spp.), *Hemigrammus* (6 spp.), *Brynocops*, *Nannostomus* (4 spp. each), *Copella*, *Centromochlus*, *Tatia* (3 spp. each), and at least two species of dwarf cichlids (*Apistogramma* spp.) (Suppl. material 1).

Approximately 30 medium- to large-size species were captured in most sampled lakes: *Ilisha amazonica* and *Pellona flavipinnis* (Pristigasteridae), *Metynnis hypsauchen* (Serrasalmidae), *Boulengerella lucius* (Ctenolociidae), *Laemolyta taeniata* (Anostomidae), *Anodus elongatus*, *Hemiodus immaculatus* (Hemiodontidae), *Semaprochilodus insignis*, *S. taeniurus* (Prochilodontidae), *Triportheus angulatus* (Triportheidae), *Curimata vittata*, *Potamorhina altamazonica*, *P. latior* and *Cyphocharax abramoides* (Curimatidae), *Raphiodon vulpinus* (Cynodontidae), *Acestrorhynchus falcirostris* and *A. microlepis* (Acestrorhynchidae), *Serrasalmus rhombeus* and *S. elongatus* (Serrasalmidae), *Pimelodus blochii*, *Hypophthalmus edentatus*, *H. fimbriatus* and *H. marginatus* (Pimelodidae), *Ageneiosus lineatus* (Auchenipteridae), *Cichla temensis*, *Geophagus altifrons*, *G. proximus* and *Uaru amphiacanthoides* (Cichlidae), and *Plagioscion squamosissimus* (Scianidae).

Benthic ichthyofauna in a black water lake

So far, only one lake (Lago do Prato) in the lower Rio Negro basin has been thoroughly sampled for benthic fish using bottom trawl nets (Garcia (1993); specimens deposited in the INPA Fish Collection), resulting in 64 species in five orders (+ Eupercaria) and 20 families. Siluriformes was the richest order with six families and 26 species (40.6%), followed by Gymnotiformes (3 families and 17 species, 26.6%) and Characiformes (seven families and 13 species, 20.3%). Two other orders (Clupeiformes and Cichliformes) plus Eupercaria were represented by eight genera and eight species (Figure 3, Suppl. material 1). Siluriformes species (Pimelodidae: 11 spp., Doradidae: 7 spp.) and Gymnotiformes (Apteronotidae: 9 spp.; Sternopgidae: 7 spp.) were present in all periods of the hydrological cycle (Suppl. material 1).

Tributary rivers

Ichthyofaunal surveys and taxonomic inventories were made for the Jaú and Unini rivers (Jaú National Park), Carabinani and Paduari rivers (Rio Negro State Park – North Sector), Igarapé Tarumã-Açú near Manaus (all located in the lower Rio Negro), as well as for the rivers Branco, Jufari, Cuaru, Anapixi, Atauí, and some of the large tributaries of the middle Rio Negro (Igarapé Rei and Igarapé Zamula), and for the Rio Tiquié in the upper Rio Negro, resulting in 596 species. The ichthyofauna was dominated by species of Characiformes (17 families, 288 species; 48.3%), Siluriformes (12 families, 183 species, 30.7%), Cichliformes (one family, 54 species, 9.1%), and Gymnotiformes (5 families, 36 species, 6.0%) (Figure 3). Another seven orders (plus Eupercaria and Ovalentaria) were represented by ten families and 35 species (5.9%) (Suppl. material 1).

The most representative families in terms of species richness were: Characidae (103 species), Loricariidae and Cichlidae (54 each), Auchenipteridae (29), Serrasalmidae and Pimelodidae (28, each), and Anostomidae and Curimatidae (26 each), which corresponded to 58.5% of the species (Suppl. material 1). The most representative genera in these environments were: *Hemigrammus* (18 species), *Moenkhausia* (17), *Leporinus* (14), *Crenicichla* (13), *Hemiodus* (11), *Jupiaba* and *Corydoras* (10 spp. each), *Serrasalmus* (9), and *Characidium* (8), together representing 18.4% of the species found (Suppl. material 1).

Small upland forest streams

Two hundred thirty-four species belonging to seven orders of 36 families were recorded in streams. The ichthyofauna of this kind of environment is dominated by small Characiformes that correspond to 46.2% of the captured species (13 families, 108 species), followed by 23.5% of Siluriformes (10 families, 55 species), 16.7% of Cichliformes (one family, 39 species), and 8.1% of Gymnotiformes (5 families, 19 species), but also including some species (5.5%) of Cyprinodontiformes, Synbranchiformes, Gobiiformes, Anabantiformes, Beloniformes, and Ovalentaria (seven families and 13 species in total) (Figure 3 and Suppl. material 1). Some of the most common and/or abundant fishes in 1^st^ to 3^rd^ order streams include species of *Pyrrhulina*, *Copella* (Lebiasinidae), *Hyphessobrycon*, *Hemigrammus*, *Moenkhausia* (Characidae), *Bryconops* (Iguanodectidae), *Crenuchus*, *Characidium* (Crenuchidae), *Erythrinus*, *Hoplias* (Erythrinidae), *Helogenes* (Cetopsidae), *Hypopygus*, *Steatogenys* (Hypopomidae), *Aequidens*, *Apistogramma* (Cichlidae), and *Microphylipnus* (Eleotridae).

Seasonal beaches

Sampled beaches in Rio Branco and in the lower Rio Negro revealed the presence of 186 species distributed in eight orders and 33 families. The Characiformes were the richest group (16 families and 93 species, 50.0%), followed by Siluriformes (seven families and 52 species, 28.0%), Cichliformes (one family and 16 species, 8.6%), and Gymnotiformes (three families and nine species, 4.8%) (Figure 3). Five other orders (+ Eupercaria) were also present: Myliobatiformes, Osteoglossiformes, Clupeiformes, Gobiiformes, Pleuronectiformes, and Eupercaria (Sciaenidae) (Figure 3, Suppl. material 1). Some families stood out in terms of species richness in beaches: Characidae (38 spp.), Cichlidae (16 spp.), Hemiodontidae (12 spp.), Loricariidae and Trichomycteridae (11 spp. each), and Doradidae and Auchenipteridae (9 spp. each). The most representative genera in the beaches were *Moenkhausia* (10 species), *Hemiodus* (9), *Hemigrammus* (6), *Boulengerella*, *Bryconops*, and *Cyphocharax* (4 spp. each) (Suppl. material 1).

Rapids

In the rapids of the upper Rio Branco (middle Rio Negro) and Rio Tiquié (upper Rio Negro), 41 fish species of four orders and nine families were recorded. Siluriformes predominated (3 families and 21 species, 51.2%), followed by Characiformes (4 families and 17 species, 41.5%), Cichliformes (1 family and 2 species), and Gymnotiformes (1 family and 1 species) (Figure 3, Suppl. material 1). In those rapids the Loricariidae (10 species), Heptapteridae (9 spp.), Crenuchidae and Serrasalmidae (5 spp. each), and Anostomidae (4 spp.) were the richest families. The most representative genera found in the rapids were *Characidium* and *Peckoltia* (3 species each) (Suppl. material 1).

## Discussion

This study presents an extensive, updated compilation of occurrence records of fish species for the Rio Negro basin, one of the most important tributaries of the Rio Amazonas. Overall, 1,165 species have confirmed occurrences in the basin, and the estimated total fish richness may reach 1759 species. This remarkably high species richness is far from adequately known, and the rate of species descriptions for the basin does not show signs of stabilization.

The compiled species richness characterizes the Rio Negro ichthyofauna as one of the richest and most diversified in the Amazon Basin and in the world, shouldering the recently disclosed fish richness for the Rio Madeira in southwestern Brazilian Amazon (Queiroz et al. 2013). However, differently from the Rio Madeira, there were no concentrated efforts to survey the Rio Negro fish fauna, which points out to a potential increase of fish species records for the basin in the future. Moreover, there is a strong unbalance in the accumulated sampling effort (and, consequently, in the recorded amount of species) between the relatively well-sampled lower portion (between Manaus and the confluence with the Unini and Jaú rivers) and the mid and upper courses of the Rio Negro close to the borders with Colombia and Venezuela (pers. obs.). This knowledge and sampling effort discrepancies do not allow a more comprehensive comparison of fish assemblages among sub-regions of the basin.

Despite the wide distribution areas of many fishes in the basin, most species are confined to specific aquatic environments, such as the main channel of large rivers, tributary rivers of different water types, lakes, shallow banks and beaches, small upland forest streams, interfluvial swamps and rapids stretches. Analyzing the taxonomic composition of the fish fauna by environment, it was verified that ostariophysans dominates in most types of habitats, but with differences reflecting the ecological particularities of each group. In the deep waters of the main river channel, Siluriformes (catfishes) and Gymnotiformes (electric fishes) dominate the assemblages (Chao 2001; Thomé-Souza and Chao 2004; Ferreira et al. 2007; Rapp Py-Daniel et al. 2017). Thomé-Souza and Chao (2004), studying the ichthyofauna of the channels of the Negro and Branco rivers, verified that these environments presented differences in composition, species richness and abundance throughout the hydrological periods, which was presumed to be the result of predation and migration. The dominance of electric fishes was also observed in a large black water lake in the Anavilhanas archipelago surveyed by bottom trawl net samplings (Garcia 1993). Although little is known in terms of their natural history, representatives of the Gymnotiformes seem to represent a large biomass in deep river channels and to be of great importance as food source for many species of large catfish (Barthem and Goulding 1997; Barbarino-Duque and Winnemiller 2003; Cox-Fernandes et al. 2004).

In lakes and tributary rivers, migratory species of Characiformes and Siluriformes presented the highest frequency and abundance (Zuanon et al. 1998; Saint-Paul et al. 2000; Lima et al. 2005; Soares and Yamamoto 2005; Zuanon et al. 2008; Yamamoto et al. 2014). Many species occurring in these environments are also common and abundant in white water floodplains of the Solimões/Amazonas system, and in other tributaries of the Amazon Basin (Ferreira 1993; Rapp Py-Daniel et al. 2007; Lima and Caires 2011), and are of great commercial importance (Goulding 1980; Junk et al. 1983; Bayley 1983, 1998, Saint-Paul et al. 2000; Ruffino et al. 2006; Soares et al. 2007). In addition, many of the small-sized species recorded for lakes and tributaries are also of great importance for the ornamental fish trade, especially in the middle and upper Rio Negro (Chao et al. 2001; Anjos et al. 2009).

In small upland forest streams that are not influenced by the seasonal flood pulse, small characins and catfishes are common (Mendonça et al. 2005; Espírito-Santo et al. 2009; Zuanon et al. 2015), but their local abundance is usually low because of the low aquatic productivity of those oligotrophic streams (Anjos 2014). The fish communities that inhabit these environments are among the most diversified and least known in the Amazon, mainly due to difficulties of access to the such water bodies far inland in the forest. In general they are small species that usually do not exceed 10 cm in standard length. Those fishes, for the most part, do not make long migrations, and spend almost their entire life in the same system or habitats. Because they are primarily dependent on allochthonous forest material, these species are highly sensitive to changes in the surrounding environment (Lowe-McConnell 1987; Silva 1993; Walker 1995; Beltrão 2007; Beltrão et al. 2018).

Streams disturbed by deforestation and pollution in the urban areas of large cities such as Manaus are occupied by non-native species such as the tilapia *Oreochromis niloticus*, three-spot gourami *Trichopodus trichopterus*, guppy *Poecilia reticulata*, swordfishes *Xiphophorus hellerii* and *X. maculatus* and the zebra fish *Danio rerio* (Beltrão 2007). Allochthonous species from nearby white-water floodplains also invade such heavily polluted urban streams. Such species dominates or even completely replace the fish fauna in impacted streams, and investigations have found evidence of established populations, a situation not yet recorded in intact streams to date (Beltrão 2007).

### Water types, habitat diversity and hydrological connectivity

Historical and geomorphological factors certainly determined to a great extent the differences observed in the fish fauna within the sub-basins of the Amazon basin (Goulding et al. 1988; Lundberg et al. 1998; Albert et al. 2011; Dagosta and de Pinna 2017, 2019), but the relative importance of these factors still needs to be properly evaluated. However, ecological factors are also determinant to the composition and abundance of fish assemblages in any hydrographic system. Among the several factors that influence the structure of fish assemblages of the Amazon are the water type and the diversity of habitats (Junk et al. 1997; Lundberg et al. 1998). Blackwater rivers such as the Rio Negro show a very low autochthonous (aquatic) productivity, because they originate in extremely poor soils with low nutrient contents (Fittkau et al. 1975; Goulding et al. 1988; Worbes 1997; Küchlera et al. 2000), which results in low values of fish density and biomass (Saint-Paul et al. 2000). However, such low productivity does not imply in a low fish diversity, since black water rivers may have comparable or even higher fish diversity than white water systems of similar sizes (Goulding et al. 1988; Saint-Paul et al. 2000). Therefore, differences in water chemistry, sediment loads and nutrient contents among the tributaries of the Rio Negro add heterogeneity to the basin and allow the coexistence of a huge diversity of fishes in a basin-scale (e.g., Ferreira et al. 2007).

The high fish diversity in the Rio Negro basin can also be explained by the enormous variety of habitats available for fish, such as the flooded forests (igapós), fluvial channels, lakes, streams, marginal ponds, temporary beaches, rapids, waterfalls and various types of interfluvial swamps. In the middle Rio Negro, for example, there are large floodplains located between the cities of Barcelos and Santa Isabel do Rio Negro as well as in the lower portion of the Rio Branco basin, in Roraima State. These extensive interfluvial regions become flooded during the rainy seasons and, in some areas, may remain flooded even during periods of extreme drought, due to the high level of the water table and the presence of hydromorphic soils (Fittkau et al. 1975; Goulding et al. 1988; Marshall et al. 2011; Vale et al. 2014). These areas harbor many species of small fish, several of them of great importance for the ornamental fish trade (Chao et al. 2001; Anjos et al. 2009).

The connectivity of the Rio Negro with large adjacent basins is another factor that contributes to the high richness in the basin. In fact, due to its peculiar geographic position, the Rio Negro has been historically interconnected to other rivers with different types of water (Winemiller and Willis 2011), which may constitute (or have constituted) important ichthyofaunistic interchange zones between basins. A preliminary analysis of the distribution of the species present in three adjacent basins (Rio Negro – Rio Orinoco – Rio Branco-Essequibo River in Guiana shield) reveals that at least 227 species are shared among these basins, indicating the importance of historical or current hydrological connectivity for the ichthyofaunal similarity of these basins. One of the most representative instances is the current connection with the Orinoco River by the Casiquiare and other adjacent channels (Winemiller et al. 2008; Winemiller and Willis 2011). Other drainage connections have been pointed out, involving clear rivers of the Guiana Shield through the Rio Branco/Rupununi interfluvial plains (Ferreira et al. 2007; Winemiller and Willis 2011), as well as white water rivers of the upper Amazonas, such as the Caquetá-Japurá rivers through headwater capture events in the past (Ruokolainen et al. 2018). The headwaters of the Rio Quiuini, a tributary of the middle Rio Negro, in periods of great floods present turbid waters possibly due to the overflow of white water from the Rio Japurá (H. Beltrão, personal observation), which may constitute another active route for fish interchange among basins. Other possible connections exist between the rivers that flow to the Lago Amanã in the middle Rio Solimões/Amazonas and the headwaters of the Rio Unini in the right bank of the lower Rio Negro (Zuanon et al. 1998). These different connections possibly have helped maintaining - and may have incremented - the fish diversity of the Rio Negro basin.

Dagosta and de Pinna (2017) analyzing the biogeography of fishes in sub-basins of the Amazon Basin, considered the Rio Negro and Rio Branco as two distinct biogeographic regions, despite the latter constituting a tributary of the former. In fact, of the 156 species considered exclusive to the Rio Negro, 115 occur only in that river, 29 only in Rio Branco, and just 12 are shared by the two drainage basins. However, nearly 65% of the species that occur in the Rio Branco are shared with the Rio Negro basin.

There is a direct relation between a river basin area and its fish species richness (Hugueny et al. 2010), which is supported by the general species-area theory (e.g., Rosenzweig 1995). However, the Rio Negro basin shows a species richness that is much higher than expected by its drainage area, holding the greatest fish species richness (1,165 spp.) so far recorded for a river in the Brazilian territory and surpassing the 1,062 species recorded for the Rio Madeira (Dagosta and de Pinna 2019). It’s noteworthy that such huge fish diversity is contained in an area of 696,808 Km^2^ which corresponds to half of the area of the Rio Madeira basin (1,380,000 km^2^). The Rio Negro fish richness is remarkably higher than that of the Rio Tocantins basin with 757,000 km^2^ and 705 species (Lima and Caires 2011; Dagosta and de Pinna 2019), and also proportionally much higher than that of the Rio Xingu basin with 504,277 Km^2^ and 502 species (Camargo et al. 2004; Dagosta and de Pinna 2019). However, it should be noted that differences in the accumulated sampling effort may partially explain the remarkably lower species richness recorded for some of the above-mentioned basins. Conversely, the fish richness in the Rio Negro basin is comparable to that of the Rio Orinoco basin in Venezuela with 1,002 species (Reis et al. 2016) but for a basin area of 1,212,000 km^2^.

Besides the high number species richness, some taxonomic groups apparently show a higher rate of endemism in Rio Negro than in other adjacent drainage basins. For example, 17 of the 31 species of *Corydoras* and 12 of 20 species of *Apistogramma* found in Rio Negro are endemic. Other examples are the cyprinodontiform genera *Anablepsoides* and *Laimosemion*, with 13 species found in Rio Negro and at least 10 endemic species. Representatives of these groups generally are small-sized species that are sedentary and with low active dispersal capacity (such as the small Characidae, Callichthyidae and Loricariidae) or display strong territorial behavior (i.e., small cichlids of the genus *Apistogramma*). These characteristics may favor speciation events at relatively small spatial scales (e.g., de Oliveira et al. 2009), resulting in discontinuous patterns of occurrence and possibly contributing to a high endemism rate in the Rio Negro basin.

### Final considerations and perspectives

The results presented in this compilation show that in recent years considerable progress has been achieved regarding the knowledge of the fish diversity in the Rio Negro basin, but also point out to important information gaps that still need to be addressed. It is necessary to investigate the taxonomic identity of many forms with uncertain status and, in the case of new species, to describe them, which may still take a long time (e.g., Ota et al. 2015). This may allow the identification of rare or threatened species that will eventually need protection policies.

Additionally, the fish diversity in hard-to-reach areas in the basin should be better sampled. Studies in the upper Rio Negro (Lima et al. 2005) revealed the presence of taxa that had never been found in Rio Negro (i.e., representatives of *Tometes* and *Utiaritichthys*; Serrasalmidae) and the discovery of at least 30 possible new species, several of which were eventually described (Britto and Lima 2003; Zanata and Lima 2005; Ferreira and Lima 2006; Lima et al. 2009; Marinho and Lima 2009; Lima and Sousa 2009; Lima and Soares 2018; Lehmann et al. 2018). Research should be encouraged in the regions of the upper and middle Rio Negro, particularly in the smaller tributaries near the borders between Brazil, Venezuela and Colombia.

The list of 1,165 fish species presented herein for the Rio Negro basin represents a 259% increase in the number of species documented by Goulding et al. (1988) (450 species). This improvement in knowledge resulted from an increase of the ichthyofaunal surveys along the Rio Negro basin, but also from an accelerated rate of species descriptions in recent years. Despite this improvement in the knowledge about the fish fauna in the basin. A considerable number of species still await to be inventoried, discovered or formally described, as already pointed out by Bohlke et al. (1978), Ota et al. (2015) and Birindelli and Sidlauskas (2018) regarding the neotropical ichthyofauna.

Information about the Rio Negro basin and its biota has been gathered for ca. 230 years, since the pioneer naturalists reports by Alexandre Rodrigues Ferreira (1784–87), Alexander Von Humboldt (1800) and Alfred Russel Wallace (1850–52). Samples that have been deposited in collections and/or museums for many years and that were not properly identified were recently described as new species (e.g., *Leptophilypnion fittkaui* Robert, 2013 and *Polycentrus jundia* Coutinho & Wosiacki, 2014). Revision of many genera or problematic groups (e.g., *Moenkhausia*) have revealed species still unknown to science, including some occurring in Rio Negro (Marinho 2009; Marinho et al. 2015). More recently, a new fish family (Tarumaniidae) has been discovered in a stream of the lower Rio Negro (Igarapé Tarumã-Mirim), very close to Manaus, the largest city in the Brazilian Amazon, *Tarumania walkeri* de Pinna, Zuanon, Rapp Py-Daniel & Petry, 2017, a very surprising discovery for an ichthyofauna that has been surveyed for more than two centuries.

Although the Rio Negro basin is apparently very well preserved (< 1% of its area is deforested, accumulated in 2000–2013; Ricardo et al. 2015), environmental changes due to human activities, such as deforestation, habitat loss, pollution and introduction of non-native species, may jeopardize the rich fish fauna of this drainage basin, as reported for several freshwater ecosystems around the world (Revenga et al. 1998, 2005; Revenga and Kura 2003; Dudgeon et al. 2006). Therefore, concentred conservation efforts should be directed to the Rio Negro basin and its biota ahead of anthropic action, preserving one of the most species-rich and environmentally unique areas of the planet.

This study honors the late Dr. Javier Alejandro Maldonado-Ocampo, a very good friend and former Subject Editor of ZooKeys, for his important contribution to the knowledge of the fish diversity, taxonomy and evolution in the Amazon basin.

## Supplementary materials

Supplementary material 1 Checklist of the species found in the different environments of the Rio Negro basin Data type: species data https://binary.pensoft.net/file/347770This dataset is made available under the Open Database License (http://opendatacommons.org/licenses/odbl/1.0/). The Open Database License (ODbL) is a license agreement intended to allow users to freely share, modify, and use this Dataset while maintaining this same freedom for others, provided that the original source and author(s) are credited.Hélio Beltrão, Jansen Zuanon, Efrem Ferreira
