# Supplementary material for: Checklist of the ichthyofauna of the Rio Negro basin in the Brazilian Amazon
Source: Zookeys. 2019 Oct 17;881:53–89. doi: 10.3897/zookeys.881.32055 (PMC6813176; doi:10.3897/zookeys.881.32055)
Supplement: Supplementary material 1 [file zookeys-881-053-s001.docx]

**Table S1**. Checklist of the species found in the different environments of the Rio Negro basin. The list includes the sampled environments indicated with A = Main channel (benthic fish), B = Lakes (pelagic fish), C = Lakes (benthic fish), D = Tributaries, E = Streams, F = Beaches, G = Rapids and H = Compilation of Species + New descriptions of Taxon (> 2003-2019). In addition, the main reference, voucher of the species deposited mainly in INPA and MZUSP fish collections, and distribution of species (including endemic and exotic species introduced in streams in the Manaus region) are presented. Distribution key (river basins): Amaz = Amazonas; Gui = coastal rivers of the Guiana Shield; LaP = La Plata; Mad = Madeira; Neg = Negro; Neg(Bra) = only Branco; Ori = Orinoco; Par = Paraná; Pur = Purus; SaF = São Francisco; Tap = Tapajós; Tef = Rio Tefé ; Toc = Tocantins; Tro = Trombetas; Uat = Uatumã; Xin = Xingu; Cost Bra = Brazilian Coast; mid = middle; upp = upper and low = lower.

| **COHORT/SERIES/ORDER/Family/Species** | **A** | **B** | **C** | **D** | **E** | **F** | **G** | **H** | **Main Reference** | **Voucher number** | **Distribution** |
| --- | --- | --- | --- | --- | --- | --- | --- | --- | --- | --- | --- |
| **ELASMOBRANCHII** |  |  |  |  |  |  |  |  |  |  |  |
| **MYLIOBATIFORMES** |  |  |  |  |  |  |  |  |  |  |  |
| **Potamotrygonidae** |  |  |  |  |  |  |  |  |  |  |  |
| *Paratrygon aiereba* (Müller & Henle, 1841) | A |  |  | D |  |  |  |  | Ferreira et al. 2007 | INPA- 1775; 33951 | Amaz/Ori/Gui |
| *Plesiotrygon iwamae* Rosa, Castello & Thorson, 1987 |  |  |  |  |  |  |  | H | INPA Fish Collection | INPA- 35689 | Amaz main channel/Mad/Neg |
| *Potamotrygon adamastor* Fontenelle & Carvalho, 2017 |  |  |  |  |  |  |  | H | Fontenelle and Carvalho 2017 | MZUSP- 104662 | Neg (Bra) |
| *Potamotrygon amazona* Fontenelle & Carvalho, 2017 |  |  |  |  |  |  |  | H | Fontenelle and Carvalho 2017 | INPA- 7911 | Neg/mid Amaz |
| *Potamotrygon motoro* (Müller & Henle, 1841) | A | B |  | D |  | F |  |  | Rapp Py-Daniel et al. 2017 | INPA- 15099; 27089 | Amaz/Ori/Gui |
| *Potamotrygon orbignyi* (Castelnau, 1855) | A | B |  |  |  | F |  |  | Rapp Py-Daniel et al. 2017 | INPA- 27085; 27088 | Amaz/Ori/Gui |
| *Potamotrygon schroederi* Fernández-Yépez, 1958 |  |  |  |  |  |  |  | H | Reis et al. 2003 | INPA- 33964; 37081 | Neg/Ori/Amaz main channel |
| *Potamotrygon scobina* Garman, 1913 | A |  |  |  |  | F |  |  | Ferreira et al. 2007 | INPA- 33956; 33961 | Amaz/Ori/Gui |
| *Potamotrygon wallacei* Carvalho, Rosa & Araújo, 2016 |  | B |  | D |  |  |  | H | Carvalho et al. 2016 | INPA- 15108; 33959 | Neg |
| **TELEOSTEI** |  |  |  |  |  |  |  |  |  |  |  |
| **OSTEOGLOSSOMORPHA** |  |  |  |  |  |  |  |  |  |  |  |
| **OSTEOGLOSSIFORMES** |  |  |  |  |  |  |  |  |  |  |  |
| **Arapaimidae** |  |  |  |  |  |  |  |  |  |  |  |
| *Arapaima gigas* (Schinz, 1822) |  | B |  | D |  |  |  |  | Goulding et al. 1988 | INPA- 1843 | Amaz/Ori/Gui |
| **Osteoglossidae** |  |  |  |  |  |  |  |  |  |  |  |
| *Osteoglossum bicirrhosum* (Cuvier, 1829) |  | B |  | D |  | F |  |  | Goulding et al. 1988 | INPA- 6541; 35549 | Amaz/Ori/Gui |
| *Osteoglossum ferreirai* Kanazawa, 1966 |  | B |  | D |  | F |  |  | Zuanon et al. 2008 | INPA- 30689; 37857 | Neg |
| **CLUPEOCEPHALA** |  |  |  |  |  |  |  |  |  |  |  |
| **CLUPEIFORMES** |  |  |  |  |  |  |  |  |  |  |  |
| **Engraulidae** |  |  |  |  |  |  |  |  |  |  |  |
| *Amazonsprattus scintilla* Roberts, 1984 | A |  |  | D |  | F |  |  | Goulding et al. 1988 | INPA- 41128 | Neg/Xin/Tap/Mad/Uat |
| *Anchovia surinamensi*s (Bleeker, 1865) |  | B | C | D |  |  |  |  | Zuanon et al. 1998 | INPA- 10273; 10287 | Amaz/Ori/Gui |
| *Anchoviella carrikeri* Fowler, 1940 | A | B |  | D |  | F |  |  | Loeb 2012 | INPA- 13142 | Amaz/Ori/Gui |
| *Anchoviella guianensis* (Eigenmann, 1912) |  |  |  | D |  |  |  |  | Ferreira et al. 2007 | MZUSP- 11598 | Amaz/Ori/Gui |
| *Anchoviella jamesi* (Jordan & Seale, 1926) |  | B |  | D |  | F |  |  | Goulding et al. 1988 | INPA- 13180 | Amaz/Ori/Gui |
| *Anchoviella juruasanga* Loeb, 2012 |  |  |  |  |  | F |  | H | Loeb 2012 | INPA- 17905 | Amaz/Ori/Gui |
| *Anchoviella lepidentostole* (Fowler, 1911) |  |  |  |  |  |  |  | H | INPA Fish Collection | INPA- 51887 | Amaz/Ori/Gui/Cost Bra |
| *Lycengraulis batesii* (Günther, 1868) |  | B | C | D |  | F |  |  | INPA Fish Collection | INPA- 10259; 10316 | Amaz/Ori/Gui |
| **Pristigasteridae** |  |  |  |  |  |  |  |  |  |  |  |
| *Ilisha amazonica* (Miranda-Ribeiro, 1920) |  | B | C | D |  |  |  |  | Saint-Paul et al. 2000 | INPA- 25793; 26536 | Amaz/Neg |
| *Pellona castelnaeana* Valenciennes, 1847 | A | B |  | D |  |  |  |  | Goulding et al. 1988 | INPA- 16251; 25778 | Amaz/Neg |
| *Pellona flavipinnis* (Valenciennes, 1837) | A | B | C | D |  |  |  |  | Saint-Paul et al. 2000 | INPA- 10294; 31403 | Amaz/Ori/Gui/Par |
| *Pristigaster cayana* Cuvier, 1829 | A | B | C | D |  |  |  |  | Thomé-Souza and Chao 2004 | INPA- 10261; 11335 | Amaz/Neg |
| *Pristigaster whiteheadi* Menezes & de Pinna, 2000 |  |  |  | D |  |  |  |  | Ferreira et al. 2007 | INPA- 26538; 36538 | Amaz/Neg |
| **OSTARIOPHYSI** |  |  |  |  |  |  |  |  |  |  |  |
| **CYPRINIFORMES (NON-NATIVE)** |  |  |  |  |  |  |  |  |  |  |  |
| **Cyprinidae** |  |  |  |  |  |  |  |  |  |  |  |
| *Danio rerio* (Hamilton, 1822) |  |  |  |  |  |  |  | H | UFAM Fish Collection | UFAM- 0286 | Non-native (Manaus) |
| **CHARACIFORMES** |  |  |  |  |  |  |  |  |  |  |  |
| **Acestrorhynchidae** |  |  |  |  |  |  |  |  |  |  |  |
| *Acestrorhynchus falcatus* (Bloch, 1794) |  | B |  | D | E |  |  |  | Lima et al. 2005 | UFAM- 0198 | Amaz/Ori/Gui |
| *Acestrorhynchus falcirostris* (Cuvier, 1819) |  | B |  | D |  | F |  |  | Zuanon et al. 2008 | INPA- 2658 | Amaz/Ori/Gui |
| *Acestrorhynchus grandoculis* Menezes & Géry, 1983 |  |  |  | D |  | F |  |  | Zuanon et al. 1998 | INPA- 5219; 39324 | Neg/Ori |
| *Acestrorhynchus heterolepis* (Cope, 1878) |  | B |  | D |  |  |  |  | Rapp Py-Daniel et al. 2017 | UFAM- 0126; 0138 | Amaz/Ori |
| *Acestrorhynchus maculipinna*Menezes & Géry, 1983 |  |  |  |  |  |  |  | H | INPA Fish Collection | INPA-51580 | Amaz main channel/Neg |
| *Acestrorhynchus microlepis* (Jardine, 1841) |  | B | C | D |  | F |  |  | Zuanon et al. 2008 | MZUSP- 29266 | Amaz/Ori |
| *Acestrorhynchus minimus* Menezes, 1969 |  |  |  | D |  |  |  |  | Goulding et al. 1988 | INPA- 15227 | Amaz/Ori |
| *Acestrorhynchus nasutus* Eigenmann, 1912 |  | B |  | D | E |  |  |  | Rapp Py-Daniel et al. 2017 | INPA- 15235 | Amaz/Ori |
| *Gnathocharax steindachneri* Fowler, 1913 |  |  |  | D | E |  |  |  | Zuanon et al. 2015 | INPA- 15932; 27705 | Amaz/Ori/Gui |
| *Heterocharax leptogrammus* Toledo-Piza, 2000 |  |  |  |  |  |  |  | H | Lucena and Menezes 2003 | MZUSP- 55727; 55725 | Neg/Ori |
| *Heterocharax macrolepis* Eigenmann, 1912 |  | B |  | D | E |  |  |  | Rapp Py-Daniel et al. 2017 | INPA- 12875; 42826 | Amaz/Ori/Gui |
| *Heterocharax virgulatu*s Toledo-Piza, 2000 |  |  |  | D | E |  |  | H | Lucena and Menezes 2003 | INPA- 42827 | Amaz/Ori |
| *Hoplocharax goethei* Géry, 1966 |  |  |  | D | E |  |  |  | Goulding et al. 1988 | INPA- 26266; 30439 | Amaz River basin |
| *Lonchogenys ilisha* Myers, 1927 | A | B |  | D |  | F |  |  | Zuanon et al. 1998 | INPA- 12574; 15549 | Neg/Ori |
| *Roestes ogilviei* (Fowler, 1914) |  |  |  |  |  |  |  | H | Toledo-Piza 2003 | MZUSP- 52065; 52066 | Neg/Gui/upp Amazon |
| **Anostomidae** |  |  |  |  |  |  |  |  |  |  |  |
| *Abramites* *hypselonotus* (Günther, 1868) |  |  |  |  |  |  |  | H | INPA Fish Collection | INPA- 10978 | Amaz/Ori/Gui/Par |
| *Anostomoides laticeps* (Eigenmann, 1912) | A |  |  | D |  |  |  |  | Zuanon et al. 2008 | INPA- 11746; 11904 | Amaz/Ori/Gui |
| *Anostomus anostomus* (Linnaeus, 1758) |  |  |  | D | E |  | G |  | Lima et al. 2005 | INPA- 15179 | Amaz/Ori/Gui |
| *Anostomus ternetzi* Fernández-Yépez, 1949 |  |  |  | D | E |  |  |  | Goulding et al. 1988 | INPA- 8139 | Amaz/Ori/Gui |
| *Hypomasticus julii* (Santos, Jégu & Lima, 1996) |  |  |  |  |  |  |  | H | INPA Fish Collection | INPA- 14969 | Neg/Xin |
| *Hypomasticus megalepis* (Günther, 1863) |  |  |  |  | E |  | G |  | Ferreira et al. 2007 | INPA- 1301 | Neg/Gui |
| *Laemolyta garmani* (Borodin, 1931) |  |  |  |  |  |  |  | H | Mautari and Menezes 2006 | MZUSP- 63614 | Amaz/Neg |
| *Laemolyta proxima* (Garman, 1890) | A | B |  | D |  | F |  | H | Mautari and Menezes 2006 | INPA- 35674; 35998 | Amaz/Neg |
| *Laemolyta taeniata* (Kner, 1858) |  | B |  | D |  | F |  | H | Mautari and Menezes 2006 | INPA- 12070; 13134 | Amaz/Ori |
| *Leporellus vittatus* (Valenciennes, 1850) |  |  |  | D |  |  |  |  | Ferreira et al. 2007 | INPA- 13549; 14047 | Amaz/Ori/Gui |
| *Leporinus agassizi* Steindachner, 1876 |  | B |  | D |  |  |  |  | Ferreira et al. 2007 | INPA- 10544; 10548 | Amaz/Ori |
| *Leporinus altipinnis* Borodin, 1929 |  | B |  | D |  |  |  | H | Britski and Birindelli 2016 | INPA- 1174; 1184 | Neg/Ori/Tap |
| *Leporinus amazonicus* Santos & Zuanon, 2008 |  |  |  |  |  |  |  |  | INPA Fish Collection | INPA- 22125; 22860 | Amaz/Neg |
| *Leporinus arimaspi* Burns, Frable & Sidlauskas, 2014 |  | B |  | D | E |  |  |  | Burns et al. 2014 | CAS- 20129 | Neg (Bra)/Ori |
| *Leporinus bleheri* Géry, 1999 |  |  |  |  |  |  |  | H | Burns et al. 2017 | MZUSP- 66676; 93445 | Amaz/Neg |
| *Leporinus brunneus* Myers, 1950 |  |  |  | D |  |  |  |  | Ferreira et al. 2007 | INPA- 4929; 14148 | Neg/Ori/Tap |
| *Leporinus cylindriformes* Borodin, 1929 |  |  |  | D |  |  |  | H | INPA Fish Collection | INPA- 6612 | Amaz/Neg |
| *Leporinus desmotes* Fowler, 1914 |  |  |  | D | E |  | G |  | Goulding et al. 1988 | INPA- 10570; 43068 | Neg/Gui |
| *Leporinus enyae* Burns, Chatfield, Birindelli & Sidlauskas, 2017 |  |  |  |  |  |  |  | H | Burns et al. 2017 | INPA- 3180; 4930 | Neg/Ori |
| *Leporinus fasciatus* (Bloch, 1794) | A | B |  | D |  | F |  | H | Birindelli and Britski 2009 | INPA- 1180; 13486 | Amaz/Ori/Gui |
| *Leporinus friderici* (Bloch, 1794) |  | B |  | D |  |  |  |  | Lima et al. 2005 | INPA- 14151; 15593 | Amaz/Ori/Gui |
| *Leporinus granti* Eigenmann, 1912 |  |  |  | D |  |  |  |  | Ferreira et al. 2007 | INPA- 10549 | Neg/Gui |
| *Leporinus klausewitzi* Géry, 1960 |  |  |  | D | E |  |  |  | Ferreira et al. 2007 | INPA- 11744; 14152 | Amaz/Neg |
| *Leporinus maculatus* Müller & Troschel, 1844 |  |  |  | D |  |  |  |  | Ferreira et al. 2007 | INPA- 1622; 14862 | Amaz/Ori/Gui |
| *Leporinus nattereri* Steindachner, 1876 |  |  |  | D |  |  |  |  | INPA Fish Collection | INPA- 14042 | Amaz/Neg/Ori |
| *Leporinus nigrotaeniatus* (Jardine, 1841) |  |  |  | D |  | F |  | H | Garavello and Britski 2003 | INPA- 10563; 10565 | Neg/Gui |
| *Leporinus ortomaculatus* Garavello, 2000 |  |  |  | D |  |  |  | H | INPA Fish Collection | INPA- 10553; 13534 | Neg (Bra)/Ori |
| *Megaleporinus trifasciatus* Steindachner, 1876 |  |  |  |  |  |  |  | H | Birindelli and Britski 2009 | INPA- 25798 | Amaz/Ori/Gui |
| *Petulanos plicatus* (Eigenmann, 1912) |  |  |  | D | E |  |  |  | Ferreira et al. 2007 | INPA- 10088 | Neg/Gui |
| *Pseudanos gracilis* (Kner, 1858) |  | B |  | D |  |  | G | H | Birindelli et al. 2012 | INPA- 11978; 13524 | Amaz/Neg |
| *Pseudanos varii* Birindelli, Lima & Britski, 2012 |  |  |  |  |  |  |  | H | Birindelli et al. 2012 | INPA- 15247 | Neg/Ori |
| *Pseudanos trimaculatus* (Kner, 1858) |  | B |  | D | E |  |  | H | Birindelli et al. 2012 | INPA- 11745; 11816 | Amaz/Ori/Gui |
| *Rhytiodus argenteofuscus* Kner, 1858 |  | B |  | D |  |  |  |  | Goulding et al. 1988 | INPA- 13537 | Amaz/Neg |
| *Rhytiodus microlepis* Kner, 1858 |  | B |  |  |  |  |  |  | INPA Fish Collection | INPA- 21604; 25653 | Amaz/Neg |
| *Schizodon fasciatus* Spix & Agassiz, 1829 |  | B |  | D |  |  |  |  | Goulding et al. 1988 | INPA- 11973; 13453 | Amaz/Ori/Gui |
| *Synaptolaemus latofasciatus* (Steindachner, 1910) |  |  |  | D |  |  |  |  | Ferreira et al. 2007 | INPA- 10571; 10573 | Amaz/Ori |
| **Bryconidae** |  |  |  |  |  |  |  |  |  |  |  |
| *Brycon amazonicus* (Spix & Agassiz, 1829) |  | B |  | D |  |  |  |  | Zuanon et al. 2008 | INPA- 15925; 39327 | Amaz/Ori/Gui |
| *Brycon falcatus* Müller & Troschel, 1844 |  | B |  | D | E |  |  |  | Goulding et al. 1988 | INPA- 16388; 16394 | Amaz/Ori/Gui |
| *Brycon melanopterus* (Cope, 1872) |  | B |  | D |  |  |  |  | Lima et al. 2017 | INPA- 5527 | Amaz/Neg |
| *Brycon pesu* Müller & Troschel, 1845 |  | B |  | D | E | F |  |  | Lima et al. 2005 | INPA- 42773; 43033 | Amaz/Ori/Gui |
| *Salminus hilarii* Valenciennes, 1850 |  |  |  | D |  |  |  |  | Ferreira et al. 2007 | INPA- 10791 | Amaz/Ori/Gui |
| **Chalceidae** |  |  |  |  |  |  |  |  |  |  |  |
| *Chalceus epakros* Zanata & Toledo-Piza, 2004 |  | B |  | D | E |  |  |  | Zuanon et al. 2008 | INPA- 30683 | Amaz/Ori/Gui |
| *Chalceus erythrurus* (Cope, 1870) |  |  |  |  |  |  |  | H | Zanata and Toledo-Piza 2004 | INPA 16949 | Amaz/Neg |
| *Chalceus macrolepidotus* Cuvier, 1818 |  | B |  | D |  |  |  |  | Lima et al. 2005 | INPA- 35760 | Amaz/Ori/Gui |
| **Characidae** |  |  |  |  |  |  |  |  |  |  |  |
| *Acestrocephalus sardina* (Fowler, 1913) | A |  |  | D |  | F |  | H | Menezes 2006 | INPA- 42770 | Amaz/Ori/Gui |
| *Aphyocharax alburnus* (Günther, 1869) |  |  |  | D |  | F |  |  | Ferreira et al. 2007 | INPA- 36939; 36853 | Amaz/Ori/Gui |
| *Aphyocharax avary*Fowler, 1913 |  |  |  |  |  |  |  | H | INPA Fish Collection | INPA-53042 | Mad/Neg |
| *Aphyocharax erythrurus* Eigenmann, 1912 |  |  |  | D |  |  |  |  | Ferreira et al. 2007 | INPA- 7872 | Neg (Bra)/Gui |
| *Aphyodite grammica* Eigenmann, 1912 |  | B |  | D |  |  |  | H | Esguícero and Castro 2017 | MZUSP- 29873; 29874 | Neg/Gui |
| *Astyanax ajuricaba* Marinho & Lima, 2009 |  |  |  |  |  |  |  | H | Marinho and Lima 2009 | INPA- 31196 | Neg/Toc/Tap/Amaz main channel |
| *Astyanax bimaculatus* (Linnaeus, 1758) |  | B |  | D | E |  |  |  | Ferreira et al. 2007 | INPA- 36952; 43791 | Amaz/Ori/Gui |
| *Astyanax clavitaeniatus* Garutti, 2003 |  |  |  | D |  |  |  |  | Ferreira et al. 2007 | MZUSP- 48281 | Neg (Bra) |
| *Astyanax anterior* Eigenmann, 1908 |  |  |  | D |  |  |  |  | Kemenes and Forsberg 2014 | MZUSP- 16959 | Neg/upp Amaz |
| *Astyanax guianensis* Eigenmann, 1909 |  |  |  | D |  |  |  | H | Marinho et al. 2015 | INPA- 50922; 51070 | Amaz/Ori/Gui |
| *Astyanax venezuelae* Schultz, 1944 |  |  |  |  |  |  |  | H | MZUSP Fish Collection | MZUSP- 55193 | Neg/Ori |
| *Atopomesus pachyodus* Myers, 1927 |  |  |  | D |  | F |  | H | Esguícero and Castro 2016 | INPA- 12650; 43005 | Neg/Ori |
| *Axelrodia lindeae* Géry, 1965 |  |  |  |  | E |  |  | H | Marinho et al. 2013 | INPA- 26832 | Neg/Tap/Mad/Amaz main channel |
| *Axelrodia riesei* Géry, 1966 |  |  |  |  |  |  |  | H | INPA Fish Collection | INPA- 40935; 4399 | Neg/Ori |
| *Axelrodia stigmatias* (Fowler, 1913) |  |  |  |  |  |  |  | H | INPA Fish Collection | INPA- 37843 | Amaz/Neg/Mad |
| *Brachychalcinus orbicularis* (Valenciennes, 1850) |  |  |  | D |  |  |  |  | Goulding et al. 1988 | INPA- 10757; | Neg/Gui |
| *Brittanichthys axelrodi* Géry, 1965 |  | B |  | D | E |  |  |  | Goulding et al. 1988 | INPA- 15417; 20981 | Neg/Ori |
| *Brittanichthys myersi* Géry, 1965 |  |  |  | D | E |  |  |  | Rapp Py-Daniel et al. 2017 | INPA- 39401 | Neg |
| *Bryconamericus* *diaphanus* (Cope, 1878) |  |  |  | D |  |  |  |  | Ferreira et al. 2007 | INPA- 36850 | Amaz/Neg |
| *Bryconamericus macrophthalmus*Román-Valencia, 2003 |  |  |  |  |  |  |  | H | Román-Valencia 2003 | MBUC- 29392 | Neg/Ori |
| *Bryconamericus orinocoensis*Román-Valencia, 2003 |  |  |  |  |  |  |  | H | INPA Fish Collection | INPA-49465; 49792 | Ori/Neg/Tap/Mad |
| *Bryconamericus ternetzi* Eigenmann, 1908 |  |  |  |  |  |  |  | H | Reis et al. 2003 | CAS- 44216 | Neg/Ori/Amaz |
| *Bryconella pallidifrons*(Fowler, 1946) |  |  |  |  |  |  |  | H | INPA Fish Collection | INPA- 36927 | Amaz/Neg |
| *Charax condei* (Géry & Knöppel, 1976) |  |  |  | D |  |  |  |  | Rapp Py-Daniel et al. 2017 | INPA- 39484 | Amaz River basin |
| *Charax delimai* Menezes & Lucena, 2014 |  |  |  |  |  |  |  | H | Menezes and Lucena 2014 | MZUSP- 81505; 84988 | Neg |
| *Charax gibbosus* (Linnaeus, 1758) |  | B |  | D |  | F |  |  | Goulding et al. 1988 | INPA- 12654 | Neg/Gui |
| *Charax hemigrammus* (Eigenmann, 1912) |  |  |  |  |  |  |  | H | Menezes and Lucena 2014 | MZUSP- 100354 | Neg/Gui/Amaz main channel |
| *Charax macrolepis* (Kner, 1858) |  |  |  |  |  |  |  | H | Menezes and Lucena 2014 | MZUSP- 62233; 92392 | Amaz |
| *Charax michaeli* Lucena, 1989 |  |  |  | D |  |  |  | H | Menezes and Lucena 2014 | MZUSP- 6878; 6673 | Amaz/Neg |
| *Charax niger* Lucena, 1989 |  |  |  |  |  |  |  | H | Menezes and Lucena 2014 | MZUSP- 31137; 81137 | Amaz/Neg |
| *Charax notulatus* Lucena, 1987 |  |  |  | D |  |  |  |  | Ferreira et al. 2007 | INPA- 7884 | Neg/Ori |
| *Charax pauciradiatus* (Günther, 1864) |  |  |  |  |  |  |  | H | INPA Fish Collection | INPA- 9194; 29821 | Amaz |
| *Charax rupununi* Eigenmann, 1912 |  | B |  | D | E |  |  |  | Ferreira et al. 2007 | INPA- 14507 | Amaz/Neg/Tro |
| *Cheirodon micropterus*Eigenmann, 1907 |  |  |  |  |  |  |  | H | INPA Fish Collection | INPA- 53362 | Amaz/Neg |
| *Creagrutus* cf. *cochui* Géry, 1964 | A |  |  |  |  |  |  |  | Thomé-Souza and Chao 2004 | MZUSP- 28035 | Neg/upp Amaz |
| *Creagrutus ephippiatus* Vari & Harold, 2001 |  |  |  |  |  |  |  | H | Reis et al. 2003 | MBUCV- V29068 | Neg/Ori |
| *Creagrutus maxillari*s (Myers, 1927) |  |  |  |  |  |  |  | H | Reis et al. 2003 | INPA- 39457 | Neg/Ori/Gui |
| *Creagrutus melanzonus* Eigenmann, 1909 |  |  |  | D |  |  |  |  | Ferreira et al. 2007 | INPA- 16568 | Neg/Ori/Gui |
| *Creagrutus menezesi* Vari & Harold, 2001 |  |  |  | D |  |  |  | H | Reis et al. 2003 | MZUSP- 17714.0 | Neg/Tap |
| *Creagrutus phasma* Myers, 1927 |  |  |  | D |  | F |  |  | Reis et al. 2003 | MZUSP- 55753 | Neg/Ori |
| *Creagrutus runa* Vari & Harold, 2001 |  |  |  |  |  |  |  | H | Reis et al. 2003 | INPA- 29978; 43015 | Neg/Ori |
| *Creagrutus* sp. |  |  |  | D |  | F |  |  | Lima et al. 2005 | MZUSP- 66640 | Neg |
| *Creagrutus tuyuka* Vari & Lima, 2003 |  |  |  | D |  |  | G | H | Lima et al. 2005 | MZUSP- 81297 | Neg |
| *Creagrutus vexillapinnus* Vari & Harold, 2001 |  |  |  |  |  |  |  | H | Reis et al. 2003 | INPA- 39474; 42777 | Neg/Ori |
| *Creagrutus zephyrus* Vari & Harold, 2001 |  |  |  |  | E |  | G | H | Reis et al. 2003 | INPA- 36042; 36186 | Neg |
| *Ctenobrycon hauxwellianus* (Cope, 1870) |  | B |  | D | E | F |  |  | INPA Fish Collection | INPA- 28326; 28513 | Amaz/Neg |
| *Ctenobrycon spilurus* (Valenciennes, 1850) |  |  |  |  |  |  |  |  | INPA Fish Collection | INPA- 7871; 18614 | Amaz/Ori/Gui |
| *Cyanogaster noctivaga* Mattox; Britz; Toledo-Piza & Marinho, 2013 |  |  |  |  |  | F |  | H | Mattox et al. 2013 | INPA- 37904 | Neg |
| *Cynopotamus amazonus* (Günther, 1868) |  | B |  | D |  | F |  |  | Ferreira et al. 2007 | INPA- 11402; 12174 | Amaz/Neg |
| *Cynopotamus essequibensis* Eigenmann, 1912 |  |  |  |  |  |  |  | H | Dagosta and de Pinna 2019 | MZUSP- 10413 | Neg/Gui/low Amaz |
| *Exodon paradoxus* Müller & Troschel, 1844 |  |  |  | D |  | F |  |  | Ferreira et al. 2007 | INPA- 12255; 36848 | Amaz/Ori/Gui |
| *Gymnocorymbus thayeri* Eigenmann, 1908 |  |  |  |  | E |  |  |  | INPA Fish Collection | INPA- 28338; | Amaz/Ori/Gui |
| *Hemigrammus analis* Durbin, 1909 |  | B |  | D | E | F |  |  | Rapp Py-Daniel et al. 2017 | INPA- 21598; 38911 | Amaz/Ori/Gui |
| *Hemigrammus barrigonae* Eigenmann & Henn, 1914 |  |  |  |  |  |  | G | H | Ota et al. 2014 | MZUSP- 85018; 85106 | Neg/Ori/Gui |
| *Hemigrammus bellottii* (Steindachner, 1882) |  | B |  | D | E | F |  |  | Lima et al. 2005 | INPA- 29999; 36418 | Amaz/Ori/Gui |
| *Hemigrammus bleheri* Géry & Mahnert, 1986 |  |  |  | D | E |  |  |  | Rapp Py-Daniel et al. 2017 | INPA- 36416 | Neg/Ori |
| *Hemigrammus boesemani* Géry, 1959 |  |  |  |  |  |  |  | H | INPA Fish Collection | INPA- 30483 | Amaz/Gui |
| *Hemigrammus coeruleus* Durbin, 1908 |  |  |  | D | E |  |  |  | Lima et al. 2005 | INPA- 26255; 37831 | Amaz/Neg |
| *Hemigrammus cupreus* Durbin, 1918 |  |  |  |  |  |  |  | H | INPA Fish Collection | INPA- 51851 | Neg/mid Amaz |
| *Hemigrammus cylindricus* Durbin, 1909 |  |  |  | D |  |  |  |  | Goulding et al. 1988 | MZUSP- 31217 | Neg/Gui |
| *Hemigrammus geisleri* Zarske & Géry, 2007 |  |  |  |  |  |  |  | H | Zarske and Géry 2007 | INPA- 36855; 42779 | Amaz/Neg |
| *Hemigrammus gracilis* (Lütken, 1875) |  |  |  | D | E | F |  |  | Goulding et al. 1988 | INPA- 9757; 30442 | Amaz/Neg |
| *Hemigrammus guyanensis* Géry, 1959 |  |  |  | D |  |  |  |  | Goulding et al. 1988 | MZUSP- 35033; 35035 | Neg/Gui |
| *Hemigrammus hyanuary* Durbin, 1918 |  |  |  | D |  |  |  |  | Chao and Prada-Pedrero 1995 | INPA- 4399; 4687 | Amaz/Neg |
| *Hemigrammus iota* Durbin, 1909 |  |  |  | D |  |  |  |  | Kemenes and Forsberg 2014 | INPA- 29485; 39422 | Neg/Gui |
| *Hemigrammus kuroobi* Reia & Benine, 2019 |  |  |  |  |  |  |  | H | INPA Fish Collection | INPA- 57937 | Neg |
| *Hemigrammus levis* Durbin, 1908 |  | B |  | D | E | F |  |  | Rapp Py-Daniel et al. 2017 | INPA- 28339; 36049 | Amaz/Neg |
| *Hemigrammus lunatus* Durbin, 1918 |  |  |  |  |  |  |  | H | Ota et al. 2014 | INPA- 15339 | Amaz/Ori/Gui |
| *Hemigrammus marginatus* Ellis, 1911 |  |  |  |  |  |  |  |  | INPA Fish Collection | INPA- 42796 | Amaz/Ori/Gui/Par/SaF |
| *Hemigrammus melanochrous* Fowler, 1913 |  |  |  |  |  |  |  |  | INPA Fish Collection | INPA- 29911; 30438 | Amaz/Neg |
| *Hemigrammus micropterus* Meek, 1907 |  |  |  | D |  |  |  |  | INPA Fish Collection | INPA- 4788 | Neg/Ori |
| *Hemigrammus microstomus* Durbin, 1918 |  |  |  |  |  |  |  | H | INPA Fish Collection | INPA- 4788 | Amaz/Ori/Gui |
| *Hemigrammus mimu*s Böhlke, 1955 |  |  |  | D |  | F |  | H | Zarske and Géry 2007 | INPA- 42832 | Neg/Ori |
| *Hemigrammus newboldi* (Fernández-Yépez, 1949) |  |  |  |  |  |  |  |  | INPA Fish Collection | INPA- 39423 | Amaz/Ori |
| *Hemigrammus ocellifer* (Steindachner, 1882) |  | B |  | D | E |  |  |  | Kemenes and Forsberg 2014 | INPA- 13228; 14162 | Amaz/Gui |
| *Hemigrammus pretoensis* Géry, 1965 |  |  |  | D | E |  |  |  | Zuanon et al. 2015 | INPA- 14167; 14215 | Amaz/Neg |
| *Hemigrammus rodwayi* Durbin, 1909 |  |  |  |  |  |  |  |  | INPA Fish Collection | INPA- 38803; 39424 | Amaz/Gui |
| *Hemigrammus rubrostriatus*Zarske, 2015 |  |  |  |  |  |  |  | H | INPA Fish Collection | INPA-49559; 55779 | Neg/Ori |
| *Hemigrammus schmardae* (Steindachner, 1882) |  |  |  | D |  |  |  |  | Zuanon et al. 2008 | INPA- 29410; 32811 | Amaz/Ori |
| *Hemigrammus stictus* (Durbin, 1909) |  | B |  | D | E |  |  | H | Lima et al. 2009 | INPA- 26260; 34588 | Amaz/Ori |
| *Hemigrammus* sp. 'prata' |  |  |  |  |  |  |  | H | INPA Fish Collection | INPA- 36348; 36933 | Amaz/Neg |
| *Hemigrammus unilineatus* (Gill, 1858) |  |  |  | D |  |  |  |  | Ferreira et al. 2007 | MZUSP- 30323 | Amaz/Ori/Gui |
| *Hemigrammus vorderwinkleri* Géry, 1963 |  | B |  | D | E | F |  |  | Lima et al. 2005 | INPA- 15984; 32753 | Amaz/Ori |
| *Hemigrammus yinyang* Lima & Sousa, 2009 |  |  |  |  |  |  |  | H | INPA Fish Collection | INPA- 31879 | Amaz/Neg |
| *Hyphessobrycon* aff. *hasemani* Fowler, 1913 |  |  |  |  |  |  |  | H | Esguícero and Castro 2017 | MPEG- 575 | Amaz/Neg |
| *Hyphessobrycon* aff*. scutulatus*Lucena, 2003 |  |  |  |  |  |  |  | H | INPA Fish Collection | INPA- 51624 | Tap/Neg |
| *Hyphessobrycon* aff. *takasei* Géry, 1964 |  |  |  |  |  |  |  |  | INPA Fish Collection | INPA- 9220 | Amaz/Gui |
| *Hyphessobrycon agulha* Fowler, 1913 |  |  |  | D | E |  |  | H | Marinho 2010 | INPA- 19790; 29939 | Amaz/Neg |
| *Hyphessobrycon bentosi* Durbin, 1908 |  |  |  |  | E |  |  |  | Ferreira et al. 2007 | INPA- 37667; 36320 | Amaz/Neg |
| *Hyphessobrycon copelandi* Durbin, 1908 |  |  |  |  | E |  |  |  | Rapp Py-Daniel et al. 2017 | INPA- 34609; 37177 | Amaz/Neg |
| *Hyphessobrycon diancistrus* Weitzman, 1977 |  |  |  | D |  |  |  |  | Goulding et al. 1988 | INPA- 37108; 39275 | Neg/Ori |
| *Hyphessobrycon dorsalis* Zarske, 2014 |  |  |  |  |  |  |  | H | Zarske 2014 | MTD- F 33172–33183, | Neg/Ori |
| *Hyphessobrycon epicharis* Weitzman & Palmer, 1997 |  |  |  |  |  |  |  | H | Reis et al. 2003 | INPA- 9206; 37664 | Neg/Ori |
| *Hyphessobrycon eques* (Steindachner, 1882) |  |  |  |  |  |  |  | H | INPA Fish Collection | INPA- 16140 | Amaz/Par |
| *Hyphessobrycon erythrostigma* (Fowler, 1943) |  |  |  |  | E |  |  |  | Goulding et al. 1988 | MZUSP- 29866 | Neg/upp Amaz |
| *Hyphessobrycon heteresthes* (Ulrey, 1894) |  |  |  |  |  |  |  | H | INPA Fish Collection | INPA- 12686 | Amaz/Neg |
| *Hyphessobrycon heterorhabdus* (Ulrey, 1894) |  |  |  | D | E |  |  |  | Zuanon et al. 2008 | INPA- 13227; 14276 | Neg/upp Amaz |
| *Hyphessobrycon melanopterus* (Eigenmann, 1915) |  |  |  |  |  |  |  | H | UFAM Fish Collection | UFAM- 0289 | Amaz/Neg |
| *Hyphessobrycon* *melazonatus* Durbin, 1908 |  |  |  | D | E |  |  |  | Zuanon et al. 2015 | INPA- 14190; 25411 | Neg/Gui |
| *Hyphessobrycon paepkei* Zarske, 2014 |  |  |  |  |  |  |  | H | Zarske 2014 | MTDF- 33159;33166 | Neg |
| *Hyphessobrycon pyrrhonotus* Burgess, 1993 |  |  |  |  |  |  |  | H | Reis et al. 2003 | INPA- 32624; 35098 | Neg |
| *Hyphessobrycon rosaceus* Durbin, 1909 |  |  |  |  |  |  |  |  | INPA Fish Collection | INPA- 42652; 42742 | Neg/Gui |
| *Hyphessobrycon socolofi* Weitzman, 1977 |  |  |  | D |  |  |  |  | Goulding et al. 1988 | INPA- 32625; 32626 | Neg |
| *Hyphessobrycon sweglesi* (Géry, 1961) |  |  |  |  | E |  |  |  | Ferreira et al. 2007 | INPA- 34591; 37677 | Neg/Ori/upp Amaz |
| *Hyphessobrycon tropis* Géry, 1963 |  |  |  |  |  |  |  | H | Reis et al. 2003 | INPA- 32776 | Neg |
| *Hyphessobrycon tukunai*Géry, 1965 |  |  |  |  |  |  |  | H | INPA Fish Collection | INPA- 39379 | Amaz/Neg |
| *Jupiaba abramoides* (Eigenmann, 1909) |  |  |  | D |  |  |  |  | Lima et al. 2005 | INPA- 38935; 42370 | Neg/Ori/Gui |
| *Jupiaba* *acanthogaster* (Eigenmann, 1911) |  |  |  | D |  |  |  |  | Goulding et al. 1988 | MZUSP- 29907 | Neg/Tap/Par |
| *Jupiaba anteroides* (Géry, 1965) |  |  |  | D |  |  |  |  | Lima et al. 2005 | INPA- 9777; 42948 | Amaz/Ori |
| *Jupiaba atypindi* Zanata, 1997 |  |  |  | D |  |  |  | H | Reis et al. 2003 | INPA- 1306; 43020 | Neg (Bra) |
| *Jupiaba essequibensis* (Eigenmann, 1909) |  |  |  | D |  |  |  |  | INPA Fish Collection | INPA- 36107; 42781 | Neg/Gui |
| *Jupiaba pinnata* (Eigenmann, 1909) |  |  |  | D |  |  |  |  | Ferreira et al. 2007 | INPA- 12705; 12872 | Neg/Gui |
| *Jupiaba pirana* Zanata, 1997 |  |  |  | D |  |  |  |  | INPA Fish Collection | INPA- 39482 | Neg/Tap |
| *Jupiaba poekotero* Zanata & Lima, 2005 |  |  |  |  |  |  |  | H | Zanata and Lima 2005 | INPA- 43000; 43044 | Neg |
| *Jupiaba polylepis* (Günther, 1864) |  |  |  | D | E | F |  |  | Ferreira et al. 2007 | INPA- 21599; 36948 | Amaz/Ori/Gui |
| *Jupiaba scologaster* (Weitzman & Vari, 1986) | A |  |  | D | E | F |  |  | Goulding et al. 1988 | INPA- 36105; 43045 | Amaz/Ori |
| *Jupiaba* *zonata* (Eigenmann, 1908) |  | B |  | D |  |  |  |  | Reis et al. 2003 | INPA- 37750; 42754 | Amaz/Neg |
| *Knodus deuterodonoides* (Eigenmann, 1914) |  |  |  |  |  |  |  | H | INPA Fish Collection | INPA- 12873; 12874 | Neg/Ori |
| *Knodus* *heterestes* (Eigenmann, 1908) |  |  |  | D |  | F |  |  | Goulding et al. 1988 | MZUSP- 29899 | Neg/Ori/Gui |
| *Knodus orteguasae* (Fowler, 1943) |  |  |  |  |  |  |  | H | INPA Fish Collection | INPA- 36940 | Amaz/Neg |
| *Knodus smithi* (Fowler, 1913) |  |  |  |  |  |  |  | H | INPA Fish Collection | INPA- 37026 | Mad/Neg |
| *Knodus tiquiensis* Ferreira & Lima, 2006 |  |  |  |  |  | F |  | H | Ferreira and Lima 2006 | INPA- 24988; 37792 | Neg |
| *Leptobrycon jatuaranae* Eigenmann, 1915 |  |  |  | D |  |  |  |  | Goulding et al. 1988 | MZUSP- 29885 | Amaz/Neg |
| *Macropsobrycon* sp. |  |  |  |  |  |  |  | H | Goulding et al. 1988 | MZUSP- 29875 | Neg |
| *Microschemobrycon callops* Böhlke, 1953 | A |  |  | D |  | F |  |  | Lima et al. 2005 | INPA- 36161 | Amaz/Ori |
| *Microschemobrycon casiquiare* Böhlke, 1953 | A |  |  | D | E | F |  |  | Rapp Py-Daniel et al. 2017 | INPA- 11112; 36194 | Amaz/Ori |
| *Microschemobrycon* *geisleri* Géry, 1973 |  |  |  |  |  |  |  | H | INPA Fish Collection | INPA- 37805 | Amaz/Neg |
| *Microschemobrycon melanotus* (Eigenmann, 1912) | A |  |  | D | E | F |  |  | Rapp Py-Daniel et al. 2017 | INPA- 39359 | Neg/Tap/Mad/Amaz main channel/Gui |
| *Microschemobrycon meyburgi* Meinken, 1975 |  |  |  | D |  |  |  |  | Buckup et al. 2007 | MZUSP- 48131 | Neg |
| *Moenkhausia affinis* Steindachner, 1915 |  |  |  |  |  |  |  | H | Reis et al. 2003 | NMW- 003 | Neg |
| *Moenkhausia* aff. *browni* Eigenmann, 1909 |  |  |  | D |  |  |  |  | Ferreira et al. 2007 | INPA- 190781 | Neg/Ori |
| *Moenkhausia agnesae* Géry, 1965 |  |  |  |  |  |  |  | H | Zarske et al. 2004 | MTD- F 18310–18312 | Neg/upp Amaz |
| *Moenkhausia* *beninei* Lima & Soares, 2018 |  |  |  |  |  |  |  | H | Lima and Soares 2018 | ZUEC- 16838 | Neg |
| *Moenkhausia celibela* Marinho & Langeani, 2010 |  |  |  |  |  |  |  | H | INPA Fish Collection | INPA- 48592 | Tap/Xin/Mad/Neg/Amaz main channel |
| *Moenkhausia ceros* Eigenmann, 1908 |  |  |  | D |  |  |  |  | Goulding et al. 1988 | INPA- 13074; 30482 | Amaz/Ori |
| *Moenkhausia chrysargyrea* (Günther, 1864) |  | B |  | D | E | F |  |  | Ferreira et al. 2007 | INPA- 39447 | Amaz/Ori |
| *Moenkhausia collettii* (Steindachner, 1882) |  | B |  | D | E |  |  |  | Melo et al. 2011 | INPA- 15899; 28269 | Amaz/Ori/Gui |
| *Moenkhausia comma* Eigenmann, 1908 |  |  |  | D |  |  |  |  | Lima et al. 2005 | MZUSP- 76535 | Amaz/Neg |
| *Moenkhausia copei* (Steindachner, 1882) |  | B |  | D | E | F |  |  | Chao 2001 | INPA- 26615; 30432 | Amaz/Ori |
| *Moenkhausia cotinho* Eigenmann, 1908 |  | B |  | D |  | F |  |  | Melo et al. 2011 | INPA- 15780; 36401 | Amaz/Ori |
| *Moenkhausia dichroura* (Kner, 1858) |  |  |  | D |  |  |  |  | Ferreira et al. 2007 | INPA- 11110; 36845 | Amaz/Ori/Par |
| *Moenkhausia diktyota* Lima & Toledo-Piza, 2001 |  |  |  | D |  |  |  |  | Melo et al. 2011 | INPA- 16200; 29344 | Neg/Ori/Mad |
| *Moenkhausia gracilima* Eigenmann, 1908 |  |  |  |  |  | F |  | H | Marinho 2009 | INPA- 28784; 28786 | Amaz/Neg |
| *Moenkhausia grandisquamis* (Müller & Troschel, 1845) |  |  |  | D |  | F |  |  | Goulding et al. 1988 | INPA- 10781; 23025 | Amaz/Ori/Gui |
| *Moenkhausia hasemani* Eigenmann, 1917 |  |  |  |  |  |  |  | H | INPA Fish Collection | INPA- 16759 | Amaz/Gui |
| *Moenkhausia hemigrammoides* Géry, 1965 |  |  |  |  | E |  |  |  | Lima et al. 2005 | INPA- 9226; 39188 | Neg/Gui |
| *Moenkhausia icae* Eigenmann, 1908 |  |  |  |  |  |  |  | H | INPA Fish Collection | INPA- 42834 | Neg/upp Amaz |
| *Moenkhausia intermedia* Eigenmann, 1908 |  | B |  | D |  |  |  |  | Goulding et al. 1988 | INPA- 41081 | Amaz/Ori/Gui/Par |
| *Moenkhausia jamesi* Eigenmann, 1908 |  | B |  | D |  | F |  |  | Ferreira et al. 2007 | INPA- 36339 | Amaz/Neg |
| *Moenkhausia justae* Eigenmann, 1908 |  |  |  | D |  |  |  |  | Ferreira et al. 2007 | MZUSP- 55748 | Amaz/Ori |
| *Moenkhausia lata* Eigenmann, 1908 |  |  |  |  |  | F |  | H | Marinho 2009 | INPA- 36936; 42785 | Neg/Gui/Tap |
| *Moenkhausia lepidura* (Kner, 1858) |  | B |  | D |  | F |  |  | Melo et al. 2011 | INPA- 12717; 13244 | Amaz/Ori |
| *Moenkhausia megalops* (Eigenmann, 1907) |  |  |  | D |  |  |  |  | Ferreira et al. 2007 | MZUSP- 55645 | Amaz/Ori |
| *Moenkhausia miangi* Steindachner, 1915 |  |  |  | D |  |  |  |  | Reis et al. 2003 | NMW- 56297 | Neg/Ori |
| *Moenkhausia mikia* Marinho & Langeani, 2010 |  |  |  |  |  | F |  |  | Marinho and Langeani 2010a | INPA- 34346 | Amaz/Ori |
| *Moenkhausia oligolepis* (Günther, 1864) |  |  |  | D | E | F |  |  | Lima et al. 2005 | INPA- 30732; | Amaz/Ori/Gui |
| *Odontostilbe fugitiva* Cope, 1870 |  |  |  | D |  | F |  | H | Bührnheim and Malabarba 2006 | INPA- 18461; 18506 | Amaz/Neg |
| *Odontostilbe pulchra* (Gill, 1858) |  |  |  |  |  |  |  | H | Bührnheim and Malabarba 2007 | INPA- 36852 | Neg/Ori |
| *Odontostilbe* sp. 'macoari' |  |  |  | D |  |  |  |  | Ferreira et al. 2007 | INPA- 36951 | Neg (Bra) |
| *Oxybrycon parvulus* Géry, 1964 |  |  |  |  |  |  |  | H | INPA Fish Collection | INPA- 42809 | Neg/upp Amaz |
| *Paracheirodon axelrodi* (Schultz, 1956) |  |  |  | D | E |  |  |  | Rapp Py-Daniel et al. 2017 | INPA- 12506; 12510 | Neg/Ori |
| *Paracheirodon simulans* (Géry, 1963) |  |  |  | D | E |  |  | H | INPA Fish Collection | INPA- 14399; 28289 | Neg/Ori |
| *Parapristella aubynei* (Eigenmann, 1909) |  |  |  |  |  |  |  |  | INPA Fish Collection | INPA -52127; 57823 | Neg/Gui |
| *Parapristella georgiae* Géry, 1964 |  |  |  |  | E |  |  | H | INPA Fish Collection | INPA- 4758; 36389 | Neg/Ori |
| *Parecbasis cyclolepis* Eigenmann, 1914 |  |  |  |  |  |  |  | H | Esguícero and Castro 2017 | LIRP- 5007 | Amaz/Neg |
| *Petitella georgiae* Géry & Boutière, 1964 |  |  |  | D |  |  |  |  | Chao and Prada-Pedrero 1995 | INPA- 34583 | Neg/upp Amaz |
| *Petitella* sp. |  |  |  |  |  |  |  | H | Ferreira et al. 2007 | MZUSP- 17663 | Neg (Bra) |
| *Phenacogaster pectinata* (Cope, 1870) |  |  |  | D | E |  |  |  | Ferreira et al. 2007 | INPA- 37165; 42887 | Amaz/Neg |
| *Phenacogaster* *prolata* Lucena & Malabarba, 2010 |  |  |  |  | E |  |  | H | Lucena and Malabarba 2010 | MZUSP- 31207 | Neg/Ori |
| *Phenacogaster retropinnus* Lucena & Malabarba, 2010 |  |  |  |  |  |  |  | H | Lucena and Malabarba 2010 | INPA- 42837 | Amaz/Neg |
| *Phenacogaster megalostictus* Eigenmann, 1909 |  |  |  |  |  |  |  | H | Lucena and Malabarba 2010 | INPA- 37021 | Neg/Gui |
| *Phenacogaster microstictus* Eigenmann, 1909 |  |  |  | D | E |  |  |  | Ferreira et al. 2007 | INPA- 9176 | Neg/Gui |
| *Poptella brevispina* Reis, 1989 |  |  |  | D |  |  |  |  | Ferreira et al. 2007 | MZUSP- 73481 | Neg/Ori/Gui |
| *Poptella compressa* (Günther, 1864) |  | B |  | D | E |  |  |  | Ferreira et al. 2007 | INPA- 36937; 36957 | Amaz/Ori/Gui |
| *Poptella longipinnis* (Popta, 1901) |  |  |  | D |  |  |  |  | Ferreira et al. 2007 | INPA- 7892 | Neg/Ori/Gui/Toc |
| *Priocharax ariel* Weitzman & Vari, 1987 |  |  |  |  |  |  |  | H | Reis et al. 2003 | INPA- 25243 | Neg/Ori |
| *Priocharax nanus* Toledo-Piza, Mattox & Britz, 2014 |  |  |  |  |  |  |  | H | Toledo-Piza et al. 2014 | INPA- 39891 | Neg |
| *Priocharax pygmaeus* Weitzman & Vari, 1987 |  |  |  |  |  |  |  | H | INPA Fish Collection | INPA- 42851 | Neg/upp Amaz |
| *Prionobrama filigera* (Cope, 1870) |  |  |  |  |  |  |  | H | INPA Fish Collection | INPA- 9096; 33377 | Amaz/Neg |
| *Ptychocharax rhyacophila* Weitzman, Fink, Machado-Allison & Royero, 1994 |  |  |  |  |  |  |  | H | Weitzman et al. 1994 | MBUCV-V- 20400 | Neg |
| *Rhinobrycon negrensis* Myers, 1944 |  |  |  | D |  | F |  | H | Goulding et al. 1988 | INPA- 16745 | Neg/Ori/Amaz |
| *Roeboides affinis* (Günther, 1868) |  | B |  | D |  | F |  | H | Lucena 2007 | INPA- 16765; 26601 | Amaz/Neg |
| *Roeboides biserialis* (Garman, 1890) | A |  |  |  |  |  |  |  | Lucena 2007 | MZUSP- 88001 | Amaz/Neg |
| *Roeboides myersii* Gill, 1870 |  |  |  |  |  |  |  | H | Reis et al. 2003 | INPA- 25810 | Amaz/Ori |
| *Roeboides oligistos* Lucena, 2000 |  |  |  | D |  |  |  | H | Lucena 2007 | MZUSP- 20183; 34711 | Amaz/Neg |
| *Schultzites* *axelrodi* Géry, 1964 |  |  |  | D |  |  |  |  | Ferreira et al. 2007 | MZUSP- 17697; 17706 | Neg/Ori |
| *Serrabrycon magoi* Vari, 1986 |  |  |  | D | E |  |  | H | Goulding et al. 1988 | MZUSP- 28749 | Neg/Ori |
| *Tetragonopterus argenteus* Cuvier, 1816 |  | B |  | D | E | F |  |  | Melo et al. 2011 | INPA- 12275; 14749 | Amaz/Ori/Par |
| *Tetragonopterus chalceus* Spix & Agassiz, 1829 | A | B |  | D |  | F |  |  | Melo et al. 2011 | INPA- 14791; 30663 | Amaz/Ori/Gui |
| *Tetragonopterus manaos* Urbanski; Melo; Silva & Benine, 2018 |  |  |  |  |  |  |  | H | Urbanski et al. 2018 | INPA- 25533; 44352 | Amaz/Neg |
| *Thayeria obliqua* Eigenmann, 1908 |  |  |  | D |  |  |  |  | Goulding et al. 1988 | INPA- 42840 | Amaz/Ori |
| *Thrissobrycon pectinifer* Böhlke, 1953 |  |  |  | D |  |  |  |  | Goulding et al. 1988 | INPA- 17503; 38342 | Neg/Ori |
| *Tucanoichthys tucano* Géry & Römer, 1997 |  |  |  | D |  |  |  | H | Marinho 2010 | MZUSP- 51321 | Neg |
| *Tyttobrycon spinosus* Géry, 1973 |  |  |  |  |  |  |  | H | Esguícero and Castro 2017 | INPA- 36395 | Mad/Neg |
| *Tyttobrycon xeruini* Géry, 1973 |  |  |  | D |  |  |  | H | Marinho et al. 2013 | INPA- 54870; 54972 | Neg/Ori |
| **Chilodontidae** |  |  |  |  |  |  |  |  |  |  |  |
| *Caenotropus labyrinthicus* (Kner, 1858) | A | B |  | D | E | F |  |  | Goulding et al. 1988 | INPA- 26616; 42820 | Amaz/Ori/Gui |
| *Caenotropus mestomorgmatos* Vari, Castro & Raredon, 1995 |  |  |  |  |  |  |  | H | Reis et al. 2003 | MZUSP- 29359 | Amaz/Ori |
| *Chilodus gracilis* Isbrücker & Nijssen, 1988 |  |  |  |  |  |  |  | H | Reis et al. 2003 | INPA- 11222 | Amaz/Neg |
| *Chilodus punctatus* Müller & Troschel, 1844 |  | B |  |  | E |  |  |  | Lima et al. 2005 | INPA- 11227; 30671 | Amaz/Ori/Gui |
| **Crenuchidae** |  |  |  |  |  |  |  |  |  |  |  |
| *Ammocryptocharax elegans* Weitzman & Kanazawa, 1976 |  |  |  | D | E |  |  |  | Lima et al. 2005 | INPA- 14175 | Amaz/Ori |
| *Ammocryptocharax minutus* Buckup, 1993 |  |  |  |  | E |  |  | H | Reis et al. 2003 | MZUSP- 43671 | Neg/Ori |
| *Characidium boaevistae* Steindachner, 1915 |  |  |  | D |  |  |  |  | Buckup et al. 2007 | NMW- 62431 | Neg/Ori |
| *Characidium crandellii* Steindachner, 1915 |  |  |  | D |  |  |  |  | Ferreira et al. 2007 | INPA- 1635; 14131 | Neg/Gui |
| *Characidium* *declivirostre* Steindachner, 1915 |  |  |  | D |  |  | G |  | Lima et al. 2005 | INPA- 14130; 15841 | Amaz/Ori |
| *Characidium fasciatum* Reinhardt, 1867 |  |  |  |  |  |  |  | H | INPA Fish Collection | INPA- 38891 | Neg/Ori |
| *Characidium hasemani* Steindachner, 1915 |  |  |  | D | E |  |  |  | Ferreira et al. 2007 | INPA- 1308; 1747 | Neg/Ori |
| *Characidium longum* Taphorn, Montaña & Buckup, 2006 |  |  |  |  |  |  |  | H | INPA Fish Collection | INPA- 39144; 39475 | Neg/Ori |
| *Characidium pellucidum* Eigenmann, 1909 |  |  |  | D | E |  |  |  | Ferreira et al. 2007 | INPA- 36884 | Neg/Ori/Gui |
| *Characidium pteroides* Eigenmann, 1909 |  |  |  | D | E | F | G |  | Lima et al. 2005 | INPA- 1744; 14128 | Amaz/Ori/Gui |
| *Characidium steindachneri* Cope, 1878 |  |  |  | D |  |  |  |  | Ferreira et al. 2007 | INPA- 8153; 36886 | Amaz/Ori/Gui |
| *Characidium zebra* Eigenmann, 1909 |  |  |  | D | E |  | G |  | Ferreira et al. 2007 | INPA- 11699; 14140 | Amaz/Ori/Gui |
| *Crenuchus spilurus* Günther, 1863 |  |  |  |  | E |  |  |  | Zuanon et al. 2015 | INPA- 28246 | Amaz/Ori/Gui |
| *Elachocharax geryi* Weitzman & Kanazawa, 1978 |  |  |  |  |  |  |  | H | Reis et al. 2003 | MZUSP- 13249 | Neg/Ori |
| *Elachocharax junki* (Géry, 1971) |  |  |  |  | E |  |  |  | Ferreira et al. 2007 | INPA- 14125; 36898 | Neg/mid Amaz |
| *Elachocharax mitopterus* Weitzman, 1986 |  |  |  |  | E |  |  |  | Ferreira et al. 2007 | INPA- 14116; 38818 | Neg/Ori |
| *Elachocharax pulcher* Myers, 1927 |  |  |  |  | E |  |  |  | Zuanon et al. 2008 | INPA- 14119; 36092 | Amaz/Ori |
| *Leptocharacidium omospilus* Buckup, 1993 |  |  |  |  |  |  |  | H | Reis et al. 2003 | INPA- 14129; 30345 | Neg/Ori |
| *Melanocharacidium blennioides* (Eigenmann, 1909) |  |  |  |  |  |  |  | H | INPA Fish Collection | INPA- 9209 | Neg/Ori/Gui |
| *Melanocharacidium depressum* Buckup, 1993 |  |  |  |  | E |  | G | H | Reis et al. 2003 | INPA- 37755; 43047 | Neg/Ori |
| *Melanocharacidium dispilomma* Buckup, 1993 |  |  |  |  | E |  | G | H | Ferreira et al. 2007 | INPA- 1694; 11700 | Amaz/Ori/Gui |
| *Melanocharacidium melanopteron* Buckup, 1993 |  |  |  |  |  |  |  | H | INPA Fish Collection | INPA- 37787 | Neg/Ori |
| *Melanocharacidium nigrum* Buckup, 1993 |  |  |  | D |  |  |  |  | Ferreira et al. 2007 | INPA- 1681; 11658 | Neg/upp Amaz |
| *Melanocharacidium pectorale* Buckup, 1993 |  |  |  | D |  |  |  | H | Reis et al. 2003 | INPA- 14146 | Amaz/Ori |
| *Microcharacidium eleotrioides* (Géry, 1960) |  |  |  | D | E |  |  |  | Zuanon et al. 2015 | INPA- 25242; 27739 | Amaz/Gui |
| *Microcharacidium gnomus* Buckup, 1993 |  |  |  | D |  |  |  |  | Rapp Py-Daniel et al. 2017 | USNM- 270168 | Neg/Ori/Amaz |
| *Microcharacidium weitzmani* Buckup, 1993 |  |  |  | D | E |  |  |  | Zuanon et al. 2008 | INPA- 14120; 37028 | Amaz/Ori |
| *Odontocharacidium aphane*s (Weitzman & Kanazawa, 1977) |  |  |  |  | E |  |  |  | Rapp Py-Daniel et al. 2017 | INPA- 29360; 32772 | Amaz/Ori |
| *Poecilocharax weitzmani* Géry, 1965 |  |  |  | D | E |  |  |  | Lima et al. 2005 | INPA- 27715; 29345 | Neg/Ori/upp Amaz |
| **Curimatidae** |  |  |  |  |  |  |  |  |  |  |  |
| *Curimata cisandina* (Allen, 1942) |  |  |  | D |  |  |  |  | Ferreira et al. 2007 | MZUSP- 31726 | Neg/upp Amaz |
| *Curimata cyprinoides* (Linnaeus, 1766) |  | B |  | D |  |  |  |  | Ferreira et al. 2007 | INPA- 2663; | Amaz/Ori/Gui |
| *Curimata incompta* Vari, 1984 | A |  |  |  |  |  |  |  | Melo and Vari 2014 | INPA 3718 | Amaz/Ori |
| *Curimata inornata* Vari, 1989 |  | B | C | D |  |  |  |  | Goulding et al. 1988 | INPA- 25535 | Amaz/Neg |
| *Curimata knerii* Steindachner, 1876 |  | B |  | D |  | F |  |  | Goulding et al. 1988 | INPA- 5751 | Amaz/Neg |
| *Curimata ocellata* Eigenmann & Eigenmann, 1889 |  | B |  | D |  |  |  |  | Ferreira et al. 2007 | INPA- 35678 | Amaz/Ori |
| *Curimata roseni* Vari, 1989 |  |  |  | D |  |  |  |  | Ferreira et al. 2007 | INPA- 35759 | Amaz/Ori/Gui |
| *Curimata vittata* (Kner, 1858) | A | B | C | D |  | F |  |  | Ferreira et al. 2007 | INPA- 12563; 12661 | Amaz/Ori/Gui |
| *Curimatella alburna* (Müller & Troschel, 1844) |  |  |  | D |  | F |  |  | Lima et al. 2005 | INPA- 12213 | Amaz/Gui |
| *Curimatella dorsalis* (Eigenmann & Eigenmann, 1889) |  |  |  |  |  |  |  |  | INPA Fish Collection | INPA- 11219 | Amaz/Ori/Par |
| *Curimatella immaculata* (Fernández-Yépez, 1948) |  | B |  | D |  |  |  |  | Ferreira et al. 2007 | MZUSP- 21171 | Amaz/Ori/Gui |
| *Curimatella meyeri* (Steindachner, 1882) | A | B | C | D |  |  |  |  | Zuanon et al. 1998 | INPA- 9793; 28927 | Amaz/Neg |
| *Curimatopsis cryptica* Vari, 1982 |  |  |  | D |  |  |  |  | Goulding et al. 1988 | INPA- 12360; 39406 | Amaz/Ori/Gui |
| *Curimatopsis evelynae* Géry, 1964 |  | B |  | D | E |  |  |  | Rapp Py-Daniel et al. 2017 | INPA- 1669; 35314 | Neg/Ori |
| *Curimatopsis microlepis* Eigenmann & Eigenmann, 1889 |  |  |  |  |  |  |  |  | Goulding et al. 1988 | INPA- 4790; 4791 | Amaz/Neg |
| *Curimatopsis macrolepis* (Steindachner, 1876) |  | B |  | D | E |  |  |  | Ferreira et al. 2007 | INPA- 30666; 33215 | Amaz/Ori |
| *Curimatopsis pallida* Melo & Oliveira, 2017 |  |  |  |  |  |  |  | H | Melo and Oliveira 2017 | MZUSP- 121192 | Neg |
| *Cyphocharax abramoides* (Kner, 1859) |  | B |  | D |  | F |  |  | Ferreira et al. 2007 | INPA- 15924; 30710 | Amaz/Ori |
| *Cyphocharax* *festivus* Vari, 1992 |  |  |  | D |  |  |  |  | Ferreira et al. 2007 | INPA- 36846; 36874 | Amaz/Ori/Gui |
| *Cyphocharax leucostictus* (Eigenmann & Eigenmann, 1889) |  |  |  | D |  | F |  |  | Ferreira et al. 2007 | INPA- 31374 | Amaz/Ori |
| *Cyphocharax mestomyllon* Vari, 1992 |  |  |  |  |  |  |  | H | Reis et al. 2003 | MZUSP- 41755; 41756 | Neg |
| *Cyphocharax multilineatus* (Myers, 1927) |  |  |  | D |  |  |  |  | Melo and Vari 2014 | INPA- 9235 | Amaz/Ori |
| *Cyphocharax nigripinnis* Vari, 1992 |  |  |  | D |  |  |  |  | Ferreira et al. 2007 | INPA- 25579 | Amaz/Neg |
| *Cyphocharax notatus* (Staindachner, 1908) |  | B |  |  |  |  |  |  | UFAM Fish Collection | UFAM- 0144 | Amaz/Neg |
| *Cyphocharax plumbeus* (Eigenmann & Eigenmann, 1889) |  | B |  | D |  | F |  |  | Goulding et al. 1988 | INPA- 26849 | Amaz/Neg |
| *Cyphocharax sanctigabrielis* Melo & Vari, 2014 |  |  |  |  |  |  |  | H | Melo and Vari 2014 | MZUSP- 115004 | Neg |
| *Cyphocharax spilurus* (Günther, 1864) |  | B |  | D | E | F |  |  | Lima et al. 2005 | INPA- 9210; 9211 | Amaz/Ori/Gui |
| *Potamorhina altamazonica* (Cope, 1878) |  | B |  |  |  |  |  |  | Saint-Paul et al. 2000 | INPA- 26597 | Amaz/Ori |
| *Potamorhina latior* (Spix & Agassiz, 1829) |  | B |  | D |  | F |  |  | Goulding et al. 1988 | INPA- 25811; 35987 | Amaz/Neg |
| *Potamorhina pristigaster* (Steindachner, 1876) |  |  |  | D |  |  |  |  | Zuanon et al. 2008 | INPA- 9800; 35555 | Amaz/Neg |
| *Psectrogaster amazonica* Eigenmann & Eigenmann, 1889 |  |  |  |  |  |  |  |  | INPA Fish Collection | INPA- 6080 | Amaz/Neg |
| *Psectrogaster ciliata* (Müller & Troschel, 1844) |  |  |  | D |  |  |  |  | Ferreira et al. 2007 | INPA- 43074 | Amaz/Neg |
| *Psectrogaster essequibensis* (Günther, 1864) |  |  |  |  |  |  |  | H | INPA Fish Collection | INPA- 35711; 35990 | Amaz/Gui |
| *Psectrogaster rutiloides* (Kner, 1858) |  | B |  | D |  |  |  |  | Saint-Paul et al. 2000 | MZUSP- 19290 | Amaz/Neg |
| *Steindachnerina bimaculata* (Steindachner, 1876) |  |  |  |  |  |  |  | H | INPA Fish Collection | INPA- 55118 | Amaz/Ori |
| *Steindachnerina hypostoma* (Boulenger, 1887) |  |  |  | D |  |  |  |  | Ferreira et al. 2007 | MZUSP- 29536 | Amaz/Neg |
| *Steindachnerina leucisca* (Günther, 1868) |  |  |  |  |  |  |  | H | Buckup et al. 2007 | MZUSP- 19289; 75474 | Neg/mid Amaz |
| *Steindachnerina planiventris* Vari & Vari, 1989 |  |  |  | D |  |  |  | H | INPA Fish Collection | INPA- 36171 | Amaz/Neg |
| **Ctenoluciidae** |  |  |  |  |  |  |  |  |  |  |  |
| *Boulengerella cuvieri* (Agassiz, 1829) |  | B |  | D |  | F |  | H | INPA Fish Collection | INPA- 16743; 36925 | Amaz/Ori/Gui |
| *Boulengerella lateristriga* (Boulenger, 1895) |  | B |  | D |  |  |  |  | INPA Fish Collection | INPA- 1201; 35559 | Amaz/Ori |
| *Boulengerella lucius* (Cuvier, 1816) |  | B |  | D |  | F |  |  | INPA Fish Collection | INPA- 31388; 35660 | Amaz/Ori |
| *Boulengerella maculata* (Valenciennes, 1850) |  | B |  | D |  | F |  |  | INPA Fish Collection | INPA- 3175; 11654; | Amaz/Ori |
| *Boulengerella xyrekes* Vari, 1995 |  |  |  | D |  | F |  | H | INPA Fish Collection | INPA- 42817 | Amaz/Ori |
| **Cynodontidae** |  |  |  |  |  |  |  |  |  |  |  |
| *Cynodon gibbus* (Spix & Agassiz, 1829) | A | B | C | D |  | F |  |  | Zuanon et al. 2008 | INPA- 31373; 35546 | Amaz/Ori/Gui |
| *Cynodon septenarius* Toledo-Piza, 2000 |  |  |  | D |  |  |  |  | Lima et al. 2005 | INPA- 26404 | Amaz/Ori/Gui |
| *Hydrolycus armatus* (Jardine, 1841) |  |  |  | D |  |  |  |  | Ferreira et al. 2007 | INPA- 5028; 35946 | Amaz/Ori/Gui |
| *Hydrolycus scomberoides* (Cuvier, 1819) | A | B |  | D |  | F |  |  | Goulding et al. 1988 | INPA- 3159; 25824 | Amaz/Neg |
| *Hydrolycus tatauaia* Toledo-Piza, Menezes & Santos, 1999 |  |  |  | D |  |  |  |  | Lima et al. 2005 | INPA- 26416; 35657 | Neg/Ori/Gui |
| *Hydrolycus wallacei* Toledo-Piza, Menezes & Santos, 1999 |  | B |  | D |  |  |  |  | Zuanon et al. 2008 | INPA- 26417; 36656 | Amaz/Ori |
| *Raphiodon vulpinus* Spix & Agassiz, 1829 |  | B |  | D |  | F |  |  | Zuanon et al. 1998 | INPA- 7754; 13345 | Amaz/Ori/Gui/Par |
| **Erythrinidae** |  |  |  |  |  |  |  |  |  |  |  |
| *Erythrinus erythrinus* (Bloch & Schneider, 1801) |  |  |  | D | E |  |  |  | Lima et al. 2005 | INPA- 11301; 14288 | Amaz/Ori/Gui/Par |
| *Hoplerythrinus unitaeniatus* (Spix & Agassiz, 1829) |  | B |  | D | E |  |  |  | Lima et al. 2005 | INPA- 12947; 14298 | Amaz/Ori/Gui/Par/SaF |
| *Hoplias aimara* (Valenciennes, 1847) |  |  |  | D |  |  |  |  | Ferreira et al. 2007 | INPA- 11303 | Amaz/Ori/Gui/Par |
| *Hoplias curupira* Oyakawa & Mattox, 2009 |  |  |  |  | E |  |  | H | Oyakawa and Mattox 2009 | INPA- 38842 | Amaz/Ori/Gui |
| *Hoplias malabaricus* (Bloch, 1794) |  | B |  | D | E | F |  |  | Lima et al. 2005 | INPA- 10319; 12053 | Amaz/Ori/Gui/Par |
| **Gasteropelecidae** |  |  |  |  |  |  |  |  |  |  |  |
| *Carnegiella marthae* Myers, 1927 |  | B |  | D | E |  |  |  | Rapp Py-Daniel et al. 2017 | INPA- 32681; 32688 | Amaz/Ori |
| *Carnegiella strigata* (Günther, 1864) |  | B |  | D | E |  |  |  | Zuanon et al. 2015 | INPA- 29297; 29301 | Amaz/Ori/Gui |
| *Thoracocharax stellatus* (Kner, 1858) |  |  |  |  |  |  |  | H | INPA Fish Collection | INPA- 16770; 26539 | Amaz/Ori/Par |
| **Hemiodontidae** |  |  |  |  |  |  |  |  |  |  |  |
| *Anodus elongatus* Agassiz, 1829 |  | B |  | D |  | F |  |  | Zuanon et al. 1998 | INPA- 25815 | Amaz/Neg |
| *Anodus orinocensis* (Steindachner, 1887 |  | B |  | D |  |  |  |  | INPA Fish Collection | INPA- 25794 | Amaz/Ori |
| *Argonectes longiceps* (Kner, 1858) |  | B |  | D |  | F |  |  | Zuanon et al. 1998 | INPA- 12101; 26614 | Amaz/Gui |
| *Bivibranchia fowleri* (Steindachner, 1908) |  |  |  | D |  |  |  |  | Lima et al. 2005 | INPA- 35756; 36102 | Amaz/Ori/Gui |
| *Bivibranchia* sp. |  |  |  | D |  |  |  |  | Ferreira et al. 2007 | MZUSP- 29628 | Neg (Bra) |
| *Bivibranchia velox*(Eigenmann & Myers, 1927) |  |  |  |  |  |  |  | H | INPA Fish Collection | INPA-51936 | Toc/Xin/Neg |
| *Hemiodus argenteus* Pellegrin, 1909 |  | B |  | D |  | F |  | H | Ferreira et al. 2007 | INPA- 9797; 25820 | Amaz/Ori/Gui |
| *Hemiodus atranalis* (Fowler, 1940) |  | B |  | D |  |  |  |  | Zuanon et al. 2008 | INPA- 30477; 35581 | Amaz/Gui |
| *Hemiodus goeldii* Steindachner, 1908 |  | B |  | D |  | F |  |  | Zuanon et al. 2008 | INPA- 11218; 39036 | Neg/low Amaz |
| *Hemiodus gracilis* Günther, 1864 |  | B |  | D |  | F |  |  | INPA Fish Collection | INPA- 26618; 36631 | Amaz/Ori |
| *Hemiodus immaculatus* Kner, 1858 |  | B |  | D |  | F |  | H | INPA Fish Collection | INPA- 12075; 26419 | Amaz/Ori |
| *Hemiodus microlepis* Kner, 1858 |  | B |  | D |  | F |  |  | Ferreira et al. 2007 | INPA- 5473 | Amaz/Ori |
| *Hemiodus quadrimaculatus* Pellegrin, 1909 |  | B |  | D |  |  |  | H | Reis et al. 2003 | INPA- 1300 | Amaz/Gui |
| *Hemiodus semitaeniatus* Kner, 1858 |  | B |  | D |  | F |  |  | Lima et al. 2005 | INPA- 42997; 43034 | Amaz/Ori/Gui/Par |
| *Hemiodus* sp. 'rabo de fogo' |  | B |  | D |  | F |  |  | Langeani 1996 | INPA- 25615; 25767 | Amaz/Neg |
| *Hemiodus thayeria* Böhlke, 1955 |  | B |  | D |  | F |  |  | INPA Fish Collection | INPA- 6549; 12188 | Amaz/Ori |
| *Hemiodus unimaculatus* (Bloch, 1794) |  | B | C | D |  | F |  |  | INPA Fish Collection | INPA- 11223; 26420 | Amaz/Gui |
| *Micromischodus sugillatus* Roberts, 1971 |  | B |  | D |  | F |  |  | Zuanon et al. 1998 | INPA- 26604; 35579 | Amaz/Neg |
| **Iguanodectidae** |  |  |  |  |  |  |  |  |  |  |  |
| *Bryconops affinis* (Günther, 1864) |  |  |  |  |  |  |  | H | Wingert and Malabarba 2011 | INPA- 39328 | Amaz/Ori/Gui |
| *Bryconops* *caudomaculatus* (Günther, 1864) |  | B |  | D | E | F |  |  | Ferreira et al. 2007 | INPA- 12399; 14301 | Amaz/Ori/Gui |
| *Bryconops alburnoides* Kner, 1858 |  | B |  | D |  | F |  |  | Goulding et al. 1988 | INPA- 30701; 35675 | Amaz/Ori |
| *Bryconops collettei*Chernoff & Machado-Allison, 2005 |  |  |  |  |  |  |  | H | INPA Fish Collection | INPA- 42775; 42975 | Amaz/Neg |
| *Bryconops disruptus* Machado-Allison & Chernoff, 1997 |  |  |  |  |  |  |  | H | Reis et al. 2003 | INPA- 41396 | Neg |
| *Bryconops giacopinii* (Fernández-Yépez, 1950) |  | B |  | D | E | F |  |  | Ferreira et al. 2007 | INPA- 14300; 16569 | Amaz/Ori |
| *Bryconops gracilis* (Eigenmann, 1908) |  | B |  | D |  |  |  |  | Zuanon et al. 1998 | INPA- 3316 | Neg/Tap |
| *Bryconops humeralis* Machado-Allison, Chernoff & Buckup, 1996 |  |  |  | D | E |  |  |  | Lima et al. 2005 | INPA- 42911 | Neg/Ori |
| *Bryconops inpai* Knöppel, Junk & Géry, 1968 |  |  |  | D | E |  |  |  | Ferreira et al. 2007 | INPA- 27729; 28619 | Amaz/Ori |
| *Bryconops magoi*Chernoff & Machado-Allison, 2005 |  |  |  |  |  |  |  | H | INPA Fish Collection | INPA-49393; 49403 | Amaz/Ori |
| *Bryconops melanurus* (Bloch, 1794) |  |  |  | D |  | F |  |  | Goulding et al. 1988 | INPA- 15776; 15854 | Amaz/Gui/Par |
| *Bryconops* *vibex* Machado-Allison; Chernoff & Buckup, 1996 |  |  |  |  |  |  |  | H | Silva-Oliveira et al. 2015 | INPA- 19638; 19642 | Neg/Ori |
| *Iguanodectes adujai* Géry, 1970 |  |  |  | D |  | F |  |  | Goulding et al. 1988 | INPA- 4754; 9765 | Amaz/Ori |
| *Iguanodectes geisleri* Géry, 1970 |  | B |  | D | E |  |  |  | Zuanon et al. 2015 | INPA- 15749; 27757 | Amaz/Ori |
| *Iguanodectes gracilis* Géry, 1993 |  |  |  | D |  |  |  |  | Zuanon et al. 2008 | INPA- 29909 | Neg |
| *Iguanodectes spilurus* (Günther, 1864) |  |  |  | D | E | F |  |  | Ferreira et al. 2007 | INPA- 14071; 30456 | Amaz/Ori/Gui |
| *Iguanodectes variatus* Géry, 1993 |  |  |  | D | E |  |  |  | Ferreira et al. 2007 | INPA- 13231; 14185 | Neg/mid Amaz |
| **Lebiasinidae** |  |  |  |  |  |  |  |  |  |  |  |
| *Copeina guttata* (Steindachner, 1876) |  |  |  |  |  |  |  | H | Reis et al. 2003 | NMW- 56966; 56967 | Neg/upp Amaz |
| *Copella compta* (Myers, 1927) |  |  |  | D |  |  |  |  | Marinho and Menezes 2017 | INPA- 4694; 42926 | Neg |
| *Copella callolepis* (Regan, 1912) |  | B |  |  | E |  |  | H | Marinho and Menezes 2017 | INPA- 14223; 28241 | Amaz/Neg |
| *Copella eigenmanni* (Regan, 1912) |  | B |  |  | E |  |  | H | Marinho and Menezes 2017 | INPA- 37783; 38956 | Neg/Ori |
| *Copella nattereri* (Steindachner, 1876) |  | B |  | D | E |  |  |  | Marinho and Menezes 2017 | INPA- 2191; 13255 | Neg/Ori/Gui |
| *Lebiasina yepezi* Netto-Ferreira, Oyakawa, Zuanon & Nolasco, 2011 |  |  |  | D |  |  |  | H | Netto-Ferreira et al. 2011 | INPA- 15091; 15742 | Neg/Ori |
| *Nannostomus anduzei* Fernandez & Weitzman, 1987 |  |  |  |  |  |  |  | H | INPA Fish Collection | INPA- 4697; 4677 | Neg/Ori |
| *Nannostomus beckfordi* Günther, 1872 |  |  |  |  | E |  |  |  | INPA Fish Collection | INPA- 28287; 39319 | Neg/Gui/upp Amaz |
| *Nannostomus digrammus* (Fowler, 1913) |  | B |  | D | E |  |  |  | Lima et al. 2005 | INPA- 26612; 37062 | Amaz/Gui |
| *Nannostomus eques* Steindachner, 1876 |  | B |  | D | E |  |  |  | Rapp Py-Daniel et al. 2017 | INPA- 15890; 29417 | Amaz/Ori |
| *Nannostomus marginatus* Eigenmann, 1909 |  |  |  | D | E |  |  |  | Lima et al. 2005 | INPA- 14170; 25392 | Amaz/Ori/Gui |
| *Nannostomus marilynae* Weitzman & Cobb, 1975 |  |  |  | D | E | F |  |  | Rapp Py-Daniel et al. 2017 | INPA- 4680; 11613 | Neg/Ori |
| *Nannostomus trifasciatus* Steindachner, 1876 |  | B |  | D | E |  |  |  | Goulding et al. 1988 | INPA- 11829; 26273 | Amaz/Ori |
| *Nannostomus unifasciatus* Steindachner, 1876 |  | B |  | D | E |  |  |  | Rapp Py-Daniel et al. 2017 | INPA- 11636; 11822 | Amaz/Ori/Gui |
| *Pyrrhulina brevis* Steindachner, 1876 |  |  |  | D | E |  |  |  | Zuanon et al. 2008 | INPA- 13229; 14165 | Amaz/Ori |
| *Pyrrhulina semifasciata* Steindachner, 1876 |  |  |  | D | E |  |  |  | Lima et al. 2005 | INPA- 2190; 2193 | Amaz/Ori/Gui |
| *Pyrrhulina stoli* Boeseman, 1953 |  |  |  |  | E |  |  |  | Ferreira et al. 2007 | INPA- 37044 | Neg/Ori/Gui |
| **Parodontidae** |  |  |  |  |  |  |  |  |  |  |  |
| *Apareiodon* sp. |  |  |  | D |  |  |  |  | Ferreira et al. 2007 | INPA- 4541; 36838 |  |
| *Parodon bifasciatus* Eigenmann, 1912 |  |  |  | D |  |  |  |  | Ferreira et al. 2007 | INPA- 40962 | Neg/Gui |
| **Prochilodontidae** |  |  |  |  |  |  |  |  |  |  |  |
| *Prochilodus nigricans* Spix & Agassiz, 1829 |  | B |  | D |  |  |  |  | Goulding et al. 1988 | INPA- 2662; 42276 | Amaz/Neg |
| *Prochilodus rubrotaeniatus* Jardine, 1841 |  | B |  | D |  |  |  |  | Ferreira et al. 2007 | INPA- 35684; 35707 | Amaz/Ori/Gui |
| *Semaprochilodus insignis* (Jardine, 1841) |  | B |  | D |  | F |  |  | Zuanon et al. 2008 | INPA- 35608; 35970 | Amaz/Neg |
| *Semaprochilodus taeniurus* (Valenciennes, 1821) |  | B | C | D |  | F |  |  | Zuanon et al. 2008 | INPA- 35540; 39001 | Amaz/Neg |
| **Serrasalmidae** |  |  |  |  |  |  |  |  |  |  |  |
| *Catoprion mento* (Cuvier, 1819) |  | B |  | D |  |  |  |  | Rapp Py-Daniel et al. 2017 | INPA- 2456; 35670 | Amaz/Ori/Gui/Par |
| *Colossoma macropomum* (Cuvier, 1816) |  |  |  | D |  |  |  |  | INPA Fish Collection | INPA- 25751; 32906 | Amaz/Ori |
| *Metynnis altidorsalis* Ahl, 1923 |  |  |  |  |  |  |  | H | Ota et al. 2016 | INPA- 39500; 41136 | Neg/Ori/Gui |
| *Metynnis argenteus* Ahl, 1923 |  |  |  | D |  |  | G |  | Zuanon et al. 1998 | INPA- 11801; 35671 | Amaz/Ori |
| *Metynnis guaporensis* Eigenmann, 1915 |  |  |  |  |  |  |  | H | INPA Fish Collection | INPA- 23350 | Mad/Neg |
| *Metynnis hypsauchen* (Müller & Troschel, 1844) |  | B | C | D |  | F |  |  | Zuanon et al. 2008 | INPA- 10277; 30702 | Amaz/Ori/Gui/Par |
| *Metynnis lippincottianus* (Cope, 1870) |  |  |  | D |  |  |  |  | INPA Fish Collection | INPA- 6601 | Amaz/Gui |
| *Metynnis longipinnis* Zarske & Géry, 2008 |  |  |  |  |  |  |  | H | Ota et al. 2016 | INPA- 42314; | Amaz/Ori |
| *Metynnis luna* Cope, 1878 |  |  |  |  |  |  |  | H | Ota et al. 2016 | INPA- 11755; 11803 | Amaz/Neg |
| *Metynnis maculatus* (Kner, 1858) |  | B |  |  |  |  |  |  | Saint-Paul et al. 2000 | INPA- 1834 | Amaz/Par |
| *Metynnis* *melanogrammus* Ota; Rapp Py-Daniel & Jégu, 2016 |  |  |  |  |  |  |  | H | Ota et al. 2016 | INPA 52216 | Amaz/Ori |
| *Mylesinus* sp. |  |  |  |  |  |  |  | H | INPA Fish Collection | INPA- 6539 |  |
| *Myleus pacu*(Jardine, 1841) |  |  |  |  |  |  |  | H | INPA Fish Collection | INPA- 3830; 3797 | Amaz |
| *Myleus setiger* Müller & Troschel, 1844 |  | B |  | D |  |  | G |  | Ferreira et al. 2007 | INPA- 36078; 37887 | Amaz/Ori/Gui |
| *Myloplus arnoldi* Ahl, 1936 |  |  |  | D |  |  |  |  | Reis et al. 2003 | ZMB- 20812 | Amaz/Neg/Toc |
| *Myloplus asterias* (Müller & Troschel, 1844) |  | B |  | D |  |  |  |  | Lima et al. 2005 | INPA- 16104; 43722 | Amaz/Gui |
| *Myloplus lobatus* (Valenciennes, 1850) |  |  |  | D |  |  |  |  | Ferreira et al. 2007 | INPA- 8657; 10219 | Amaz/Ori |
| *Myloplus lucienae* Andrade, Ota, Bastos & Jégu, 2016 |  |  |  |  |  |  |  | H | INPA Fish Collection | INPA- 3877; 42972 | Neg |
| *Myloplus rhomboidalis* (Cuvier, 1818) | A |  |  | D |  |  | G |  | Ferreira et al. 2007 | INPA- 11702; 37885 | Amaz/Ori/Gui |
| *Myloplus rubripinnis* (Müller & Troschel, 1844) |  | B | C | D |  |  | G |  | Zuanon et al. 2008 | INPA- 2291; 35713 | Amaz/Gui |
| *Myloplus schomburgkii* (Jardine, 1841) |  | B |  | D |  | F |  |  | Goulding et al. 1988 | INPA- 3684; 30716 | Amaz/Ori/Gui |
| *Myloplus* sp. |  |  |  | D |  |  | G |  | Lima et al. 2005 | INPA- 3872; 3873 | Neg |
| *Myloplus torquatus* (Kner, 1858) |  | B |  | D |  | F |  |  | Zuanon et al. 2008 | INPA- 889; 30717 | Amaz/Neg/Ori |
| *Mylossoma albiscopum* (Cope, 1872) |  | B |  | D |  |  |  |  | Mateussi et al. 2018 | INPA- 2668; 11105 | Amaz/Ori |
| *Mylossoma aureum* (Spix & Agassiz, 1829) |  | B |  | D |  |  |  |  | INPA Fish Collection | INPA- 4510; 25758 | Amaz/Ori |
| *Piaractus brachypomus* (Cuvier, 1818) |  |  |  |  |  |  |  | H | INPA Fish Collection | INPA- 25754; 35681 | Amaz/Ori |
| *Pristobrycon calmoni* (Steindachner, 1908) |  | B |  |  |  |  |  |  | UFAM Fish Collection | UFAM- 0013 | Amaz/Ori/Gui |
| *Pristobrycon striolatus* (Steindachner, 1908) |  | B |  | D |  |  |  |  | Zuanon et al. 2008 | INPA- 14075; 16105 | Amaz/Ori/Gui |
| *Pygocentrus nattereri* Kner, 1858 |  | B |  | D |  |  |  | H | INPA Fish Collection | INPA- 25615; 35965 | Amaz/Gui/Par |
| *Pygopristis denticulatus* (Cuvier, 1819) |  | B |  | D |  |  |  |  | Zuanon et al. 1998 | INPA- 35574; 35728 | Amaz/Ori/Gui |
| *Serrasalmus altispinis* Merckx, Jégu & Santos, 2000 |  | B |  | D |  |  |  |  | Ferreira et al. 2007 | INPA- 28887; 28890 | Neg/Uat |
| *Serrasalmus altuvei* Ramírez, 1965 |  | B |  |  |  |  |  |  | Saint-Paul et al. 2000 | INPA- 1002; 1997 | Amaz/Ori |
| *Serrasalmus* *compressus* Jégu, Leão & Santos, 1991 |  | B |  | D |  |  |  |  | Ferreira et al. 2007 | INPA- 35715; 35757 | Amaz/Neg |
| *Serrasalmus eigenmanni* Norman, 1929 |  | B |  | D |  |  |  |  | Ferreira et al. 2007 | INPA- 11095; 12015 | Amaz/Ori/Gui |
| *Serrasalmus elongatus* Kner, 1858 |  | B |  | D |  |  |  |  | Saint-Paul et al. 2000 | INPA- 2748; 2752 | Amaz/Ori |
| *Serrasalmus gibbus* Castelnau, 1855 |  |  |  |  |  |  |  | H | INPA Fish Collection | INPA- 52897 | Toc/Neg |
| *Serrasalmus gouldingi* Fink & Machado-Allison, 1992 |  | B |  | D |  | F |  |  | Zuanon et al. 2008 | INPA- 16096; 28888 | Amaz/Ori |
| *Serrasalmus hastatus* Fink & Machado-Allison, 2001 |  | B |  | D |  |  |  | H | Reis et al. 2003 | INPA- 35683; 35698 | Neg |
| *Serrasalmus hollandi* Eigenmann, 1915 |  | B | C |  |  |  |  |  | INPA Fish Collection | INPA- 2288; 35561 | Amaz/Gui |
| *Serrasalmus maculatus* Kner, 1858 |  | B |  |  |  |  |  | H | INPA Fish Collection | INPA- 32909; 32910 | Amaz/Par |
| *Serrasalmus manueli* (Fernández-Yépez & Ramírez, 1967) | A | B |  | D |  | F |  |  | Zuanon et al. 2008 | INPA- 14080; 14088 | Amaz/Ori |
| *Serrasalmus rhombeus* (Linnaeus, 1766) |  | B | C | D |  | F |  |  | Zuanon et al. 2008 | INPA- 10235 | Amaz/Ori/Gui/Par |
| *Serrasalmus serrulatus* (Valenciennes, 1850) |  | B | C | D |  |  |  |  | Zuanon et al. 2008 | INPA- 28884; 28885 | Amaz/Gui |
| *Tometes makue* Jégu, Santos & Belmont-Jégu, 2002 |  | B |  |  |  |  |  | H | Andrade et al. 2013 | INPA- 4924; 43077 | Neg/Ori |
| **Tarumaniidae** |  |  |  |  |  |  |  |  |  |  |  |
| *Tarumania walkerae* de Pinna, Zuanon, Rapp Py-Daniel & Petry, 2017 |  |  |  |  | E |  |  | H | de Pinna et al. 2017 | INPA- 25747; 26246 | Neg |
| **Triportheidae** |  |  |  |  |  |  |  |  |  |  |  |
| *Agoniates anchovia* Eigenmann, 1914 |  | B | C | D |  |  |  |  | Zuanon et al. 1998 | INPA- 10271; 12345 | Amaz/Neg |
| *Agoniates halecinus* Müller & Troschel, 1845 |  | B |  | D |  |  |  |  | Zuanon et al. 2008 | INPA- 26230 | Amaz/Ori/Gui |
| *Triportheus albus* Cope, 1872 |  | B |  | D |  | F |  |  | Malabarba 2004 | INPA- 16210; 16751 | Amaz/Neg |
| *Triportheus angulatus* (Spix & Agassiz, 1829) |  | B |  | D |  |  |  | H | Malabarba 2004 | INPA- 2659; 35609 | Amaz/Gui |
| *Triportheus auritus* (Valenciennes, 1850) |  | B |  | D |  |  |  |  | INPA Fish Collection | INPA- 23358 | Amaz/Ori/Gui |
| *Triportheus rotundatus* (Jardine, 1841) |  |  |  | D |  |  |  |  | Zuanon et al. 2008 | INPA- 30697; 30709 | Amaz/Gui |
| **SILURIFORMES** |  |  |  |  |  |  |  |  |  |  |  |
| **Aspredinidae** |  |  |  |  |  |  |  |  |  |  |  |
| *Acanthobunocephalus nicoi* Friel, 1995 |  |  |  |  |  |  |  | H | INPA Fish Collection | INPA- 34949 | Neg/Ori |
| *Amaralia hypsiura* (Kner, 1855) |  |  |  | D |  |  |  | H | INPA Fish Collection | INPA- 6517 | Amaz/Gui |
| *Bunocephalus aleuropsis* Cope, 1870 |  |  |  |  |  |  |  | H | INPA Fish Collection | INPA- 29005; 37796 | Amaz/Ori |
| *Bunocephalus aloikae* Hoedeman, 1961 |  |  |  |  |  |  |  |  | INPA Fish Collection | INPA- 43008; 49727 | Neg/Ori/Gui |
| *Bunocephalus coracoideus* (Cope, 1874) |  |  |  | D |  |  |  |  | Lima et al. 2005 | INPA- 22002; 37016 | Amaz/Neg |
| *Bunocephalus* cf. *knerii* Steindachner, 1882 |  |  |  |  |  |  |  |  | Lima et al. 2005 | MZUSP- 63305 | Neg/Ori/upp Amaz |
| *Bunocephalus verrucosus* (Walbaum, 1792) | A |  | C | D | E |  |  |  | Ferreira et al. 2007 | INPA- 36090; 37037 | Amaz/Gui |
| *Pterobunocephalus depressus* (Haseman, 1911) | A |  |  |  |  |  |  |  | Rapp Py-Daniel et al. 2017 | INPA- 40961 | Amaz/Ori/Par |
| *Pterobunocephalus* sp. |  |  |  |  |  |  |  | H | MZUSP Fish Collection | MZUSP- 57208; 57209 |  |
| *Pseudobunocephalus* cf. *lundbergi* Friel, 2008 |  |  |  |  |  |  |  | H | INPA Fish Collection | INPA- 42936 | Neg/Ori |
| **Auchenipteridae** |  |  |  |  |  |  |  |  |  |  |  |
| *Ageneiosus akamai* Ribeiro, Rapp Py-Daniel & Walsh, 2017 |  |  |  |  |  |  |  | H | Ribeiro et al. 2017 | INPA- 4648 | Amaz/Neg |
| *Ageneiosus dentatus* Kner, 1857 |  |  |  |  |  |  |  | H | Ribeiro et al. 2017 | INPA- 22737 | Amaz/Ori/Gui |
| *Ageneiosus inermis* (Linnaeus, 1766) |  | B | C | D |  |  |  |  | Lima et al. 2005 | INPA- 37767 | Amaz/Ori/Gui/Par |
| *Ageneiosus intrusus* Ribeiro, Rapp Py-Daniel & Walsh, 2017 |  |  |  |  |  |  |  | H | Ribeiro et al. 2017 | INPA- 12581 | Amaz/Ori |
| *Ageneiosus lineatus* Ribeiro, Rapp Py-Daniel & Walsh, 2017 |  | B |  |  |  |  |  | H | Ribeiro et al. 2017 | INPA- 35548 | Amaz/Ori |
| *Ageneiosus polystictus* Steindachner, 1915 |  | B |  | D |  |  |  | H | Ribeiro and Rapp Py-Daniel 2010 | INPA- 34089 | Neg/Uat/Tro/Amaz main channel |
| *Ageneiosus ucayalensis* Castelnau, 1855 | A | B | C | D |  |  |  | H | Ribeiro and Rapp Py-Daniel 2010 | INPA- 25806 | Amaz/Toc |
| *Ageneiosus uranophthalmus* Ribeiro & Py-Daniel, 2010 |  |  |  |  |  | F |  | H | Ribeiro and Rapp Py-Daniel 2010 | INPA- 37811 | Amaz/Neg |
| *Ageneiosus vittatus* Steindachner, 1908 | A |  |  | D |  |  |  | H | Ribeiro and Rapp Py-Daniel 2010 | INPA- 12530 | Neg/Ori/upp Amaz |
| *Asterophysus batrachus* Kner, 1858 |  |  |  | D |  |  |  | H | Birindelli 2014 | INPA- 15680; 24119 | Neg/Ori/upp Amaz |
| *Auchenipterichthys coracoideus* (Eigenmann & Allen, 1942) |  | B |  | D |  | F |  |  | Ferreira et al. 2007 | INPA- 35720; 37779 | Amaz/Neg |
| *Auchenipterichthys longimanus* (Günther, 1864) |  | B |  | D |  | F |  | H | Ferraris-Jr et al. 2005 | INPA- 26234; 37766 | Amaz/Ori |
| *Auchenipterichthys punctatus* (Valenciennes, 1840) |  | B |  | D | E |  |  | H | Calegari et al. 2014 | INPA- 26415; 35955 | Neg/Ori/upp Amaz |
| *Auchenipterus ambyiacus* Fowler, 1915 |  |  |  |  |  |  |  | H | MZUSP Fish Collection | MZUSP- 30606; 30610 | Neg/Ori/upp Amaz |
| *Auchenipterus brachyurus* (Cope, 1878) |  |  |  |  |  |  |  | H | INPA Fish Collection | INPA- 25523; 25734 | Neg/upp Amaz |
| *Auchenipterus britskii* Ferraris & Vari, 1999 |  | B |  | D |  |  |  |  | Ferreira et al. 2007 | INPA- 12683 | Neg/mid Amaz |
| *Auchenipterus nuchalis* (Spix & Agassiz, 1829) | A | B |  | D |  | F |  |  | Goulding et al. 1988 | INPA- 9736; 9941 | Amaz/Ori/Gui |
| *Centromochlus altae* Fowler, 1945 |  |  |  |  |  |  |  | H | INPA Fish Collection | INPA- 39347 | Neg/Ori |
| *Centromochlus* aff. *concolor* (Mees, 1974) |  |  |  |  |  |  |  | H | INPA Fish Collection | INPA- 30320 | Neg/Gui |
| *Centromochlus existimatus* Mees, 1974 |  |  |  |  |  |  |  | H | Reis et al. 2003 | MZUSP- 53637; 57647 | Amaz/Neg |
| *Centromochlus heckelii* (De Filippi, 1853) | A | B | C | D |  | F |  |  | Goulding et al. 1988 | INPA- 10274; 37772 | Amaz/Ori |
| *Centromochlus macracanthus* Soares-Porto, 2000 |  | B |  | D |  | F |  | H | Vari and Calegari 2014 | INPA- 30687; 42995 | Neg |
| *Centromochlus reticulatus* (Mees, 1974) |  | B |  | D |  |  |  |  | Reis et al. 2003 | MZUSP- 88789 | Amaz/Gui |
| *Gelanoglanis nanonocticolus* Soares-Porto, Walsh, Nico & Netto, 1999 |  |  |  |  |  |  |  | H | Reis et al. 2003 | MZUSP- 28308 | Neg/Ori |
| *Liosomadoras oncinus* (Jardine, 1841) |  |  |  | D |  |  |  | H | Birindelli and Zuanon 2012 | INPA- 35453; 35536 | Amaz/Ori |
| *Pseudepapterus cucuhyensis* Böhlke, 1951 | A |  |  |  |  | F |  |  | Thomé-Souza and Chao 2004 | INPA- 17965; 37807 | Amaz/Neg |
| *Pseudepapterus hasemani* (Steindachner, 1915) | A |  | C | D |  |  |  |  | INPA Fish Collection | INPA- 12501; 12689 | Amaz/Neg |
| *Spinipterus* sp. |  |  |  | D |  |  |  |  | Ferreira et al. 2007 | INPA- 42816; 43053 | Neg/Ori/Gui |
| *Tatia aulopygia* (Kner, 1858) |  |  |  |  |  |  |  | H | INPA Fish Collection | INPA- 9993; 36026 | Amaz/Neg |
| *Tatia brunnea* Mees, 1974 |  | B |  | D | E |  |  | H | Vari and Calegari 2014 | INPA- 41240 | Amaz/Gui |
| *Tatia gyrina* (Eigenmann & Allen, 1942) |  |  |  | D |  |  |  |  | Vari and Calegari 2014 | INPA- 29421; 39437 | Amaz/Gui |
| *Tatia intermedia* (Steindachner, 1877) |  | B |  | D | E |  |  |  | Zuanon et al. 1998 | INPA- 36037; 36193 | Amaz/Ori/Gui |
| *Tatia nigra* Sarmento-Soares & Martins-Pinheiro, 2008 |  |  |  |  |  | F |  | H | Vari and Calegari 2014 | INPA- 35564; 35958 | Amaz/Ori |
| *Tatia strigata* Soares-Porto, 1995 |  | B |  | D | E |  |  | H | Vari and Calegari 2014 | INPA- 30728 | Amaz/Ori |
| *Tetranematichthys barthemi* Peixoto & Wosiacki, 2010 |  |  |  |  |  |  |  | H | Peixoto and Wosiacki 2010 | INPA- 14163 | Neg/low Amaz |
| *Tetranematichthys quadrifilis* (Kner, 1858) |  |  |  | D | E |  |  |  | Zuanon et al. 2008 | INPA- 1275; 26237 | Amaz/Ori |
| *Tetranematichthys wallacei* Vari & Ferraris, 2006 |  | B |  | D | E |  |  | H | Birindelli 2014 | INPA- 37828; 37864 | Amaz/Ori |
| *Trachelyichthys decaradiatus* Mees, 1974 |  |  |  | D |  |  |  |  | Rapp Py-Daniel et al. 2017 | INPA- 37007 | Neg/Ori/Gui |
| *Trachelyopterichthys taeniatus* (Kner, 1858) |  |  |  | D |  |  |  |  | Zuanon et al. 2008 | INPA- 26238; 35666 | Amaz/Ori |
| *Trachelyopterus ceratophysus* (Kner, 1858) |  |  |  | D |  |  |  | H | Birindelli 2014 | MZUSP- 52087 | Neg/mid Amaz/Par |
| *Trachelyopterus galeatus* (Linnaeus, 1766) |  | B |  | D |  |  |  |  | Zuanon et al. 1998 | INPA- 16141; 30759 | Amaz/Ori/Gui/Par |
| *Trachycorystes porosus* Eigenmann & Eigenmann, 1888 |  |  |  |  |  |  |  | H | Birindelli 2014 | MZUSP- 75365 |  |
| *Trachycorystes trachycorystes* (Valenciennes, 1840) |  | B |  | D |  |  |  |  | Birindelli 2014 | INPA- 26406; 26413 | Amaz/Ori/Gui |
| *Tympanopleura atronasus* (Eigenmann & Eigenmann, 1888) |  |  |  | D |  | F |  | H | INPA Fish Collection | INPA- 16598 | Amaz/Neg |
| *Tympanopleura brevis* (Steindachner, 1881) |  |  |  |  |  |  |  | H | Ribeiro and Rapp Py-Daniel 2010 | INPA- 28808 | Amaz/Neg |
| *Tympanopleura longipinna* Walsh, Ribeiro & Rapp Py-Daniel, 2015 |  |  |  |  |  |  |  | H | Walsh et al. 2015 | INPA 34078 | Amaz/Neg |
| *Tympanopleura piperatus* Eigenmann, 1912 | A |  |  | D |  |  |  | H | Calegari et al. 2014 | INPA- 10263; 36757 | Amaz/Gui |
| *Tympanopleura rondoni* (Miranda Ribeiro, 1914) |  |  |  |  |  |  |  | H | Walsh et al. 2015 | INPA- 26537 | Amaz/Neg |
| **Callichthyidae** |  |  |  |  |  |  |  |  |  |  |  |
| *Aspidoras*sp. 'demini' |  |  |  |  |  |  |  | H | INPA Fish Collection | INPA- 35095; 51969 | Neg |
| *Callichthys callichthys* (Linnaeus, 1758) |  |  |  | D | E |  |  |  | Ferreira et al. 2007 | INPA- 10755; 15087 | Amaz/Ori/Gui/Par/SaF |
| *Callichthys serralabium* Lehmann & Reis, 2004 |  |  |  | D |  |  |  | H | Lehmann and Reis 2004 | INPA- 38893 | Neg/Ori |
| *Corydoras adolfoi* Burgess, 1982 |  |  |  |  |  |  |  | H | Tencatt and Ohara 2016 | INPA- 22959 | Neg |
| *Corydoras aeneus* (Gill, 1858) |  |  |  |  | E |  |  |  | INPA Fish Collection | INPA- 43808; 43832 | Amaz/Ori/Gui/Par |
| *Corydoras amandajanea* Sands, 1995 |  |  |  |  |  |  |  | H | Reis et al. 2003 | LIVCM- 1994.4.21 | Neg |
| *Corydoras arcuatus* Elvim, 1939 |  |  |  |  |  |  |  | H | INPA Fish Collection | INPA- 9166; 9215 | Neg/upp Amaz |
| *Corydoras bicolor* Nijssen & Isbrücker, 1967 |  |  |  |  |  |  |  |  | INPA Fish Collection | INPA- 8093 | Neg/Gui |
| *Corydoras blochi* Nijssen, 1971 |  |  |  | D |  |  |  |  | Ferreira et al. 2007 | INPA- 1289 | Amaz/Ori/Gui |
| *Corydoras bondi* Gosline, 1940 |  |  |  | D |  |  |  |  | Ferreira et al. 2007 | INPA- 1288; 1639 | Neg/Ori/Gui |
| *Corydoras burgessi* Axelrod, 1987 |  |  |  |  |  |  |  | H | Reis et al. 2003 | INPA- 43013 | Neg |
| *Corydoras* cf*. breei* Isbrücker & Nijssen, 1992 |  |  |  |  |  |  |  |  | INPA Fish Collection | INPA- 8129; 8133 | Neg/Gui |
| *Corydoras crimmeni* Grant, 1997 |  |  |  | D |  |  |  | H | Reis et al. 2003 | MZUSP- 52490 | Neg (Bra) |
| *Corydoras crypticus* Sands, 1995 |  |  |  |  |  |  |  | H | Reis et al. 2003 | LIVCM- 1994.4.29 | Neg |
| *Corydoras davidsandsi* Black, 1987 |  |  |  |  |  |  |  | H | Tencatt and Brito 2016 | MZUSP- 38632 | Neg |
| *Corydoras desana* Lima & Sazima, 2017 |  |  |  |  |  |  |  | H | Lima and Sazima 2017 | MZUSP- 121044; | Neg |
| *Corydoras duplicareus* Sands, 1995 |  |  |  |  |  |  |  | H | Reis et al. 2003 | LIVCM- 1994.4.36 | Neg |
| *Corydoras granti* Tencatt, Lima & Britto, 2019 |  |  |  |  |  |  |  | H | Tencatt et al. 2019 | MNRJ- 51193 | Upp Amaz/mid Amaz/Neg |
| *Corydoras hastatus* Eigenmann & Eigenmann, 1888 |  |  |  |  |  |  |  |  | INPA Fish Collection | INPA- 33256 | Amaz/Par |
| *Corydoras imitator* Nijssen & Isbrücker, 1983 |  |  |  |  |  |  |  | H | Reis et al. 2003 | INPA- 9171; 9237 | Neg |
| *Corydoras incolicana* Burgess, 1993 |  |  |  |  |  |  |  | H | Tencatt and Ohara 2016 | MZUSP- 45717 | Neg |
| *Corydoras kanei* Grant, 1998 |  |  |  | D |  |  |  | H | Ferreira et al. 2007 | MZUSP- 52489 | Neg (Bra) |
| *Corydoras melanistius* Regan, 1912 |  |  |  | D |  |  |  |  | Ferreira et al. 2007 | INPA- 39404 | Neg/Ori/Gui |
| *Corydoras melini* Lönnberg & Rendahl, 1930 |  |  |  | D |  |  |  | H | Tencatt and Ohara 2016 | NRM- 11091 | Neg/Ori |
| *Corydoras nijsseni* Sands, 1989 |  |  |  |  |  |  |  | H | Reis et al. 2003 | RMNH- 31625 | Neg |
| *Corydoras osteocarus* Böhlke, 1951 |  |  |  | D |  |  |  |  | Ferreira et al. 2007 | INPA- 7910; 7916 | Neg/Ori/Gui |
| *Corydoras parallelus* Burgess, 1993 |  |  |  |  |  |  |  | H | Tencatt and Brito 2016 | MZUSP- 45716 | Neg |
| *Corydoras potaroensis* Myers, 1927 |  |  |  | D |  |  |  |  | Ferreira et al. 2007 | INPA- 8095 | Neg/Gui |
| *Corydoras pulcher*Isbrücker & Nijssen, 1973 |  |  |  |  |  |  |  | H | INPA Fish Collection | INPA- 40933 | Pur/Neg |
| *Corydoras rabauti* La Monte, 1941 |  |  |  |  |  |  |  | H | Reis et al. 2003 | USNM- 16272 | Neg/upp Amaz |
| *Corydoras robineae* Burgess, 1983 |  |  |  |  |  |  |  | H | Tencatt and Brito 2016 | MZUSP- 27175 | Neg |
| *Corydoras serratus* Sands, 1995 |  |  |  |  |  |  |  | H | Reis et al. 2003 | LIVCM- 1994.4.38 | Neg |
| *Corydoras tukano* Britto & Lima, 2003 |  |  |  | D |  |  |  | H | Brito and Lima 2003 | INPA- 21423 | Neg |
| *Corydoras* sp. 'tacutu' |  |  |  | D |  |  |  |  | Ferreira et al. 2007 | INPA- 40925 | Neg (Bra) |
| *Hoplosternum littorale* (Hancock, 1828) |  | B |  |  |  |  |  |  | INPA Fish Collection | INPA- 13066; 25614 | Amaz/Ori/Gui/Par |
| *Megalechis picta* (Müller & Troschel, 1849) |  |  |  |  |  |  |  | H | INPA Fish Collection | INPA- 38918; 51361 | Amaz/Ori/Gui |
| *Megalechis thoracata* (Valenciennes, 1840) |  |  |  | D | E |  |  |  | Lima et al. 2005 | INPA- 3644; 30690 | Amaz/Ori/Gui/Par |
| **Cetopsidae** |  |  |  |  |  |  |  |  |  |  |  |
| *Cetopsidium morenoi*(Fernández-Yépez, 1972) |  |  |  |  |  |  |  | H | INPA Fish Collection | INPA- 30316; 36183 | Neg/Ori |
| *Cetopsidium minutum* (Eigenmann, 1912) |  |  |  |  |  |  |  | H | INPA Fish Collection | INPA- 6502; 6506 | Neg/Gui |
| *Cetopsidium pemon* Vari, Ferraris & de Pinna, 2005 |  |  |  | D |  |  |  |  | INPA Fish Collection | INPA- 16693; 16694 | Neg/Ori |
| *Cetopsidium soniae* Vari & Ferraris, 2009 |  |  |  |  |  |  |  | H | Vari and Ferraris-Jr 2009 | CSBD- F1665 | Neg (Bra) |
| *Cetopsis candiru* Spix & Agassiz, 1829 |  |  |  |  |  |  |  | H | Vari et al. 2005 | MZUSP- 30696 | Amaz/Neg |
| *Cetopsis coecutiens* (Lichtenstein, 1819) |  | B | C |  |  |  |  | H | Vari et al. 2005 | INPA- 10279; 43059 | Amaz/Ori/Toc |
| *Cetopsis* *parma* Oliveira, Vari & Ferraris, 2001 |  |  |  | D |  |  |  |  | Lima et al. 2005 | MZUSP- 79993 | Neg/upp Amaz |
| *Denticetopsis macilenta* (Eigenmann, 1912) |  |  |  | D | E |  |  |  | Lima et al. 2005 | INPA- 25373; 25408 | Neg/Gui |
| *Denticetopsis praecox* (Ferraris & Brown, 1991) |  |  |  |  |  |  |  | H | Vari et al. 2005 | USNM- 309184 | Neg/Amaz main channel |
| *Denticetopsis royeroi* Ferraris, 1996 |  |  |  |  |  |  |  | H | Vari et al. 2005 | MBUCV- V-26785 | Neg |
| *Denticetopsis sauli* Ferraris, 1996 |  |  |  |  |  |  |  | H | Vari et al. 2005 | ANSP- 161432 | Neg |
| *Denticetopsis seducta* Vari, Ferraris & de Pinna, 2005 |  |  |  | D | E |  |  |  | Zuanon et al. 2015 | INPA- 29818; 30457 | Amaz/Neg |
| *Helogenes marmoratus* Günther, 1863 |  |  |  | D | E |  |  |  | Ferreira et al. 2007 | INPA- 12877; 14205 | Amaz/Ori/Gui |
| **Doradidae** |  |  |  |  |  |  |  |  |  |  |  |
| *Acanthodoras cataphractus* (Linnaeus, 1758) |  |  |  |  | E |  |  | H | Birindelli 2014 | INPA- 29951 | Amaz/Gui |
| *Acanthodoras depressus* (Steindachner, 1881) |  |  |  |  |  |  |  | H | Reis et al. 2003 | NMW- 46870 | Neg |
| *Acanthodoras spinosissimus* (Eigenmann & Eigenmann, 1888) |  | B |  | D |  |  |  |  | Goulding et al. 1988 | INPA- 16098 | Amaz/Gui |
| *Agamyxis pectinifrons* (Cope, 1870) |  |  |  |  |  |  |  |  | INPA Fish Collection | INPA- 18595 | Amaz/Neg |
| *Amblydoras* *affinis* (Kner, 1855) |  |  |  |  |  |  |  | H | Reis et al. 2003 | INPA- 20179 | Amaz/Ori/Gui |
| *Anduzedoras oxyrhynchus* (Valenciennes, 1821) | A | B |  | D |  | F |  | H | Birindelli 2014 | INPA- 26412 | Amaz/Ori/Gui |
| *Anadoras grypus* (Cope, 1872) |  |  |  |  |  |  |  | H | INPA Fish Collection | INPA- 22423; 27043 | Neg/upp Amaz |
| *Anadoras weddellii* (Castelnau, 1855) |  |  |  |  |  |  |  | H | INPA Fish Collection | INPA-23367 | Par/Mad/Neg |
| *Astrodoras asterifrons* (Kner, 1853) | A | B | C |  |  | F |  |  | Zuanon et al. 1998 | INPA- 9994; 35568 | Amaz/Neg |
| *Centrodoras brachiatus* (Cope, 1872) | A | B |  |  |  |  |  |  | Chao 2001 | INPA- 22003; | Amaz/Neg |
| *Centrodoras hasemani* (Steindachner, 1915) | A | B |  |  |  |  |  | H | Birindelli 2014 | INPA- 11338 | Neg |
| *Doras carinatus*(Linnaeus, 1766) |  |  |  |  |  |  |  | H | INPA Fish Collection | INPA- 51895 | Amaz/Ori/Gui |
| *Doras higuchii*Sabaj Pérez & Birindelli, 2008 |  |  |  |  |  |  |  | H | INPA Fish Collection | INPA-50983 | low Amaz/Neg |
| *Doras phlyzakion* Sabaj Pérez & Birindelli, 2008 |  |  |  |  |  |  |  | H | Sabaj and Birindelli 2008 | INPA- 36081; 39442 | Neg/mid Amaz |
| *Hassar orestis* (Steindachner, 1875) | A |  |  |  |  | F |  |  | Thomé-Souza and Chao 2004 | INPA- 17706; 17742 | Amaz/Ori/Gui |
| *Hassar wilderi* Kindle, 1895 | A |  |  | D |  |  |  |  | Ferreira et al. 2007 | INPA- 2003 | Neg/Toc |
| *Hemidoras boulengeri* (Steindachner, 1915) |  | B | C |  |  |  |  |  | INPA Fish Collection | INPA- 11333; 36811 | Amaz/Neg |
| *Hemidoras morei* (Steindachner, 1881) | A | B | C | D |  |  |  | H | Birindelli 2014 | INPA- 11311; 12678 | Neg/mid Amaz |
| *Hemidoras morrisi* Eigenmann, 1925 | A |  |  |  |  |  |  |  | Thomé-Souza and Chao 2004 | INPA- 27050; 36515 | Neg/upp Amaz |
| *Hemidoras stenopeltis* (Kner, 1855) | A |  |  |  |  |  |  |  | Thomé-Souza and Chao 2004 | INPA- 12154; 22170 | Amaz/Neg |
| *Hemidoras stuebelii* (Steindachner, 1882) | A |  |  |  |  |  |  |  | Thomé-Souza and Chao 2004 | INPA- 27045; 27051 | Neg/upp Amaz |
| *Leptodoras acipenserinus* (Günther, 1868) | A |  |  |  |  |  |  | H | Reis et al. 2003 | BMNH- 1867.6.13.32. | Neg/upp Amaz |
| *Leptodoras cataniai* Sabaj Pérez, 2005 | A |  |  |  |  |  |  | H | Birindelli et al. 2008 | INPA- 17709; 17717 | Amaz/Ori |
| *Leptodoras copei* (Fernández-Yépez, 1968) | A |  |  |  |  |  |  | H | Sabaj 2005 | ANSP- 180893; 180896 | Neg/Ori/upp Amaz |
| *Leptodoras hasemani* (Steindachner, 1915) | A |  |  |  |  |  |  |  | Sabaj 2005 | INPA- 17699; 35744 | Neg/Ori/Gui |
| *Leptodoras juruensis* Boulenger, 1898 | A |  |  |  |  |  |  | H | INPA Fish Collection | INPA- 26900; 36788 | Neg/upp Amaz |
| *Leptodoras linnelli* Eigenmann, 1912 | A |  |  |  |  |  |  |  | Birindelli et al. 2008 | INPA- 2002; 36779 | Amaz/Ori/Gui |
| *Leptodoras praelongus* (Myers & Weitzman, 1956) | A |  |  |  |  | F |  | H | Sabaj 2005 | INPA- 12554; 17704 | Neg/Ori |
| *Lithodoras dorsalis* (Valenciennes, 1840) | A |  |  |  |  |  |  | H | Chao 2001 | MZUSP- 13993.0 | Amaz/Gui |
| *Megalodoras uranoscopus* (Eigenmann & Eigenmann, 1888) |  | B |  | D |  |  |  |  | Goulding et al. 1988 | INPA- 22165; 22421 | Amaz/Gui/Toc |
| *Nemadoras elongatus* (Boulenger, 1898) |  | B |  | D |  |  |  | H | Birindelli 2014 | INPA- 11321; 18624 | Amaz/Neg |
| *Nemadoras hemipeltis* (Eigenmann, 1925) |  | B |  |  |  |  |  |  | INPA Fish Collection | INPA- 25776; | Amaz/Neg |
| *Nemadoras humeralis* (Kner, 1855) |  |  |  |  |  |  |  | H | Birindelli 2014 | INPA- 25814; 36525 | Amaz/Neg |
| *Ossancora asterophysa* Birindelli & Sabaj Pérez, 2011 |  |  |  |  |  |  |  |  | INPA Fish Collection | INPA- 9640; 23012 | Amaz/Neg |
| *Ossancora fimbriata* (Kner, 1855) | A | B |  | D |  |  |  |  | INPA Fish Collection | INPA- 12379; 12684 | Amaz/Neg |
| *Ossancora punctata* (Kner, 1855) |  |  |  |  |  |  |  |  | INPA Fish Collection | INPA- 22137; 22228 | Amaz/Par |
| *Oxydoras niger* (Valenciennes, 1821) |  | B |  | D |  | F |  | H | Birindelli 2014 | INPA- 21570; 42278 | Amaz/Gui/SaF |
| *Physopyxis ananas* Sousa & Py-Daniel, 2005 |  |  |  | D | E |  |  | H | Sousa and Rapp Py-Daniel 2005 | INPA- 13420; 18448 | Amaz/Gui |
| *Physopyxis cristata* Sousa & Py-Daniel, 2005 |  |  |  | D | E |  |  | H | Sousa and Rapp Py-Daniel 2005 | INPA- 25061; 25062 | Neg |
| *Physopyxis lyra* Cope, 1872 |  |  |  | D | E |  |  |  | Sousa and Rapp Py-Daniel 2005 | INPA- 39459 | Amaz/Gui |
| *Platydoras costatus* (Linnaeus, 1758) | A | B |  | D | E |  |  |  | Goulding et al. 1988 | INPA- 16097; 21571 | Amaz/Ori/Gui |
| *Platydoras hancocki* (Valenciennes, 1840) | A |  |  | D |  |  |  |  | Rapp Py-Daniel et al. 2017 | INPA- 9932; 35956 | Neg/Ori/Gui |
| *Pterodoras granulosus* (Valenciennes, 1821) | A | B |  |  |  |  |  |  | Thomé-Souza and Chao 2004 | INPA- 18627; 20805 | Amaz/Gui/Par |
| *Rhinodoras armbrusteri* Sabaj, Taphorn & Castillo, 2008 | A |  |  |  |  |  |  | H | Sabaj et al. 2008 | ANSP- 179096 | Neg/Gui |
| *Rhynchodoras woodsi* Glodek, 1976 | A |  |  |  |  |  |  | H | Ferreira et al. 2007 | INPA- 34987; 36507 | Amaz/Gui |
| *Scorpiodoras heckelii* (Kner, 1855) | A |  |  | D |  |  |  |  | Sousa and Birindelli 2011 | INPA- 30138; 36008 | Neg/Ori |
| *Tenellus leporhinus* (Eigenmann, 1912) | A | B |  | D |  |  |  |  | Thomé-Souza and Chao 2004 | INPA- 43072 | Amaz/Ori/Gui |
| *Tenellus ternetzi* (Eigenmann, 1925) | A | B | C |  |  | F |  | H | Birindelli 2014 | INPA- 17964; 34988 | Amaz/Ori/Gui |
| *Tenellus trimaculatus* (Boulenger, 1898) | A | B | C | D |  | F |  | H | INPA Fish Collection | INPA- 11327; 18006 | Amaz/Ori/Gui |
| *Trachydoras brevis* (Kner, 1853) | A |  |  | D |  | F |  | H | Birindelli 2014 | INPA- 12622 | Amaz/Gui |
| *Trachydoras gepharti* Sabaj & Arce, 2017 | A |  |  |  |  |  |  | H | Sabaj and Hernández 2017 | AUM- 43647 | Amaz/Ori/Gui |
| *Trachydoras microstomus* (Eigenmann, 1912) | A | B | C |  |  | F |  | H | Birindelli 2014 | INPA- 10301; 39466 | Amaz/Ori/Gui |
| *Trachydoras nattereri* (Steindachner, 1881) | A | B |  | D |  |  |  | H | Birindelli 2014 | INPA- 17752; 18623 | Amaz/Gui |
| *Trachydoras steindachneri* (Perugia, 1897) | A |  | C |  |  |  |  |  | INPA Fish Collection | INPA- 11309; 18632 | Amaz/Neg |
| **Heptapteridae** |  |  |  |  |  |  |  |  |  |  |  |
| *Brachyglanis frenata* Eigenmann, 1912 |  |  |  |  |  |  |  | H | INPA Fish Collection | INPA- 14226 | Amaz/Ori/Gui |
| *Brachyglanis magoi*Fernández-Yépez, 1967 |  |  |  |  |  |  |  | H | INPA Fish Collection | INPA-49594 | Neg/Ori |
| *Brachyglanis melas* Eigenmann, 1912 |  |  |  |  | E |  | G |  | Anjos and Zuanon 2007 | INPA- 25346 | Neg/Gui |
| *Brachyglanis microphthalmus* Bizerril, 1991 |  |  |  |  | E |  |  |  | Zuanon et al. 2015 | INPA- 29982 | Neg/Tro |
| *Brachyglanis nocturnus* Myers, 1928 |  |  |  |  |  |  | G | H | Ferraris-Jr 2007 | CAS- 63393 | Neg |
| *Brachyrhamdia heteropleura* (Eigenmann, 1912) |  |  |  | D | E |  | G | H | Reis et al. 2003 | INPA- 8111 | Neg/Gui |
| *Brachyrhamdia rambarrani* (Axelrod & Burgess, 1987) |  |  |  |  |  |  |  | H | Reis et al. 2003 | INPA- 9188 | Neg |
| *Cetopsorhamdia insidiosa* (Steindachner, 1915) |  |  |  |  |  |  | G |  | Ferreira et al. 2007 | INPA- 11833; 22839 | Neg (Bra) |
| *Chasmocranus longior* Eigenmann, 1912 |  |  |  |  |  |  | G | H | Reis et al. 2003 | INPA- 22827 | Amaz/Ori/Gui |
| *Gladioglanis conquistador* Lundberg, Bornbusch & Mago-Leccia, 1991 |  |  |  | D | E |  |  | H | Rocha et al. 2008b | INPA- 28655; 30436 | Amaz/Neg |
| *Gladioglanis machadoi* Ferraris & Mago-Leccia, 1989 |  |  |  | D |  |  |  | H | Rocha et al. 2008b | INPA- 28958; 42792 | Amaz/Ori |
| *Goeldiella eques* (Müller & Troschel, 1849) |  | B |  | D |  |  |  | H | Birindelli 2014 | INPA- 30090 | Amaz/Ori/Gui |
| *Heptapterus* sp. |  |  |  | D |  |  |  |  | Ferreira et al. 2007 | INPA- 8037 |  |
| *Imparfinis hasemani* Steindachner, 1915 |  |  |  |  |  |  | G |  | Ferreira et al. 2007 | INPA- 11843; 11859 | Neg/Tap |
| *Imparfinis pristos* Mees & Cala, 1989 |  |  |  |  | E |  |  |  | Zuanon et al. 2015 | INPA- 27724; 30060 | Neg/Ori |
| *Imparfinis stictonotus* (Fowler, 1940) |  |  |  |  |  |  |  |  | INPA Fish Collection | INPA- 16758 | Amaz/Neg |
| *Leptorhamdia essequibensis* (Eigenmann, 1912) |  |  |  |  |  |  | G |  | INPA Fish Collection | INPA- 8004; 10764 | Neg/Gui/Toc |
| *Leptorhamdia marmorata* Myers, 1928 |  |  |  |  |  |  | G | H | Ferraris-Jr 2007 | CAS- 63671 | Neg |
| *Mastiglanis asopos* Bockmann, 1994 |  |  |  | D | E | F |  |  | Lima et al. 2005 | INPA- 20077; 27876 | Amaz/Ori |
| *Mastiglanis* sp. ‘rio branco’ |  |  |  | D |  | F |  |  | Ferreira et al. 2007 | INPA- 11685; 36065 | Neg |
| *Myoglanis koepckei* Chang, 1999 |  |  |  |  |  |  |  | H | Rocha et al. 2008b | INPA- 14224; 29937 | Neg/upp Amaz |
| *Myoglanis* cf. *potaroensis* Eigenmann, 1912 |  |  |  | D |  |  |  |  | Lima et al. 2005 | MZUSP- 74973; 80138 | Neg/Gui |
| *Myoglanis* sp. |  |  |  | D |  |  |  |  | Goulding et al. 1988 | MZUSP- 30834 | Neg (Bra) |
| *Nannorhamdia* sp. |  |  |  | D |  |  |  |  | Ferreira et al. 2007 | INPA- 7913; 29678 |  |
| *Nemuroglanis lanceolatus* Eigenmann & Eigenmann, 1889 |  |  |  | D | E |  |  |  | Kemenes and Forsberg 2014 | INPA- 36123 | Neg/upp Amaz |
| *Nemuroglanis pauciradiatus* Ferraris, 1988 |  |  |  |  | E |  |  |  | INPA Fish Collection | INPA- 11838; 29910 | Neg/Ori |
| *Nemuroglanis* sp. |  |  |  |  |  |  |  |  | Zuanon et al. 2015 | INPA- 27936; 28624 | Neg |
| *Pariolius armillatus* Cope, 1872 |  |  |  |  |  |  |  | H | INPA Fish Collection | INPA- 49920 | Neg/upp Amaz |
| *Pariolius* sp. |  |  |  | D |  |  |  |  | Lima et al. 2005 | MZUSP- 80136; 85152 |  |
| *Phenacorhamdia tenuis*(Mees, 1986) |  |  |  |  | E |  | G | H | INPA Fish Collection | INPA- 16589 | Neg/Ori/Gui |
| *Pimelodella altipinnis* (Steindachner, 1864) |  |  |  |  |  |  |  | H | INPA Fish Collection | INPA- 12599; 15913 | Neg/Gui |
| *Pimelodella breviceps* (Kner, 1858) |  |  |  |  |  |  |  | H | Ferraris-Jr 2007 | NMW- 45615 | Neg |
| *Pimelodella cristata* (Müller & Troschel, 1849) | A |  |  | D |  | F |  |  | Lima et al. 2005 | INPA- 1292; 16196 | Amaz/Ori/Gui |
| *Pimelodella gracilis* (Valenciennes, 1835) |  |  |  |  |  |  |  | H | INPA Fish Collection | INPA- 16106 | Amaz/Ori/Par |
| *Pimelodella megalops* Eigenmann, 1912 | A |  |  | D |  | F |  |  | Ferreira et al. 2007 | INPA- 36109; 36315 | Neg/Gui |
| *Rhamdia foina* (Müller & Troschel, 1849) |  |  |  | D |  |  |  | H | Reis et al. 2003 | INPA- 7988 | Neg/Gui/low Amaz |
| *Rhamdia laukidi* Bleeker, 1858 |  |  |  | D | E |  |  |  | Lima et al. 2005 | INPA- 38847; 39335 | Amaz/Ori/Gui |
| *Rhamdia muelleri* (Günther, 1864) |  |  |  |  |  | F |  | H | Ferraris-Jr 2007 | NMW- 45790 | Amaz/Ori/Gui/Par |
| *Rhamdia quelen* (Quoy & Gaimard, 1824) |  |  |  |  | E |  |  |  | Zuanon et al. 2015 | INPA- 14294; 27907 | South America |
| **Loricariidae** |  |  |  |  |  |  |  |  |  |  |  |
| *Acanthicus hystrix* Spix & Agassiz, 1829 |  |  |  | D |  |  |  |  | Ferreira et al. 2007 | INPA- 6305 | Amaz/Ori/Toc |
| *Acestridium colombiense*Retzer, 2005 |  |  |  |  |  |  |  | H | INPA Fish Collection | INPA- 49527; 49907 | Neg/Ori |
| *Acestridium dichromum* Retzer, Nico & Provenzano, 1999 |  |  |  |  |  |  |  | H | Rodriguez and Reis 2007 | USNM- 269949 | Amaz/Ori |
| *Acestridium discus* Haseman, 1911 |  |  |  | D | E |  |  | H | Rodriguez and Reis 2007 | FMNH- 54339 | Neg/Tro/Amaz main channel |
| *Acestridium gymnogaster*Reis & Lehmann, 2009 |  |  |  |  |  |  |  | H | INPA Fish Collection | INPA- 54837 | Mad/Neg |
| *Acestridium martini* Retzer, Nico & Provenzano, 1999 |  |  |  |  | E |  |  | H | Rodriguez and Reis 2007 | MZUSP- 26820; 61945 | Neg/Ori |
| *Ancistrus dolichopterus* Kner, 1854 |  |  |  | D | E |  | G |  | Zuanon et al. 2008 | INPA- 30669; 38779 | Neg/Gui |
| *Ancistrus hoplogenys* (Günther, 1864) |  |  |  | D | E |  |  |  | Zuanon et al. 2015 | INPA- 15090; 15093 | Amaz/Gui/Par |
| *Ancistrus maximus* de Oliveira; Zuanon; Zawadzki & Rapp-Py Daniel, 2015 |  |  |  |  |  |  |  | H | de Oliveira et al. 2015 | INPA- 25629 | Neg (Bra) |
| *Ancistrus nudiceps* (Müller & Troschel, 1848) |  |  |  | D |  |  |  |  | Ferraris-Jr 2007 | ZMB- 3180 | Neg/Gui |
| *Aphanotorulus emarginatus* (Valenciennes, 1840) |  |  |  |  |  | F |  | H | Ray and Armbruster 2016 | INPA- 2377; 16595 | Amaz/Ori/Gui |
| *Aphanotorulus horridus* (Kner, 1854) |  |  |  | D |  |  |  |  | Ferreira et al. 2007 | INPA- 418; 6074 | Amaz/Neg |
| *Aphanotorulus unicolor*(Steindachner, 1908) |  |  |  |  |  |  |  | H | INPA Fish Collection | INPA- 14807 | Upp Amaz/Neg |
| *Apistoloricaria* sp. | A |  |  |  |  |  |  |  | Thomé-Souza and Chao 2004 | INPA- 16213; 16220 |  |
| *Chaetostoma jegui* Rapp Py-Daniel, 1991 |  |  |  | D |  |  |  |  | Ferreira et al. 2007 | INPA- 8151; 33840 | Neg (Bra) |
| *Dentectus* sp. |  |  |  |  |  |  |  | H | INPA Fish Collection | INPA-37820 |  |
| *Dekeyseria amazonica* Rapp Py-Daniel, 1985 |  |  |  | D |  |  |  |  | INPA Fish Collection | INPA- 205; 25782 | Amaz/Neg |
| *Dekeyseria picta* (Kner, 1854) |  |  |  |  |  |  |  | H | Silva and Rapp Py-Daniel 2018 | INPA- 379; 42971 | Neg/Ori |
| *Dekeyseria scaphirhyncha* (Kner, 1854) |  | B |  | D | E |  |  |  | Zuanon et al. 1998 | INPA- 2376; 2435 | Neg/Mad/Pur/Ori |
| *Dekeyseria* sp. |  |  |  |  |  |  |  | H | INPA Fish Collection | INPA- 42763 |  |
| *Exastilithoxus fimbriatus* (Steindachner, 1915) |  |  |  | D |  |  |  |  | Ferreira et al. 2007 | INPA- 15092; 38957 | Neg/Ori |
| *Exastilithoxus hoedemani* Isbrücker & Nijssen, 1985 |  |  |  |  |  |  |  | H | Reis et al. 2003 | INPA- 506 | Neg |
| *Farlowella amazonum* (Günther, 1864) |  |  |  |  |  |  |  | H | Reis et al. 2003 | NMW- 46498 | Amaz/Toc/Par |
| *Farlowella* cf. *gladiolus* (Günther, 1864) |  |  |  | D |  |  |  |  | Ferreira et al. 2007 | INPA- 8081; 8159 | Neg/Tap |
| *Farlowella* cf. *hasemani*Eigenmann & Vance, 1917 |  |  |  |  |  |  |  | H | INPA Fish Collection | INPA-57607 |  |
| *Farlowella nattereri* Steindachner, 1910 |  |  |  | D |  |  |  |  | Ferreira et al. 2007 | INPA- 1637; 16760 | Neg/Gui/upp Amaz |
| *Farlowella oxyrryncha* (Kner, 1853) |  |  |  | D |  |  |  |  | Ferreira et al. 2007 | MZUSP- 22919 | Amaz/Ori |
| *Farlowella rugosa* Boeseman, 1971 |  |  |  |  | E |  |  |  | INPA Fish Collection | INPA- 37025 | Neg/Gui |
| *Farlowella* *schreitmuelleri* Ahl, 1937 |  |  |  | D | E |  |  |  | Zuanon et al. 2015 | MZUSP- 81368 | Neg/low Amaz |
| *Farlowella smithi* Fowler, 1913 |  |  |  | D |  |  |  |  | Ferreira et al. 2007 | INPA- 16763; 27505 | Amaz/Neg |
| *Furcodontichthys novaesi* Rapp Py-Daniel, 1981 | A | B | C |  |  |  |  |  | Rapp Py-Daniel et al. 2017 | INPA- 10291; 11334 | Neg/mid Amaz |
| *Hemiancistrus* cf. *guahiborum* Werneke, Armbruster, Lujan & Taphorn, 2005 |  |  |  |  |  |  |  |  | INPA Fish Collection | INPA- 6592 | Neg/Ori |
| *Hemiancistrus* sp. |  |  |  | D |  |  |  |  | Goulding et al. 1988 | MZUSP- 35097 | Amaz/Gui |
| *Hemiancistrus subviridis*Werneke, Sabaj Pérez, Lujan & Armbruster, 2005 |  |  |  |  |  |  |  | H | INPA Fish Collection | INPA- 51148; 51149 | Neg/Ori |
| *Hemiodontichthys acipenserinus* (Kner, 1853) | A |  |  |  |  | F |  |  | Goulding et al. 1988 | INPA- 300; 2403 | Neg/Ori |
| *Hypancistrus contradens*Armbruster, Lujan & Taphorn, 2007 |  |  |  |  |  |  |  | H | INPA Fish Collection | INPA- 49592 | Neg/Ori |
| *Hypancistrus debilittera*Armbruster, Lujan & Taphorn, 2007 |  |  |  |  |  |  |  | H | INPA Fish Collection | INPA- 37579 | Neg/Ori |
| *Hypancistrus inspector* Armbruster, 2002 |  |  |  | D |  |  |  |  | Lima et al. 2005 | INPA- 42905; 42999 | Amaz/Gui |
| *Hypancistrus lunaorum*Armbruster, Lujan & Taphorn, 2007 |  |  |  |  |  |  |  | H | INPA Fish Collection | INPA- 57670 | Neg/Ori |
| *Hypancistrus phantasma* Tan & Armbruster, 2016 |  |  |  |  |  |  |  | H | Tan and Armbruster 2016 | MZUSP- 116531 | Neg |
| *Hypancistrus margaritatus* Tan & Armbruster, 2016 |  |  |  |  |  |  |  | H | Tan and Armbruster 2016 | AUM- 35610 | Neg (Bra) |
| *Hypoptopoma gulare* Cope, 1878 |  | B |  | D |  |  |  | H | INPA Fish Collection | INPA- 14029; 20780 | Amaz/Ori |
| *Hypoptopoma guianense* Boeseman, 1974 |  |  |  | D |  |  |  |  | Ferreira et al. 2007 | INPA- 7898 | Neg/Gui |
| *Hypoptopoma incognitum* Aquino & Schaefer, 2010 |  |  |  |  |  |  |  |  | INPA Fish Collection | INPA- 155; 36080 |  |
| *Hypoptopoma* sp. |  |  |  |  | E |  |  |  | Anjos and Zuanon 2007 | INPA- 25416; 36794 |  |
| *Hypoptopoma steindachneri* Boulenger, 1895 |  |  |  |  |  |  |  | H | Reis et al. 2003 | NMW- 46272 | Neg/mid Amaz |
| *Hypoptopoma thoracatum* Günther, 1868 |  | B |  | D | E |  |  |  | Ferreira et al. 2007 | INPA- 34642 | Amaz/Neg |
| *Hypostomus carinatus* (Steindachner, 1881) | A | B |  | D |  |  |  |  | Goulding et al. 1988 | INPA- 1194; 31836 | Amaz/Neg |
| *Hypostomus crassicauda*Boeseman, 1968 |  |  |  |  |  |  |  | H | INPA Fish Collection | INPA- 54782 | Neg/Gui |
| *Hypostomus hemicochliodon* Armbruster, 2003 |  |  |  |  |  |  |  | H | Armbruster 2003 | MBUCV- 19239 | Amaz/Ori |
| *Hypostomus hemiurus*(Eigenmann, 1912) |  |  |  |  |  |  |  | H | INPA Fish Collection | INPA- 33943 | Neg/Gui |
| *Hypostomus kopeyaka* Carvalho, Lima & Zawadzki, 2010 |  |  |  |  |  |  |  | H | Carvalho et al. 2010 | MZUSP- 98764 | Neg |
| *Hypostomus macushi* Armbruster & de Souza, 2005 | A |  |  | D |  |  | G | H | Carvalho et al. 2010 | INPA- 8171 | Neg/Gui |
| *Hypostomus oculeus* (Fowler, 1943) |  |  |  |  |  |  |  | H | INPA Fish Collection | INPA- 36970 |  |
| *Hypostomus plecostomus* (Linnaeus, 1758) |  |  |  | D |  |  |  |  | Ferreira et al. 2007 | INPA- 404; 423 | Neg/Ori/Gui |
| *Hypostomus sculpodon* Armbruster, 2003 |  |  |  |  |  |  |  | H | Armbruster 2003 | MCNG- 12148 | Neg/Ori |
| *Hypostomus taphorni* (Lilyestrom, 1984) |  |  |  |  |  |  |  | H | INPA Fish Collection | INPA- 6299; 6303 | Neg/Ori/Gui |
| *Hypostomus weberi* Carvalho, Lima & Zawadzki, 2010 |  |  |  |  |  |  |  | H | Carvalho et al. 2010 | MZUSP- 98767 | Neg |
| *Lamontichthys filamentosus* (La Monte, 1935) |  |  |  |  |  |  |  | H | INPA Fish Collection | INPA- 17927 | Amaz/Neg |
| *Leptotocinclus ctenistus* Delapieve, Lehmann A & Reis, 2018 |  |  |  |  |  |  |  | H | INPA Fish Collection | INPA- 53272 | Neg/Mid Amaz |
| *Lasiancistrus* *schomburgkii* (Günther, 1864) | A |  |  | D |  |  | G |  | Ferreira et al. 2007 | INPA- 1962 | Amaz/Ori/Gui |
| *Limatulichthys griseus* (Eigenmann, 1909) |  | B |  | D |  |  |  |  | Rapp Py-Daniel et al. 2017 | INPA- 25869; 36010 | Amaz/Ori |
| *Limatulichthys petleyi* (Fowler, 1940) | A |  |  | D |  | F |  |  | Ferraris-Jr 2007 | BMNH- 1893.4.24.18 | Amaz/Toc/Par |
| *Lithoxus bovallii* (Regan, 1906) |  |  |  |  |  |  |  | H | Reis et al. 2003 | BMNH- 1905.11.1.43 | Neg/Gui |
| *Loricaria cataphracta* Linnaeus, 1758 | A | B |  | D |  | F |  |  | Thomé-Souza and Chao 2004 | INPA- 14811; 14812 | Amaz/Gui |
| *Loricaria clavipinna* Fowler, 1940 |  |  |  |  |  |  |  | H | INPA Fish Collection | INPA- 37799 | Amaz/Neg |
| *Loricaria lundbergi* Thomas & Rapp Py-Daniel, 2008 |  |  |  |  |  |  |  | H | Thomas and Rapp Py-Daniel 2008 | INPA- 38100; 42784 | Neg |
| *Loricaria simillima* Regan, 1904 |  |  |  | D |  |  |  |  | Ferreira et al. 2007 | INPA- 4875; 6014 | Amaz/Ori/LaP |
| *Loricaria spinulifera* Thomas & Rapp Py-Daniel, 2008 |  |  |  |  |  |  |  | H | Thomas and Rapp Py-Daniel 2008 | INPA- 28849; 28850 | Neg |
| *Loricariichthys acutus* (Valenciennes, 1840) | A | B |  | D |  | F |  |  | Zuanon et al. 1998 | INPA- 157; 2440 | Amaz/Neg |
| *Loricariichthys nudirostris* (Kner, 1853) | A | B |  | D |  | F |  |  | Zuanon et al. 1998 | INPA- 2439; 2442 | Amaz/Neg |
| *Loricariichthys stuebelii* (Steindachner, 1882) |  |  |  |  |  |  |  | H | INPA Fish Collection | INPA- 179 | Neg/upp Amaz |
| *Nannoptopoma* sp*.* |  |  |  |  |  |  |  | H | INPA Fish Collection | INPA- 25483 |  |
| *Neblinichthys pilosus* Ferraris, Isbrücker & Nijssen, 1986 |  |  |  |  |  |  |  | H | INPA Fish Collection | INPA- 2975; 2976 | Neg/Ori |
| *Niobichthys ferrarisi* Schaefer & Provenzano, 1998 |  |  |  |  |  |  |  | H | Reis et al. 2003 | MBUCV- V-20225 | Neg |
| *Oxyropsis acutirostris* Miranda-Ribeiro, 1951 |  | B |  | D |  | F |  |  | Goulding et al. 1988 | INPA- 14325; 30431 | Neg/Ori |
| *Oxyropsis carinata* (Steindachner, 1879) |  |  |  | D | E |  |  |  | Ferreira et al. 2007 | INPA- 203; 34650 | Amaz/Neg |
| *Panaqolus claustellifer* Tan; Sousa & Armbruster, 2016 |  |  |  |  |  |  |  | H | Tan et al. 2016 | MZUSP- 33704 | Neg/Gui |
| *Panaque* sp. |  |  |  | D |  |  |  |  | Ferreira et al. 2007 | MZUSP- 33704 |  |
| *Parancistrus* sp. |  |  |  | D |  |  |  |  | Ferreira et al. 2007 | INPA- 6286 |  |
| *Pareiorhaphis regani* (Giltay, 1936) |  |  |  |  |  |  |  | H | Ferraris-Jr 2007 | IRSNB- 47 | Neg |
| *Parotocinclus britskii* Boeseman, 1974 |  |  |  |  | E |  |  |  | Ferreira et al. 2007 | INPA- 1619; 1655 | Neg/Ori/Gui |
| *Parotocinclus collinsae* Schmidt & Ferraris, 1985 |  |  |  |  |  |  |  | H | Schaefer 2003 | AMNH- 55433 | Neg (Bra) |
| *Parotocinclus eppleyi* Schaefer & Provenzano, 1993 |  |  |  |  |  |  |  | H | INPA Fish Collection | INPA- 51389; 54965 | Neg/Ori |
| *Parotocinclus longirostris* Garavello, 1988 |  |  |  |  | E |  |  |  | Zuanon et al. 2015 | INPA- 27714; 27854 | Amaz/Neg |
| *Parotocinclus polyochrus* Schaefer, 1988 |  |  |  | D |  |  |  |  | INPA Fish Collection | INPA- 15885 | Neg |
| *Parotocinclus yaka* Lehmann, Lima & Reis, 2018 |  |  |  |  |  |  |  | H | Lehmann et al. 2018 | MZUSP- 123655 | Neg |
| *Peckoltia braueri* (Eigenmann, 1912) | A |  |  | D |  |  | G | H | Armbruster 2008 | INPA- 33841; 36964 | Neg (Bra) |
| *Peckoltia sabaji* Armbruster, 2003 |  |  |  |  |  |  | G |  | Ferreira et al. 2007 | INPA- 36923; | Neg/Ori/Gui |
| *Peckoltia* sp. |  |  |  | D |  |  |  | H | INPA Fish Collection | INPA- 33843 |  |
| *Peckoltia vittata* (Steindachner, 1881) | A |  |  | D |  |  | G |  | Thomé-Souza and Chao 2004 | INPA- 4879 | Amaz/Ori |
| *Planiloricaria cryptodon* (Isbrücker, 1971) |  |  |  |  |  |  |  | H | INPA Fish Collection | INPA- 26904; 36771 | Neg/upp Amaz |
| *Pseudacanthicus histrix* (Valenciennes, 1840) |  |  |  |  |  |  |  | H | Reis et al. 2003 | INPA 1985 | Neg/low Amaz |
| *Pseudacanthicus* *leopardus* (Fowler, 1914) |  |  |  | D |  |  | G |  | Ferreira et al. 2007 | INPA- 2011 | Neg/Gui |
| *Pseudacanthicus spinosus* (Castelnau, 1855) |  |  |  |  |  |  |  |  | INPA Fish Collection | INPA- 15133 | Amaz/Neg |
| *Pseudancistrus barbatus*(Valenciennes, 1840) |  |  |  |  |  |  |  | H | INPA Fish Collection | INPA- 6144; 6206 | Neg/Gui |
| *Pseudancistrus nigrescens*Eigenmann, 1912 |  |  |  |  |  |  |  | H | INPA Fish Collection | INPA- 57672 | Neg/Gui |
| *Pseudancistrus pectegenitor* Lujan, Armbruster & Sabaj, 2007 |  |  |  |  |  |  |  | H | Lujan et al. 2007 | ANSP- 182801 | Neg/Ori |
| *Pseudancistrus sidereus* Armbruster, 2004 |  |  |  |  |  |  |  | H | Ferraris-Jr 2007 | MCNG- 26125 | Neg/Ori |
| *Pseudancistrus* sp. |  |  |  | D |  |  | G |  | Ferreira et al. 2007 | MZUSP- 43331; INPA- 1976 | |
| *Pseudohemiodon* sp. |  |  |  | D |  |  |  |  | Goulding et al. 1988 | MZUSP- 35115 |  |
| *Pseudolithoxus dumus* (Armbruster & Provenzano, 2000) |  |  |  |  |  |  |  | H | Ferraris-Jr 2007 | MBUCV- V-17544 | Neg/Ori |
| *Pseudolithoxus nicoi* (Armbruster & Provenzano, 2000) |  |  |  | D |  |  | G |  | Lima et al. 2005 | INPA- 6575; 42767 | Neg |
| *Pseudoloricaria laeviuscula* (Valenciennes, 1840) | A | B |  | D |  | F |  |  | Rapp Py-Daniel et al. 2017 | INPA- 16223; 36039 | Neg/mid Amaz |
| *Pseudorinelepis genibarbis* (Valenciennes, 1840) |  |  |  |  |  |  |  | H | Armbruster and Hardman 1999 | ZMA- 120.102 | Neg/Ori/upp Amaz |
| *Pterosturisoma microps* (Eigenmann & Allen, 1942) |  |  |  |  |  |  |  | H | INPA Fish Collection | INPA- 36793 | Neg/upp Amaz |
| *Pterygoplichthys gibbiceps* (Kner, 1854) | A | B |  | D |  |  |  | H | Reis et al. 2003 | INPA- 2434; 6579 | Neg/Ori/mid Amaz |
| *Pterygoplichthys pardalis* (Castelnau, 1855) |  |  |  |  | E |  |  |  | INPA Fish Collection | INPA- 28239; 28296 | Amaz/Neg |
| *Pterygoplichthys punctatus* Günther, 1864 |  |  |  |  |  |  |  | H | Armbruster and Page 2006 | INPA- 43818- 43822 | Amaz/Neg |
| *Reganella depressa* (Kner, 1853) | A | B | C |  |  | F |  |  | Rapp Py-Daniel et al. 2017 | INPA- 10297; 12571 | Neg/Tap |
| *Rineloricaria castroi* Isbrücker & Nijssen, 1984 | A |  |  | D |  | F |  |  | Ferreira et al. 2007 | INPA- 20005; 22123 | Amaz/Neg |
| *Rineloricaria* cf. *parva* (Boulenger, 1895) |  |  |  | D |  |  |  |  | Ferreira et al. 2007 | INPA- 8140 | Neg/Par |
| *Rineloricaria daraha* Py-Daniel & Fichberg, 2008 |  |  |  |  |  |  | G | H | Rapp Py-Daniel and Fichberg 2008 | INPA- 28579; 17939 | Neg |
| *Rineloricaria fallax* (Steindachner, 1915) | A |  |  | D |  |  |  |  | Ferreira et al. 2007 | MZUSP- 48797 | Neg/Gui |
| *Rineloricaria formosa* Isbrücker & Nijssen, 1979 |  |  |  | D |  |  |  | H | Rapp Py-Daniel and Fichberg 2008 | INPA- 6581; 16217 | Neg/Ori/upp Amaz |
| *Rineloricaria hasemani* Isbrücker & Nijssen, 1979 | A |  |  | D |  |  |  |  | Ferreira et al. 2007 | INPA- 8136; 25863 | Neg/low Amaz/Toc |
| *Rineloricaria heteroptera* Isbrücker & Nijssen, 1976 |  |  |  |  | E |  |  | H | Rapp Py-Daniel and Fichberg 2008 | INPA- 15870; 27506 | Neg/mid Amaz |
| *Rineloricaria jurupari* Londoño-Burbano & Urbano-Bonilla, 2018 |  |  |  |  |  |  |  | H | Londoño-Burbano and Urbano-Bonilla 2018 | MPUJ- 12520 | Neg |
| *Rineloricaria lanceolata* (Günther, 1868) | A |  |  | D | E |  |  | H | Rapp Py-Daniel and Fichberg 2008 | INPA- 15713; 27858 | Amaz/Gui/Par/LaP |
| *Rineloricaria melini* (Schindler, 1959) |  |  |  |  |  |  |  | H | Reis et al. 2003 | NRM- 10830 | Amaz/Neg |
| *Rineloricaria phoxocephala* (Eigenmann & Eigenmann, 1889) | A |  |  | D |  |  |  | H | Rapp Py-Daniel and Fichberg 2008 | INPA- 22074; 25864 | Amaz/Neg |
| *Rineloricaria stewarti* (Eigenmann, 1909) |  |  |  |  |  |  |  | H | Ferraris-Jr 2007 | FMNH- 53330 | Neg/Ori |
| *Spatuloricaria* sp. | A |  |  | D |  | F |  |  | Thomé-Souza and Chao 2004 | INPA- 1965; 4883 |  |
| *Sturisoma rostratum* (Spix & Agassiz, 1829) |  |  |  | D |  |  |  |  | Ferreira et al. 2007 | INPA- 16585 | Neg/mid Amaz |
| *Sturisoma* *tenuirostris* (Steindachner, 1910) |  |  |  | D |  |  |  |  | Ferreira et al. 2007 | INPA- 1992 | Neg/Ori |
| **Pimelodidae** |  |  |  |  |  |  |  |  |  |  |  |
| *Brachyplatystoma capapretum* Lundberg & Akama, 2005 |  | B |  |  |  |  |  |  | INPA Fish Collection | INPA- 5459; 26534 | Amaz/Neg |
| *Brachyplatystoma filamentosum* (Lichtenstein, 1819) | A | B | C |  |  |  |  |  | Zuanon et al. 1998 | INPA- 9628; 10302 | Amaz/Ori/Gui |
| *Brachyplatystoma juruense* (Boulenger, 1898) |  |  |  |  |  |  |  | H | INPA Fish Collection | INPA- 26903; 36762 | Amaz/Ori |
| *Brachyplatystoma platynemum* Boulenger, 1898 | A |  | C | D |  |  |  |  | Ferreira et al. 2007 | INPA- 26906; 28638 | Amaz/Ori |
| *Brachyplatystoma rousseauxii* (Castelnau, 1855) |  |  | C | D |  | F |  |  | INPA Fish Collection | INPA- 8351 | Amaz/Ori/Gui |
| *Brachyplatystoma tigrinum* (Britski, 1981) |  |  |  |  |  |  |  | H | INPA Fish Collection | INPA- 26707 | Amaz/Neg |
| *Brachyplatystoma vaillantii*(Valenciennes, 1840) |  |  |  | D |  |  |  |  | Ferraris-Jr 2007 | Syntypes: at NMW | Amaz/Ori/Gui |
| *Calophysus macropterus* (Lichtenstein, 1819) | A | B | C | D |  | F |  |  | Zuanon et al. 2008 | INPA- 15915; 35935 | Amaz/Ori |
| *Cheirocerus goeldii* (Steindachner, 1908) | A |  |  |  |  | F |  |  | Ferreira et al. 2007 | INPA- 17770; 17774 | Neg/upp Amaz |
| *Cheirocerus* cf. *eques* Eigenmann, 1917 |  |  |  | D |  |  |  |  | Goulding et al. 1988 | INPA- 36758 | Amaz/Neg |
| *Duopalatinus peruanus* Eigenmann & Allen, 1942 | A |  |  |  |  |  |  |  | Thomé-Souza and Chao 2004 | INPA- 28354; 28634 | Amaz/Ori |
| *Exallodontus aguanai* Lundberg, Mago-Leccia & Nass, 1991 |  |  |  |  |  |  |  |  | INPA Fish Collection | INPA- 26901; 36783 | Amaz/Ori |
| *Hemisorubim platyrhynchos* (Valenciennes, 1840) |  |  |  | D |  |  |  | H | Ferreira et al. 2007 | INPA- 28198; 36070 | Amaz/Ori/Gui/Arg |
| *Hypophthalmus edentatus* Spix & Agassiz, 1829 | A | B | C |  |  |  |  |  | Zuanon et al. 2008 | INPA- 18010; 30698 | Amaz/Ori/Gui |
| *Hypophthalmus fimbriatus* Kner, 1858 | A | B | C | D |  | F |  |  | Zuanon et al. 2008 | INPA- 12359; 16103 | Amaz/Ori |
| *Hypophthalmus marginatus* Valenciennes, 1840 | A | B | C | D |  |  |  |  | Zuanon et al. 2008 | INPA- 10285; 18055 | Amaz/Ori/Gui |
| *Leiarius marmoratus* (Gill, 1870) |  |  |  | D |  |  |  |  | Goulding et al. 1988 | INPA- 7751; 16095 | Amaz/Ori/Gui |
| *Megalonema amaxanthum* Lundberg & Dahdul, 2008 |  |  |  |  |  |  |  | H | INPA Fish Collection | INPA- 29490; 29491 | Amaz/Gui |
| *Megalonema platycephalum* Eigenmann, 1912 |  |  |  | D |  |  |  |  | Ferreira et al. 2007 | INPA- 36318; 43025 | Amaz/Ori/Gui |
| *Phractocephalus hemioliopterus* (Bloch & Schneider, 1801) |  | B |  | D |  |  |  |  | Goulding et al. 1988 | INPA- 2155; 35963 | Amaz/Ori/Gui |
| *Pimelodidae* sp. 1 |  |  |  | D |  |  |  |  | Ferreira et al. 2007 | MZUSP- 55536 |  |
| *Pimelodidae* sp. 2 'orcesi' |  |  |  | D |  |  |  |  | Ferreira et al. 2007 | MZUSP- 55537 |  |
| *Pimelodina flavipinnis* Steindachner, 1876 | A | B | C | D |  |  |  |  | Thomé-Souza and Chao 2004 | INPA- 11339; 18021 | Amaz/Ori |
| *Pimelodus albofasciatus* Mees, 1974 |  |  |  | D |  | F |  |  | Lima et al. 2005 | INPA- 3177; 16737 | Amaz/Ori/Gui |
| *Pimelodus altissimus* Eigenmann & Pearson, 1942 | A |  |  |  |  |  |  |  | Thomé-Souza and Chao 2004 | INPA- 4452; 4454 | Amaz/Neg |
| *Pimelodus blochii* Valenciennes, 1840 | A | B | C | D |  | F |  |  | Zuanon et al. 2008 | INPA- 10275; 12343 | Amaz/Ori/Gui |
| *Pimelodus* cf. *tetramerus* Ribeiro & Lucena, 2006 |  |  |  |  |  |  |  | H | INPA Fish Collection | INPA- 36865 | Neg/Tap/Toc |
| *Pimelodus microstoma* Steindachner, 1876 |  |  |  | D |  |  |  |  | Ferraris-Jr 2007 | NMW- 45823 | Neg |
| *Pimelodus ornatus* Kner, 1858 |  |  |  | D |  |  |  |  | Lima et al. 2005 | INPA- 6590; 11101 | Neg/mid Amaz |
| *Pinirampus pirinampu* (Spix & Agassiz, 1829) | A | B | C | D |  |  |  |  | Zuanon et al. 2008 | INPA- 10278; 16100 | Amaz/Ori/Gui/Par |
| *Platynematichthys notatus* (Jardine, 1841) |  | B |  | D |  |  |  |  | Goulding et al. 1988 | INPA- 36190 | Amaz/Ori |
| *Platysilurus mucosus* (Vaillant, 1880) |  |  |  | D |  |  |  |  | Ferreira et al. 2007 | INPA- 26914 | Amaz/Ori |
| *Platystomatichthys sturio* (Kner, 1858) | A |  | C | D |  |  |  |  | Thomé-Souza and Chao 2004 | INPA- 26920; 28631 | Amaz/Neg |
| *Propimelodus caesius* Parisi, Lundberg & Do Nascimiento, 2006 | A |  |  |  |  |  |  | H | Parisi et al. 2006 | INPA- 26473; 30014 | Amaz/Neg |
| *Propimelodus eigenmanni* (Van der Stigchel, 1946) | A |  |  |  |  |  |  | H | INPA Fish Collection | INPA- 36770; 36792 | Amaz/Gui |
| *Propimelodus* sp. |  |  |  |  |  |  |  | H | INPA Fish Collection | INPA- 28644; 36802 |  |
| *Pseudoplatystoma punctifer* (Castelnau, 1855) | A | B |  | D |  |  |  | H | Buitrago–Suárez and Burr 2007 | INPA- 42279 | Amaz/Neg |
| *Pseudoplatystoma reticulatum* Eigenmann & Eigenmann, 1889 |  |  |  |  |  |  |  | H | Buitrago–Suárez and Burr 2007 | MCZ- 23813 | Neg/mid Amaz/Par |
| *Pseudoplatystoma tigrinum* (Valenciennes, 1840) |  | B |  | D |  |  |  | H | Buitrago–Suárez and Burr 2007 | CAS- 6379 | Amaz/Neg |
| *Sorubim elongatus* Littmann, Burr, Schmidt & Isern, 2001 |  |  |  | D |  | F |  | H | Ferreira et al. 2007 | INPA- 25764; 35556 | Amaz/Ori/Gui |
| *Sorubim lima* (Bloch & Schneider, 1801) |  | B |  | D |  |  |  |  | Saint-Paul et al. 2000 | INPA- 805; 25763 | Neg/mid Amaz/Par |
| *Sorubim maniradii* Littmann, Burr & Buitrago-Suarez, 2001 |  |  |  |  |  |  |  | H | INPA Fish Collection | INPA- 26634 | Amaz/Neg |
| *Sorubimichthys planiceps* (Spix & Agassiz, 1829) |  |  |  | D |  |  |  |  | Reis et al. 2003 |  | Neg/mid Amaz/Par/Arg |
| *Zungaro zungaro* (Humboldt, 1821) |  |  |  | D |  |  |  |  | Zuanon et al. 2008 | INPA- 1629; 42284 | Amaz/Ori/Gui |
| **Phreatobiidae** |  |  |  |  |  |  |  |  |  |  |  |
| *Phreatobius* sp. 1 'tarumã-mirim' |  |  |  | D |  |  |  | H | INPA Fish Collection | INPA- 26240; 26244; 26247 | Neg |
| *Phreatobius* sp. 2 'são gabriel da cachoeira' |  |  |  | D |  |  |  | H | INPA Fish Collection | INPA- 49570; 42888; 42988 | Neg |
| *Phreatobius* sp. 3 'anapixi' |  |  |  |  |  |  |  | H | INPA Fish Collection | INPA- 16696; 25505 | Neg |
| *Phreatobius* sp. 4 'barcelos' |  |  |  |  |  |  |  | H | INPA Fish Collection | INPA- 14762; 28629; 28630 | Neg |
| **Pseudopimelodidae** |  |  |  |  |  |  |  |  |  |  |  |
| *Batrochoglanis raninus* (Valenciennes, 1840) |  |  |  | D | E |  |  |  | Zuanon et al. 2015 | INPA- 6589; 19863 | Amaz/Gui |
| *Batrochoglanis* *villosus* (Eigenmann, 1912) |  |  |  | D | E |  |  |  | Ferreira et al. 2007 | INPA- 35222; 35223 | Amaz/Ori/Gui |
| *Microglanis* cf. *oliveirai*Ruiz & Shibatta, 2011 |  |  |  |  |  |  |  | H | INPA Fish Collection | INPA- 54903; 54993 | Toc/Neg |
| *Microglanis poecilus* Eigenmann, 1912 |  |  |  |  | E |  | G |  | INPA Fish Collection | INPA- 34600; 37023 | Amaz/Ori/Gui |
| *Microglanis* *secundus* Mees, 1974 |  |  |  |  |  |  |  |  | INPA Fish Collection | INPA- 7907; 11837 | Neg/Ori/Gui |
| *Pseudopimelodus bufonius* (Valenciennes, 1840) | A |  |  |  |  |  | G |  | Ferreira et al. 2007 | INPA- 10954; 11057 | Amaz/Ori/Gui |
| *Rhyacoglanis* aff. *pulcher* (Boulenger, 1887) |  |  |  |  |  |  |  | H | INPA Fish Collection | INPA- 34986 | Neg/upp Amaz |
| **Scoloplacidae** |  |  |  |  |  |  |  |  |  |  |  |
| *Scoloplax baileyi* Rocha, Lazzorotto & Rapp Py-Daniel, 2012 |  |  |  |  |  |  |  | H | Rocha et al. 2012 | INPA 35637 | Neg |
| *Scoloplax baskini* Rocha, de Oliveira & Rapp Py-Daniel, 2008 |  |  |  |  |  |  |  | H | Rocha et al. 2012 | INPA- 36327; 39963 | Neg/mid Amaz |
| *Scoloplax dicra* Bailey & Baskin, 1976 |  |  |  | D | E |  |  |  | Rocha et al. 2012 | INPA- 36097 | Amaz/Par |
| *Scoloplax dolicholophia* Schaefer, Weitzman & Britski, 1989 |  |  |  | D |  |  |  | H | Rocha et al. 2008a | INPA- 26257; 28955 | Neg |
| **Trichomycteridae** |  |  |  |  |  |  |  |  |  |  |  |
| *Acanthopoma* sp. |  |  |  | D |  |  |  |  | Ferreira et al. 2007 | INPA- 8158 |  |
| *Ammoglanis* sp. |  |  |  |  |  |  |  | H | INPA Fish Collection | INPA- 42854; 49784 |  |
| *Apomatoceros alleni*Eigenmann, 1922 |  |  |  |  |  |  |  | H | INPA Fish Collection | INPA-25849 | Up Amaz/Neg |
| *Glanapteryx anguilla* Myers, 1927 |  |  |  | D |  |  |  | H | INPA Fish Collection | INPA- 14258; 29457 | Neg/Ori |
| *Glanapteryx niobium* de Pinna, 1998 |  |  |  |  |  |  |  | H | INPA Fish Collection | INPA- 12421 | Neg |
| *Haemomaster venezuelae* Myers, 1927 |  |  |  | D |  | F |  |  | Ferreira et al. 2007 | INPA- 36876; | Amaz/Ori |
| *Henonemus punctatus* (Boulenger, 1887) |  |  |  |  |  |  |  | H | INPA Fish Collection | INPA- 16583; 36877 | Neg/upp Amaz |
| *Ituglanis amazonicus* (Steindachner, 1882) |  |  |  |  | E |  |  |  | Zuanon et al. 2015 | INPA- 13223; 32864 | Amaz/Gui |
| *Ituglanis gracilior* (Eigenmann, 1912) |  |  |  |  |  |  |  |  | INPA Fish Collection | INPA- 38863 | Neg/Ori/Gui |
| *Megalocentor echthrus* de Pinna & Britski, 1991 |  |  |  | D |  | F |  |  | Ferreira et al. 2007 | INPA- 36755 | Amaz/Ori |
| *Ochmacanthus alternus* Myers, 1927 |  |  |  |  | E | F |  | H | Reis et al. 2003 | CAS- 13522 | Neg/Ori |
| *Ochmacanthus orinoco* Myers, 1927 |  |  |  | D |  |  |  |  | Goulding et al. 1988 | INPA- 24453 | Neg/Ori |
| *Ochmacanthus reinhardtii*(Steindachner, 1882) |  |  |  |  |  |  |  | H | INPA Fish Collection | INPA- 48796 | Amaz/Gui |
| *Paracanthopoma parva* Giltay, 1935 |  |  |  |  | E | F |  |  | Ferreira et al. 2007 | INPA- 29899 | Amaz/Gui |
| *Paravandellia* sp. |  |  |  |  |  |  |  | H | INPA Fish Collection | INPA- 37364;39363 |  |
| *Pareiodon microps* Kner, 1855 |  |  |  |  |  |  |  | H | INPA Fish Collection | INPA- 25851 | Amaz/Neg |
| *Plectrochilus* sp. |  |  |  |  |  |  |  | H | INPA Fish Collection | INPA- 20804 |  |
| *Potamoglanis hasemani* (Eigenmann, 1914) |  |  |  |  | E | F |  |  | INPA Fish Collection | INPA- 16717; 30059 | Amaz/Gui |
| *Potamoglanis wapixana* Henschel, 2016 |  |  |  |  |  |  |  | H | Henschel et al. 2018 | UFRJ- 10251 | Neg (Bra) |
| *Pseudostegophilus nemurus* (Günther, 1869) |  |  |  |  |  |  |  | H | INPA Fish Collection | INPA- 16714; 25848 | Amaz/Ori |
| *Pygidianops amphioxus* de Pinna & Kirovski, 2011 |  |  |  |  | E | F |  |  | de Pinna and Kirovski 2011 | INPA- 27856 | Neg |
| *Pygidianops eigenmanni* Myers, 1944 |  |  |  |  |  |  |  | H | INPA Fish Collection | INPA- 8080 | Neg |
| *Sarcoglanis simplex* Myers & Weitzman, 1966 |  |  |  | D |  | F |  | H | Reis et al. 2003 | INPA- 9043; 36430 | Neg |
| *Stauroglanis gouldingi* de Pinna, 1989 |  |  |  | D | E | F |  |  | Lima et al. 2005 | INPA- 14979 | Neg |
| *Stegophilus panzeri*(Ahl, 1931) |  |  |  |  |  |  |  | H | INPA Fish Collection | INPA- 51960 | Low Amaz/Neg |
| *Trichomycterus gabrieli* (Myers, 1926) |  |  |  | D |  |  |  | H | Reis et al. 2003 | INPA- 12987 | Neg |
| *Trichomycterus lewi* Lasso & Provenzano, 2003 |  |  |  |  |  |  |  | H | INPA Fish Collection | INPA- 38958 | Neg/Ori |
| *Typhlobelus ternetzi* Myers, 1944 |  |  |  |  |  |  |  | H | Buckup et al. 2007 | CAS- 11119 | Neg |
| *Typhlobelus* sp. |  |  |  | D |  | F |  |  | Ferreira et al. 2007 | INPA- 36431; 37005 | Neg(Bra) |
| *Vandellia cirrhosa* Valenciennes, 1846 |  |  |  | D | E | F |  |  | Goulding et al. 1988 | INPA- 25850; 36864 | Amaz/Neg |
| *Vandellia sanguinea* Eigenmann, 1917 |  |  |  | D |  | F |  |  | Ferreira et al. 2007 | INPA- 22486; 36871 | Amaz/Ori |
| **GYMNOTIFORMES** |  |  |  |  |  |  |  |  |  |  |  |
| **Apteronotidae** |  |  |  |  |  |  |  |  |  |  |  |
| *Adontosternarchus balaenops* (Cope, 1878) |  |  |  |  |  |  |  | H | INPA Fish Collection | INPA- 26281; 26627 | Neg/upp Amaz |
| *Adontosternarchus clarkae* Mago-Leccia, Lundberg & Baskin, 1985 | A |  | C |  |  |  |  |  | Thomé-Souza and Chao 2004 | INPA- 17972; 18011 | Neg/upp Amaz/Ori |
| *Adontosternarchus nebulosus* Lundberg & Cox Fernandes, 2007 |  |  |  |  |  |  |  | H | Lundberg and Cox-Fernandez 2007 | INPA- 36772 | Amaz/Neg |
| *Adontosternarchus sachsi* (Peters, 1877) | A |  | C |  |  |  |  |  | Thomé-Souza and Chao 2004 | INPA- 12366 | Neg/Ori |
| *Apteronotus albifrons* (Linnaeus, 1766) |  | B |  | D | E |  |  |  | Goulding et al. 1988 | INPA- 36034; 16150 | Amaz/Ori/Gui/Par |
| *Apteronotus apurensis*Fernández-Yépez, 1968 |  |  |  |  |  |  |  | H | INPA Fish Collection | INPA-7688; 9578 | Neg/Ori |
| *Apteronotus bonapartii* (Castelnau, 1855) | A |  | C | D |  |  |  |  | Thomé-Souza and Chao 2004 | INPA- 12627; 28868 | Amaz/Neg |
| *Apteronotus macrolepis* (Steindachner, 1881) | A |  |  |  |  |  |  |  | Buckup et al. 2007 | NMW- 65333 | Amaz/Neg |
| *Compsaraia compsus* (Mago-Leccia, 1994) |  |  |  |  |  |  |  | H | Reis et al. 2003 | INPA- 27586 | Neg/Ori |
| *Compsaraia iara* Bernt & Albert, 2017 |  |  |  |  |  |  |  | H | Bernt and Albert 2017 | ANSP- 192777; 192778 | Amaz/Neg |
| *Compsaraia samueli* Albert & Crampton, 2009 |  |  |  |  |  |  |  | H | Bernt et al. 2018 | ANSP- 189212 | Amaz/Neg |
| *Melanosternarchus amaru* Bernt; Crampton; Orfinger & Albert, 2018 |  |  |  |  |  |  |  | H | Bernt et al. 2018 | ANSP- 192675 | Amaz/Neg |
| *Orthosternarchus tamandua* (Boulenger, 1898) | A |  |  |  |  |  |  |  | Thomé-Souza and Chao 2004 | INPA- 12621; 27604 | Amaz/Neg |
| *Parapteronotus hasemani*(Ellis, 1913) |  |  |  |  |  |  |  | H | INPA Fish Collection | INPA- 14533; 16262 | Amaz/Neg |
| *Platyurosternarchus crypticus* de Santana & Vari, 2009 |  |  |  |  |  |  |  | H | de Santana and Vari 2009 | INPA- 7420 | Neg (Bra) |
| *Platyurosternarchus macrostoma* (Günther, 1870) |  |  |  | D | E |  | G |  | Ferreira et al. 2007 | INPA- 14536; 20162 | Amaz/Ori/Gui |
| *Porotergus duende* de Santana, Crampton, 2010 |  |  |  |  |  |  |  | H | Ferraris-Jr et al. 2017 | MCP 37357 | Amaz/Neg |
| *Porotergus gimbeli* Ellis, 1912 |  |  |  |  |  |  |  | H | INPA Fish Collection | INPA- 20151; 26966 | Amaz/Neg |
| *Sternarchella calhamazon* Lundberg, Cox Fernandes & Campos-Da-Paz, 2013 |  |  |  |  |  |  |  | H | Evans et al. 2017 | INPA- 13541 | Amaz/Neg |
| *Sternarchella duccis* Lundberg, Cox Fernandes & Albert, 1996 | A |  |  | D |  |  |  | H | Ivanyisky and Albert 2014 | INPA- 10445; 12577 | Amaz/Neg |
| *Sternarchella orthos* Mago-Leccia, 1994 | A |  | C |  |  |  |  |  | INPA Fish Collection | INPA- 10260; 12377 | Amaz/Ori |
| *Sternarchella raptor* (Lundberg, Cox Fernandes & Albert, 1996) |  |  |  |  |  |  |  | H | INPA Fish Collection | INPA- 10440; 11329 | Amaz/Neg |
| *Sternarchella schotti* (Steindachner, 1868) | A |  |  | D |  |  |  |  | Thomé-Souza and Chao 2004 | INPA- 16125; 17639 | Amaz/Neg |
| *Sternarchella sima* Starks, 1913 |  |  |  |  |  |  |  | H | INPA Fish Collection | INPA- 26573 | Amaz/Neg |
| *Sternarchogiton labiatus* de Santana, Crampton, 2007 |  |  |  |  |  |  |  | H | Ferraris-Jr et al. 2017 | MCP- 37544 | Amaz/Neg |
| *Sternarchogiton nattereri* (Steindachner, 1868) | A |  | C |  |  |  |  |  | Thomé-Souza and Chao 2004 | INPA- 11332; 12497 | Amaz/Ori |
| *Sternarchogiton porcinum* Eigenmann & Allen, 1942 | A |  | C | D |  |  |  |  | Thomé-Souza and Chao 2004 | INPA- 36778 | Amaz/Ori |
| *Sternarchogiton preto* de Santana & Crampton, 2007 |  |  |  |  |  |  |  | H | INPA Fish Collection | INPA- 14433; 16092 | Amaz/Ori |
| *Sternarchorhamphus muelleri* (Steindachner, 1881) | A |  | C | D |  |  |  |  | Thomé-Souza and Chao 2004 | INPA- 11330; 11457 | Amaz/Ori |
| *Sternarchorhynchus caboclo* de Santana & Nogueira, 2006 |  |  |  |  |  |  |  | H | de Santana and Nogueira 2006 | INPA- 10594; 22894 | Neg (Bra) |
| *Sternarchorhynchus* cf. *gnomus* de Santana & Taphorn, 2006 |  |  |  |  |  |  |  | H | INPA Fish Collection | INPA- 10579 | Neg/Ori |
| *Sternarchorhynchus cramptoni* de Santana & Vari, 2010 |  |  |  |  |  |  |  | H | INPA Fish Collection | INPA- 36521 | Amaz/Neg |
| *Sternarchorhynchus curvirostris* (Boulenger, 1887) | A |  | C |  |  |  |  |  | Thomé-Souza and Chao 2004 | INPA- 11704; 17605 | Neg/upp Amaz |
| *Sternarchorhynchus goeldii* de Santana & Vari, 2010 |  | B |  |  |  |  |  |  | de Santana and Vari 2010 | INPA- 11355 | Neg/upp Amaz |
| *Sternarchorhynchus mareikeae* de Santana & Vari, 2010 |  |  |  |  |  |  |  | H | INPA Fish Collection | INPA- 28861 | Tro/Neg |
| *Sternarchorhynchus montanus*de Santana & Vari, 2010 |  |  |  |  |  |  |  | H | INPA Fish Collection | INPA- 36086 | Neg/upp Amaz |
| *Sternarchorhynchus mormyrus* (Steindachner, 1868) |  |  |  | D |  |  |  |  | Goulding et al. 1988 | INPA- 17608; 17611 | Amaz/Ori |
| *Sternarchorhynchus oxyrhynchus* (Müller & Troschel, 1849) | A |  | C | D |  |  |  |  | Goulding et al. 1988 | INPA- 10578; 11355 | Neg/Ori |
| *Sternarchorhynchus retzeri* de Santana & Vari, 2010 |  |  |  |  |  |  |  | H | de Santana and Vari 2010 | INPA- 27493 | Neg/Tef |
| *Sternarchorhynchus roseni* Mago-Leccia, 1994 |  |  |  |  |  |  |  | H | INPA Fish Collection | INPA- 36782 | Neg/Ori |
| *Sternarchorhynchus severii* Santana & Nogueira, 2006 |  |  |  |  |  |  |  | H | de Santana and Nogueira 2006 | INPA- 22893; 22898 | Neg (Bra) |
| **Gymnotidae** |  |  |  |  |  |  |  |  |  |  |  |
| *Electrophorus electricus* (Linnaeus, 1766) |  | B |  | D | E |  |  |  | Zuanon et al. 2015 | INPA- 2567; 32350 | Amaz/Ori/Gui |
| *Gymnotus anguillaris* Hoedeman, 1962 |  | B |  | D | E |  |  |  | Zuanon et al. 2008 | INPA- 14258; 29457 | Amaz/Ori/Gui |
| *Gymnotus carapo* Linnaeus, 1758 |  | B |  | D | E |  |  |  | Ferreira et al. 2007 | INPA- 10376; 12716 | Amaz/Ori/Gui |
| *Gymnotus cataniapo* Mago-Leccia, 1994 |  |  |  | D | E |  |  |  | Zuanon et al. 2015 | INPA- 36960; 39292 | Neg/Ori/Gui |
| *Gymnotus coatesi*La Monte, 1935 |  |  |  |  |  |  |  | H | INPA Fish Collection | INPA- 51250 | Amaz/Neg |
| *Gymnotus coropinae* Hoedeman, 1962 |  |  |  | D | E |  |  |  | Lima et al. 2005 | INPA- 29369; 36407 | Neg/Ori/Gui |
| *Gymnotus mamiraua*Albert & Crampton, 2001 |  |  |  |  |  |  |  | H | INPA Fish Collection | INPA- 46968 | Amaz |
| *Gymnotus pedanopterus* Mago-Leccia, 1994 |  |  |  | D | E |  |  |  | Zuanon et al. 2015 | INPA- 15907; 39412 | Neg/Ori |
| *Gymnotus* *stenoleucus* Mago-Leccia, 1994 |  |  |  |  | E |  |  |  | INPA Fish Collection | INPA- 21563; 27899 | Neg/Ori |
| *Gymnotus* sp. |  |  |  |  |  |  |  | H | Zuanon et al. 2015 | INPA- 13427; 14210 | Neg |
| *Gymnotus tiquie* Maxime, Lima & Albert, 2011 |  |  |  |  |  |  |  | H | Maxime et al. 2011 | MZUSP- 104507 | Neg |
| **Hypopomidae** |  |  |  |  |  |  |  |  |  |  |  |
| *Brachyhypopomus batesi* Crampton, de Santana, Waddell, Lovejoy, 2016 |  |  |  |  |  |  |  | H | Ferraris-Jr et al. 2017 | MCP- 47020 | Amaz/Neg |
| *Brachyhypopomus beebei* (Schultz, 1944) |  |  |  | D |  |  |  |  | Zuanon et al. 2008 | INPA- 28351; 30748 | Amaz/Ori/Gui |
| *Brachyhypopomus brevirostris* (Steindachner, 1868) |  | B |  | D | E |  |  |  | Zuanon et al. 1998 | INPA- 12265; 19936 | Amaz/Ori/Gui |
| *Brachyhypopomus bullocki* Sullivan, 2009 |  |  |  |  |  |  |  | H | Sullivan 2009 | INPA- 39376 | Amaz/Ori/Gui |
| *Brachyhypopomus diazae* (Fernández-Yépez, 1972) |  |  |  |  |  |  |  | H | INPA Fish Collection | INPA- 39375; 39399 | Neg/Ori |
| *Brachyhypopomus hamiltoni* Crampton, de Santana, Waddell, Lovejoy, 2016 |  |  |  |  |  |  |  | H | Ferraris-Jr et al. 2017 | MCP- 45482 | Amaz/Neg |
| *Brachyhypopomus hendersoni* Crampton, de Santana, Waddell, Lovejoy, 2016 |  |  |  |  |  |  |  | H | Ferraris-Jr et al. 2017 | MCP- 45305 | Amaz/Gui |
| *Brachyhypopomus pinnicaudatus* (Hopkins, 1991) |  |  |  |  |  |  |  | H | INPA Fish Collection | INPA- 4389; 26282 | Amaz/Gui/Par |
| *Brachyhypopomus provenzanoi* Crampton, de Santana, Waddell, Lovejoy, 2016 |  |  |  |  |  |  |  | H | Ferraris-Jr et al. 2017 | MBUCV-V 35650 | Neg/Ori |
| *Brachyhypopomus walteri* Sullivan, Zuanon & Cox Fernandes, 2013 |  |  |  |  |  |  |  | H | Sullivan et al. 2013 | INPA- 8941 | Amaz/Gui/Par |
| *Microsternarchus bilineatus* Fernández-Yépez, 1968 |  |  |  | D | E | F |  |  | Cox-Fernandes et al. 2015 | INPA- 26270; 27596 | Neg/Ori |
| *Microsternarchus brevis* Cox-Fernandes; Nogueira; Williston & Alves-Gomes, 2015 |  |  |  |  |  |  |  | H | Cox-Fernandes et al. 2015 | INPA- 42262; 48028 | Neg |
| *Procerusternarchus pixuna* Cox-Fernandes; Nogueira & Alves-Gomes, 2014 |  |  |  |  |  |  |  | H | Cox-Fernandes et al. 2014 | INPA- 28589; 29495 | Neg |
| *Racenisia fimbriipinna* Mago-Leccia, 1994 |  |  |  |  |  |  |  | H | Reis et al. 2003 | MZUSP- 56536.0 | Neg/Ori |
| **Rhamphichthyidae** |  |  |  |  |  |  |  |  |  |  |  |
| *Gymnorhamphichthys bogardusae* Lundberg, 2005 |  |  |  |  |  |  |  |  | INPA Fish Collection | INPA- 28914; 28917 | Neg/Ori |
| *Gymnorhamphichthys hypostomus* Ellis, 1912 | A |  |  | D | E | F |  |  | INPA Fish Collection | INPA- 12557; 27624 | Amaz/Ori |
| *Gymnorhamphichthys rondoni* (Miranda Ribeiro, 1920) | A |  |  | D | E | F |  |  | INPA Fish Collection | INPA- 11687; 14209 | Amaz/Ori/Gui/Par |
| *Gymnorhamphichthys rosamariae* Schwassmann, 1989 |  |  |  |  |  |  |  | H | INPA Fish Collection | INPA- 28903; 37802 | Neg/Xin/Tap/Gui/Ori |
| *Hypopygus cryptogenes* (Triques, 1997) |  |  |  | D | E |  |  | H | de Santana and Crampton 2011 | INPA- 4386; 26254 | Neg |
| *Hypopygus hoedemani* De Santana & Crampton, 2011 |  |  |  |  |  |  |  | H | de Santana and Crampton 2011 | MZUSP- 30169; 62774 | Neg |
| *Hypopygus lepturus* Hoedeman, 1962 |  |  |  | D | E |  |  |  | Ferreira et al. 2007 | INPA- 1646; 16027 | Amaz/Ori/Gui/Par |
| *Hypopygus minissimus* De Santana & Crampton, 2011 |  |  |  |  |  |  |  |  | INPA Fish Collection | INPA- 42864 | Neg/Ori |
| *Hypopygus neblinae* Mago-Leccia, 1994 |  |  |  |  |  |  |  | H | Reis et al. 2003 | INPA- 26254; 29474 | Amaz/Ori |
| *Iracema caiana* Triques, 1996 |  |  |  |  |  |  |  | H | Reis et al. 2003 | MZUSP- 8952 | Neg |
| *Rhamphichthys drepanium* Triques, 1999 |  |  |  |  |  |  |  | H | Reis et al. 2003 | INPA- 17650; 17682 | Amaz/Ori |
| *Rhamphichthys heleios* Carvalho & Albert, 2015 |  |  |  |  |  |  |  | H | Ferraris-Jr et al. 2017 | INPA- 42309 | Amaz/Neg |
| *Rhamphichthys lineatus* Castelnau, 1855 |  |  |  |  |  |  |  | H | INPA Fish Collection | INPA- 26575; 36801 | Amaz/Neg |
| *Rhamphichthys marmoratus* Castelnau, 1855 | A |  |  |  |  |  |  |  | Thomé-Souza and Chao 2004 | INPA- 17646; 27618 | Amaz/Ori/Toc |
| *Rhamphichthys rostratus* (Linnaeus, 1766) |  | B |  | D |  | F |  |  | Ferreira et al. 2007 | INPA- 17644; 27620 | Amaz/Ori |
| *Steatogenys duidae* (La Monte, 1929) |  |  |  |  | E |  |  |  | Zuanon et al. 2015 | INPA- 14291; 16604 | Amaz/Ori |
| *Steatogenys elegans* (Steindachner, 1880) | A | B | C | D |  | F |  |  | Zuanon et al. 1998 | INPA- 11340; 11408 | Amaz/Ori |
| *Steatogenys ocellatus* Crampton, Thorsen & Albert, 2004 |  |  |  | D |  |  |  | H | Ferreira et al. 2007 | INPA- 30746 | Neg/upp Amaz |
| **Sternopygidae** |  |  |  |  |  |  |  |  |  |  |  |
| *Archolaemus blax* Korringa, 1970 |  |  |  | D |  |  |  |  | Ferreira et al. 2007 | INPA- 6499; 20956 | Neg/Gui/Toc |
| *Archolaemus ferreirai* Vari, de Santana & Wosiacki, 2012 |  |  |  |  |  |  |  | H | INPA Fish Collection | INPA- 36379; 3757 | Neg (Bra) |
| *Distocyclus conirostris* (Eigenmann & Allen, 1942) | A |  | C | D |  |  |  |  | Goulding et al. 1988 | INPA- 10295; 11341 | Amaz/Ori |
| *Distocyclus goajira* Schultz, 1949 |  |  |  | D |  |  |  |  | Goulding et al. 1988 | INPA- 19295; 11341 | Neg/Ori |
| *Eigenmannia humboldtii* (Steindachner, 1878) | A |  | C | D |  |  |  |  | Zuanon et al. 1998 | MZUSP- 29953 | Amaz/Ori |
| *Eigenmannia limbata* (Schreiner & Miranda Ribeiro, 1903) | A | B |  |  |  |  |  |  | Zuanon et al. 2008 | INPA- 16112; 20159 | Amaz/Ori/Gui |
| *Eigenmannia macrops* (Boulenger, 1897) | A | B | C | D | E | F |  |  | Zuanon et al. 2015 | INPA- 30756; 33023 | Amaz/Ori/Gui |
| *Eigenmannia matintapereira* Peixoto; Dutra & Wosiacki, 2015 |  |  |  |  |  |  |  | H | Peixoto et al. 2015 | MZUSP- 109618 | Neg |
| *Eigenmannia nigra* Mago-Leccia, 1994 |  |  |  |  |  |  |  | H | Peixoto et al. 2015 | AMNH- 58642 | Amaz/Ori/Gui |
| *Eigenmannia trilineata* López & Castello, 1966 | A | B |  | D | E | F |  |  | Anjos and Zuanon 2007 | INPA- 30020; 37801 | Amaz/Par/LaP |
| *Eigenmannia virescens* (Valenciennes, 1836) | A |  |  | D |  | F |  |  | Thomé-Souza and Chao 2004 | INPA- 17554; 33028 | Amaz/Ori/Gui/Par |
| *Rhabdolichops caviceps* (Fernández-Yépez, 1968) | A | B | C |  |  |  |  |  | Thomé-Souza and Chao 2004 | INPA- 10272; 17563 | Amaz/Ori |
| *Rhabdolichops eastwardi* Lundberg & Mago-Leccia, 1986 | A |  | C | D |  |  |  |  | Lima et al. 2005 | INPA- 10270; 11356 | Amaz/Ori |
| *Rhabdolichops electrogrammus* Lundberg & Mago-Leccia, 1986 | A |  |  | D |  |  |  |  | Thomé-Souza and Chao 2004 | INPA- 11342; 15005 | Neg/Ori |
| *Rhabdolichops lundbergi* Correa, Crampton & Albert, 2006 |  |  |  |  |  |  |  | H | INPA Fish Collection | INPA- 10310; 12687 | Amaz/Neg |
| *Rhabdolichops nigrimans* Correa, Crampton & Albert, 2006 |  |  |  |  |  |  |  | H | INPA Fish Collection | INPA- 10258; 18014 | Neg/Tef |
| *Rhabdolichops troscheli* (Kaup, 1856) | A | B | C | D |  | F |  |  | Goulding et al. 1988 | INPA- 11310; 12363 | Amaz/Ori |
| *Sternopygus astrabes* Mago-Leccia, 1994 |  |  |  |  |  |  |  | H | Reis et al. 2003 | INPA- 13236; 30502 | Neg/Ori |
| *Sternopygus branco* Crampton; Hulen & Albert, 2004 |  |  |  |  |  |  |  | H | Crampton et. al. 2004 | INPA- 26556 | Neg |
| *Sternopygus macrurus* (Bloch & Schneider, 1801) | A | B | C | D | E |  |  |  | Lima et al. 2005 | INPA- 12370; 17619 | Amaz/Ori/Gui/Par |
| *Sternopygus obtusirostris* Steindachner, 1881 |  |  |  |  |  |  |  | H | Reis et al. 2003 | INPA- 9072 | Neg/mid Amazon |
| **EUTELEOSTOMORPHA** |  |  |  |  |  |  |  |  |  |  |  |
| **BATRACHOIDIARIA** |  |  |  |  |  |  |  |  |  |  |  |
| **BATRACHOIDIFORMES** |  |  |  |  |  |  |  |  |  |  |  |
| **Batrachoididae** |  |  |  |  |  |  |  |  |  |  |  |
| *Thalassophryne amazonica* Steindachner, 1876 |  |  |  |  |  |  |  | H | Reis et al. 2003 | NMW (not found in 1995) | Amaz/Neg |
| **GOBIARIA** |  |  |  |  |  |  |  |  |  |  |  |
| **GOBIIFORMES** |  |  |  |  |  |  |  |  |  |  |  |
| **Eleotridae** |  |  |  |  |  |  |  |  |  |  |  |
| *Leptophilypnion fittkaui* Robert, 2013 |  |  |  |  |  |  |  | H | Roberts 2013 | INPA- 40977 | Neg |
| *Microphilypnus hypolyrasimeion* Caires & Toledo-Piza, 2018 |  |  |  |  |  |  |  | H | Caires and Toledo-Piza 2018 | MZUSP- 121679; 95404 | Neg |
| *Microphilypnus macrostoma* Myers, 1927 |  |  |  |  | E | F |  |  | INPA Fish Collection | INPA- 11761 | Neg/Ori |
| *Microphilypnus ternetzi* Myers, 1927 |  |  |  |  |  |  |  | H | INPA Fish Collection | INPA- 26253; 32784 | Amaz/Ori |
| **ANABANTARIA** |  |  |  |  |  |  |  |  |  |  |  |
| **ANABANTIFORMES** |  |  |  |  |  |  |  |  |  |  |  |
| **Osphronemidae (Non-native)** |  |  |  |  |  |  |  |  |  |  |  |
| *Trichopodus trichopterus* (Pallas, 1770) |  |  |  |  | E |  |  |  | UFAM Fish Collection | UFAM- 0288 | Non-native (Manaus) |
| **SYNBRANCHIFORMES** |  |  |  |  |  |  |  |  |  |  |  |
| **Synbranchidae** |  |  |  |  |  |  |  |  |  |  |  |
| *Synbranchus* cf. *marmoratus* Bloch, 1795 |  | B |  | D | E |  |  |  | Goulding et al. 1988 | INPA- 15866; 19654 | South America |
| *Synbranchus madeirae* Rosen & Rumney, 1972 |  |  |  | D |  |  |  | H | INPA Fish Collection | INPA- 36396; 38821 | Neg/mid Amaz |
| *Synbranchus* sp. 'reticulado' |  |  |  |  | E |  |  |  | Zuanon et al. 2015 | INPA- 11835; 11839 | Neg |
| **CARANGIARIA** |  |  |  |  |  |  |  |  |  |  |  |
| **PLEURONECTIFORMES** |  |  |  |  |  |  |  |  |  |  |  |
| **Achiridae** |  |  |  |  |  |  |  |  |  |  |  |
| *Achiropsis nattereri* Steindachner, 1876 |  |  |  |  |  |  |  | H | Reis et al. 2003 | NMW- 14001 | Amaz/Neg |
| *Apionichthys menezesi* Ramos, 2003 |  |  |  |  |  |  |  | H | Ramos 2003 | MZUSP- 31052; 33385 | Neg/Ori/upp Amaz |
| *Apionichthys seripierriae* Ramos, 2003 | A |  |  |  |  | F |  |  | Ferreira et al. 2007 | MZUSP- 33389 | Amaz/Neg |
| *Hypoclinemus mentalis* (Günther, 1862) | A |  |  |  |  | F |  |  | Ferreira et al. 2007 | INPA- 11022; 16744 | Amaz/Ori/Gui |
| *Soleonasus finis* Eigenmann, 1912 |  |  |  |  |  |  |  | H | INPA Fish Collection | INPA- 36342 | Neg/Gui/upp Amaz |
| **OVALENTARIA** |  |  |  |  |  |  |  |  |  |  |  |
| **ATHERINOMORPHAE** |  |  |  |  |  |  |  |  |  |  |  |
| **BELONIFORMES** |  |  |  |  |  |  |  |  |  |  |  |
| **Belonidae** |  |  |  |  |  |  |  |  |  |  |  |
| *Belonion apodion* Collette, 1966 |  | B |  | D | E |  |  |  | Rapp Py-Daniel et al. 2017 | INPA- 14539; 14999 | Neg/mid Amaz |
| *Belonion dibranchodon* Collette, 1966 |  |  |  |  |  |  |  | H | Reis et al. 2003 | INPA- 11045 | Amaz/Ori |
| *Pseudotylosurus angusticeps* (Günther, 1866) |  | B |  | D |  |  |  | H | INPA Fish Collection | INPA- 36870 | Amaz/Par |
| *Pseudotylosurus microps* (Günther, 1866) |  | B |  | D |  |  |  | H | INPA Fish Collection | INPA- 16761; 16771 | Amaz/Ori/Gui |
| *Potamorrhaphis guianensis* (Jardine, 1843) |  | B |  | D |  |  |  |  | Lima et al. 2005 | INPA- 11725; 14538 | Amaz/Ori/Gui |
| *Potamorrhaphis petersi* Collette, 1974 |  | B |  | D |  |  |  |  | Goulding et al. 1988 | MZUSP- 59509; 61939 | Neg/Ori |
| **CYPRINODONTIFORMES** |  |  |  |  |  |  |  |  |  |  |  |
| **Poeciliidae** |  |  |  |  |  |  |  |  |  |  |  |
| *Fluviphylax gouldingi* Bragança, 2018 |  |  |  |  |  |  |  | H | Bragança 2018 | UFRJ- 9275 | Neg |
| *Fluviphylax obscurus* Costa, 1996 |  | B |  | D |  |  |  |  | Rapp Py-Daniel et al. 2017 | INPA- 30077; 38932 | Neg/Ori |
| *Fluviphylax pygmaeus* (Myers & Carvalho, 1955) |  | B |  | D | E |  |  |  | Goulding et al. 1988 | INPA- 15581; 22961 | Amaz/Ori |
| *Fluviphylax simplex* Costa, 1996 |  |  |  |  |  |  |  |  | INPA Fish Collection | INPA- 36421; 39409 | Amaz/Neg |
| *Fluviphylax wallacei* Bragança, 2018 |  |  |  |  |  |  |  |  | Bragança 2018 | UFRJ- 9080 | Neg |
| *Fluviphylax zonatus* Costa, 1996 |  |  |  |  |  |  |  | H | Reis et al. 2003 | INPA- 26620 | Neg |
| *Pamphorichthys* sp. |  |  |  |  |  |  |  | H | INPA Fish Collection | INPA- 47970 |  |
| *Poecilia reticulata* Peters, 1859 |  |  |  |  | E |  |  |  | INPA Fish Collection | INPA- 28241; 28330 | Non-native (Manaus) |
| *Xiphophorus hellerii* Heckel, 1848 |  |  |  |  | E |  |  |  | UFAM Fish Collection | UFAM- 0289 | Non-native (Manaus) |
| *Xiphophorus maculatus* (Günther, 1866) |  |  |  |  | E |  |  |  | UFAM Fish Collection | UFAM- 0287 | Non-native (Manaus) |
| **Rivulidae** |  |  |  |  |  |  |  |  |  |  |  |
| *Anablepsoides henschelae* Costa; Bragança& Amorim, 2013 |  |  |  |  |  |  |  | H | Costa et. al. 2013 | UFRJ- 9608 | Neg |
| *Anablepsoides micropus* (Steindachner, 1863) |  |  |  |  | E |  |  |  | Kemenes and Forsberg 2014 | INPA- 13224; 27776 | Neg/mid Amaz |
| *Anablepsoides ornatus* (Garman, 1895) |  |  |  | D | E |  |  |  | INPA Fish Collection | INPA- 15707; 26263 | Amaz/Neg |
| *Anablepsoides ottonii* Costa; Bragança & Amorim, 2013 |  |  |  |  |  |  |  | H | Costa et. al. 2013 | UFRJ- 9612 | Neg |
| *Anablepsoides roraima* Costa, Bragança & Amorim, 2013 |  |  |  |  |  |  |  | H | Costa et. al. 2013 | UFRJ- 9571 | Neg (Bra) |
| *Anablepsoides taeniatus*(Fowler, 1945) |  |  |  |  |  |  |  | H | INPA Fish Collection | INPA- 53020 | Amaz/Neg |
| *Laimosemion amanapira* (Costa, 2004) |  |  |  |  |  |  |  | H | Costa 2004b | UFRJ- 5929 | Neg |
| *Laimosemion gili* Valdesalici & Nielson, 2017 |  |  |  |  |  |  |  | H | Valdesalici and Nielson 2017 | ZUEC- 14575 | Neg |
| *Laimosemion jauaperi* Costa & Bragança, 2013 |  |  |  |  |  |  |  | H | Costa and Bragança 2013 | UFRJ- 9277 | Neg |
| *Laimosemion kirovskyi* (Costa, 2004) |  |  |  |  | E |  |  | H | Hrbek et al. 2004 | INPA- 21748; 27717 | Neg |
| *Laimosemion romeri* (Costa, 2003) |  |  |  |  |  |  |  | H | Costa 2003a | UFRJ- 5447 | Neg |
| *Laimosemion staecki* (Schindler & Valdesalici, 2011) |  |  |  |  |  |  |  | H | Schindler and Valdesalici 2011 | MTD- F 32344; 32348 | Neg |
| *Laimosemion uakti* (Costa, 2004) |  |  |  |  |  |  |  | H | Costa 2004b | INPA- 48928 | Neg |
| *Moema portugali* Costa, 1989 |  |  |  | D |  |  |  |  | Ferreira et al. 2007 | INPA- 12946; 36397 | Neg (Bra) |
| *Moema staecki* (Seegers, 1987) |  |  |  |  |  |  |  | H | Reis et al. 2003 | ZFMK- 14597 | Neg/mid Amaz |
| *Moema nudifrontata* Costa, 2003 |  |  |  |  |  |  |  | H | Costa 2003b | MCP 30684; 30685 | Neg (Bra) |
| **CICHLIMORPHAE** |  |  |  |  |  |  |  |  |  |  |  |
| **CICHLIFORMES** |  |  |  |  |  |  |  |  |  |  |  |
| **Cichlidae** |  |  |  |  |  |  |  |  |  |  |  |
| *Acarichthys heckelii* (Müller & Troschel, 1849) | A | B |  | D | E | F |  |  | Zuanon et al. 2008 | INPA- 11120; 13155 | Amaz/Gui |
| *Acaronia nassa* (Heckel, 1840) |  | B |  | D | E |  |  |  | Rapp Py-Daniel et al. 2017 | INPA- 28332; 36398 | Amaz/Gui |
| *Acaronia vultuosa* Kullander, 1989 |  |  |  |  |  |  |  | H | Reis et al. 2003 | INPA- 36426 | Neg/Ori |
| *Aequidens diadema* (Heckel, 1840) |  |  |  |  |  |  |  | H | Reis et al. 2003 | MZUSP- 29791 | Amaz/Ori |
| *Aequidens pallidus* (Heckel, 1840) |  |  |  | D | E | F |  |  | Zuanon et al. 2015 | INPA- 15391 | Amaz/Neg |
| *Aequidens tetramerus* (Heckel, 1840) |  | B |  | D | E |  |  |  | Goulding et al. 1988 | INPA- 1405 | Amaz/Ori/Gui |
| *Aequidens superomaculatum* Hernández-Acevedo, Machado-Allison & Lasso, 2015 |  |  |  |  |  |  |  | H | Hernández-Acevedo et al. 2015 | MBUCV- 35740 | Neg/Ori |
| *Apistogramma agassizii* (Steindachner, 1875) |  |  |  | D | E |  |  |  | Zuanon et al. 2015 | INPA- 28244; 31377 | Amaz/Neg |
| *Apistogramma brevis* Kullander, 1980 |  |  |  |  |  |  |  | H | Reis et al. 2003 | INPA- 38866 | Neg |
| *Apistogramma diplotaenia* Kullander, 1987 |  |  |  |  |  |  |  | H | Reis et al. 2003 | INPA- 42855 | Neg |
| *Apistogramma elizabethae* Kullander, 1980 |  |  |  |  |  |  |  | H | INPA Fish Collection | INPA- 42789; 42871 | Neg |
| *Apistogramma gephyra* Kullander, 1980 |  |  |  |  | E |  |  | H | Kullander and Ferreira 2005 | INPA- 26272; 39396 | Neg |
| *Apistogramma gibbiceps* Meinken, 1969 |  |  |  | D | E |  |  |  | Rapp Py-Daniel et al. 2017 | INPA- 34568; 38923 | Neg |
| *Apistogramma hippolytae* Kullander, 1982 |  |  |  |  | E |  |  |  | Zuanon et al. 2015 | INPA- 11946; 37749 | Neg/mid Amaz |
| *Apistogramma meinkeni* Kullander, 1980 |  |  |  |  | E |  |  | H | Kullander and Ferreira 2005 | INPA- 30445; 42842 | Neg |
| *Apistogramma mendezi* Römer, 1994 |  |  |  |  | E |  |  | H | Reis et al. 2003 | INPA- 28279; 28293 | Neg |
| *Apistogramma paucisquamis* Kullander & Staeck, 1988 |  |  |  |  | E |  |  |  | Rapp Py-Daniel et al. 2017 | INPA- 38904; 38927 | Neg |
| *Apistogramma personata* Kullander, 1980 |  |  |  | D |  |  |  | H | Britzki et al. 2014 | IRSNB- 576 | Neg |
| *Apistogramma pertensis* (Haseman, 1911) |  | B |  | D | E |  |  | H | Kullander and Ferreira 2005 | INPA- 38909; | Neg/mid Amaz |
| *Apistogramma pulchra* Kullander, 1980 |  |  |  |  | E |  |  | H | Ferreira et al. 2007 | INPA- 36091; 37008 | Neg/mid Amaz |
| *Apistogramma regani* Kullander, 1980 |  |  |  | D | E |  |  |  | Goulding et al. 1988 | INPA- 27862; 29873 | Neg |
| *Apistogramma resticulosa* Kullander, 1980 |  |  |  |  | E |  |  | H | Dagosta and de Pinna 2019 | MZUSP- 29705 | Neg/mid Amaz |
| *Apistogramma rupununi* Fowler, 1914 |  |  |  |  | E |  |  |  | Ferreira et al. 2007 | INPA- 37071; 39343 | Neg/Gui |
| *Apistogramma* sp. 'miuá' |  |  |  |  |  |  |  | H | INPA Fish Collection | INPA- 49775; 49841 | Neg |
| *Apistogramma steindachneri* (Regan, 1908) |  | B |  |  | E |  |  |  | Zuanon et al. 2008 | INPA- 30520; 36912 | Neg/Ori/Gui |
| *Apistogramma uaupesi* Kullander, 1980 |  |  |  |  |  |  |  | H | Kullander and Ferreira 2005 | INPA- 42952 | Neg/Ori |
| *Apistogramma wapisana*Römer, Hahn & Conrad, 2006 |  |  |  |  |  |  |  | H | INPA Fish Collection | INPA-51935 | Neg (Bra) |
| *Astronotus crassipinnis* (Heckel, 1840) |  | B |  | D |  |  |  |  | Ferreira et al. 2007 | INPA- 26459 | Amaz/Par |
| *Astronotus ocellatus* (Agassiz, 1831) |  | B |  | D |  |  |  |  | Zuanon et al. 1998 | INPA- 12063; 26472 | Amaz/Ori/Gui |
| *Biotodoma cupido* (Heckel, 1840) | A | B |  | D | E |  |  |  | Rapp Py-Daniel et al. 2017 | INPA- 30661; 36403 | Amaz/Gui |
| *Biotodoma wavrini* (Gosse, 1963) |  | B |  | D |  |  |  |  | Goulding et al. 1988 | INPA- 30658; 35553 | Amaz/Ori |
| *Biotoecus opercularis* (Steindachner, 1875) | A | B |  | D | E |  |  |  | Rapp Py-Daniel et al. 2017 | INPA- 36160; 36341 | Amaz/Neg |
| *Bujurquina mariae* Eigenmann, 1922 |  |  |  |  |  |  |  |  | Buckup et al. 2007 | NRM- 16646 | Neg/Ori |
| *Bujurquina* sp. 'manauara' |  |  |  |  |  |  |  | H | INPA Fish Collection | INPA- 13438 | Neg |
| *Caquetaia spectabilis* (Steindachner, 1875) |  | B |  | D |  |  | G |  | Ferreira et al. 2007 | INPA- 1397; 7491 | Amaz/Gui |
| *Chaetobranchopsis orbicularis* (Steindachner, 1875) |  | B |  |  |  |  |  | H | Reis et al. 2003 | INPA- 26465; | Amaz/Neg |
| *Chaetobranchus flavescens* Heckel, 1840 |  | B |  | D |  |  |  | H | Ferreira et al. 2007 | INPA- 6606; 39382 | Amaz/Ori/Gui |
| *Cichla monoculus* Spix & Agassiz, 1831 |  | B |  | D |  |  |  |  | Kullander and Ferreira 2006 | INPA- 25768; 35571 | Amaz/Gui |
| *Cichla nigromaculata* Jardine & Schomburgk, 1843 |  |  |  |  |  |  |  | H | Kullander and Ferreira 2006 | MZUSP- 92401; 6743 | Neg/Ori |
| *Cichla ocellaris* Bloch & Schneider, 1801 |  | B |  | D |  | F |  |  | Goulding et al. 1988 | INPA- 35980; 36349 | Amaz/Gui |
| *Cichla orinocensis* Humboldt, 1821 |  | B |  | D |  | F |  |  | Kullander and Ferreira 2006 | INPA- 35703; 43012 | Amaz/Ori |
| *Cichla temensis* Humboldt, 1821 |  | B |  | D |  | F |  |  | Kullander and Ferreira 2006 | INPA- 16126; 30713 | Amaz/Ori |
| *Cichlasoma amazonarum* Kullander, 1983 |  |  |  | D | E |  |  |  | INPA Fish Collection | INPA- 26470; 28236 | Amaz/Gui |
| *Cichlasoma bimaculatum* (Linnaeus, 1758 |  |  |  | D |  |  |  |  | Ferreira et al. 2007 | INPA- 7901; 39372 | Amaz/Ori/Gui |
| *Crenicara punctulata* (Günther, 1863) |  |  |  |  | E |  |  |  | Ferreira et al. 2007 | INPA- 39383 | Neg/upp Amaz |
| *Crenicichla adspersa* Heckel, 1840 |  |  |  |  |  |  |  |  | INPA Fish Collection | INPA- 1523; 1787 | Amaz/Neg |
| *Crenicichla alta* Eigenmann, 1912 |  | B |  | D | E |  |  |  | Zuanon et al. 2015 | INPA- 1398; 21935 | Amaz/Ori/Gui |
| *Crenicichla anthurus* Cope, 1872 |  |  |  | D |  |  |  |  | Ferreira et al. 2007 | MZUSP- 32768; 32769 | Amaz/Ori |
| *Crenicichla cincta* Regan, 1905 |  | B |  |  |  |  |  |  | Saint-Paul et al. 2000 | INPA- 2960 | Amaz/Neg |
| *Crenicichla inpa* Ploeg, 1991 |  |  |  |  | E |  |  |  | Zuanon et al. 2015 | INPA- 29366; 29435 | Amaz/Neg |
| *Crenicichla johanna* Heckel, 1840 | A | B |  | D | E |  |  |  | Kullander and Varella 2015 | INPA- 9937; 15725 | Amaz/Ori/Gui |
| *Crenicichla lenticulata* Heckel, 1840 |  | B |  | D | E |  |  |  | Kullander and Varella 2015 | INPA- 2946; 26408 | Amaz/Ori |
| *Crenicichla lugubris* Heckel, 1840 |  | B |  | D | E |  |  |  | Zuanon et al. 2015 | INPA- 1380; 7873 | Amaz/Ori/Gui |
| *Crenicichla macrophthalma* Heckel, 1840 |  | B |  | D |  | F |  |  | Zuanon et al. 1998 | INPA- 35577; | Amaz/Neg |
| *Crenicichla marmorata* Pellegrin, 1904 |  |  |  | D | E |  |  |  | INPA Fish Collection | INPA- 15868; 16023 | Amaz/Neg |
| *Crenicichla monicae* Kullander & Varella, 2015 |  |  |  |  |  |  |  | H | Kullander and Varella 2015 | MZUSP- 115494 | Neg |
| *Crenicichla notophthalmus* Regan, 1913 | A | B |  | D | E |  |  |  | Rapp Py-Daniel et al. 2017 | INPA- 1445; 1519 | Neg |
| *Crenicichla proteus* Cope, 1872 |  |  |  |  |  |  |  |  | INPA Fish Collection | INPA- 2915 | Amaz/Neg |
| *Crenicichla regani* Ploeg, 1989 |  | B |  | D |  |  |  |  | Rapp Py-Daniel et al. 2017 | INPA- 1521; 32808 | Amaz/Neg |
| *Crenicichla reticulata* (Heckel, 1840) |  | B |  | D |  |  |  |  | Goulding et al. 1988 | INPA- 2108; 22126 | Amaz/Gui |
| *Crenicichla semicincta*Steindachner, 1892 |  |  |  |  |  |  |  | H | INPA Fish Collection | INPA- 49623 | Amaz/Neg |
| *Crenicichla strigata* Günther, 1862 |  |  |  | D |  |  |  |  | Goulding et al. 1988 | MZUSP- 32869.0 | Amaz/Gui |
| *Crenicichla virgatula* Ploeg, 1991 |  |  |  |  |  |  |  |  | Ferreira et al. 2007 | INPA- 39405; 38778 | Neg (Bra) |
| *Crenicichla* *wallacii* Regan, 1905 |  |  |  | D |  | F |  |  | Lima et al. 2005 | MZUSP- 41281.0 | Neg/Gui |
| *Dicrossus filamentosus* (Ladiges, 1958) | A |  |  |  | E |  |  | H | Reis et al. 2003 | INPA- 37842 | Neg/Ori |
| *Dicrossus foirni* Römer, Hahn & Vergara, 2010 |  |  |  |  | E |  |  | H | Römer et al. 2010 | MZUSP- 106589 | Neg |
| *Dicrossus maculatus* Steindachner, 1875 |  |  |  |  | E |  |  |  | Ferreira et al. 2007 | INPA- 36331 | Neg/low Amaz |
| *Geophagus* aff. *brachybranchus*Kullander & Nijssen, 1989 |  |  |  |  |  |  |  | H | INPA Fish Collection | INPA- 50024; 22141 | Neg/Gui |
| *Geophagus altifrons* Heckel, 1840 | A | B | C | D |  | F |  |  | Zuanon et al. 1998 | INPA- 12062; 14680 | Amaz/Neg |
| *Geophagus proximus* (Castelnau, 1855) |  | B |  | D |  | F |  |  | Saint-Paul et al. 2000 | INPA- 35570; 39005 | Amaz/Neg |
| *Geophagus* sp. ‘branco’ |  |  |  |  |  |  |  | H | INPA Fish Collection | INPA- 35676; 36060 |  |
| *Geophagus* *surinamensis* (Bloch, 1791) |  | B |  | D |  |  |  |  | Goulding et al. 1988 | INPA- 3168; 4936 | Amaz/Gui |
| *Geophagus winemilleri*López-Fernández & Taphorn, 2004 |  |  |  |  |  |  |  | H | INPA Fish Collection | INPA- 11905; 53001 | Neg/Ori |
| *Guianacara cuyunii* López-Fernández, Taphorn Baechle & Kullander, 2006 |  | B |  |  |  |  |  |  | INPA Fish Collection | INPA- 36974 | Neg/Gui |
| *Guianacara dacrya* Arbour & Lopés-Fernández, 2011 |  |  |  |  |  |  |  | H | Arbour and Lopés-Fernández 2011 | INPA- 53146 | Neg/Gui |
| *Guianacara sphenozona* Kullander& Nijssen, *2011* |  |  |  |  |  |  |  | H | Ferreira et al. 2007 | INPA- 01404 | Neg/Gui |
| *Heros efasciatus* Heckel, 1840 |  | B |  | D | E |  |  |  | Zuanon et al. 2015 | INPA- 15699; 26529 | Amaz/Neg |
| *Heros notatus* (Jardine, 1843) |  |  |  | D |  |  |  | H | Reis et al. 2003 | INPA- 7891 | Neg/Gui |
| *Heros severus* Heckel, 1840 |  | B |  | D |  | F |  |  | Zuanon et al. 2008 | INPA- 3172; 15726 | Amaz/Ori |
| *Hoplarchus psittacus* (Heckel, 1840) |  | B |  |  |  | F |  |  | Zuanon et al. 2008 | INPA- 35662; 43723 | Amaz/Ori |
| *Hypselecara coryphaenoides* (Heckel, 1840) |  | B |  | D | E | F |  |  | Zuanon et al. 2015 | INPA- 17128; 26528 | Amaz/Ori |
| *Hypselecara temporalis* (Günther, 1862) |  | B |  | D |  | F |  |  | Ferreira et al. 2007 | INPA- 26450; 26466 | Amaz/Neg |
| *Ivanacara adoketa* (Kullander & Prada-Pedreros, 1993) |  |  |  |  |  |  |  | H | Reis et al. 2003 | MZUSP- 44685 | Neg |
| *Laetacara* cf. *curviceps* (Ahl, 1923) |  |  |  | D |  |  |  |  | Ferreira et al. 2007 | INPA- 36428 | Neg/low Amaz |
| *Laetacara flavilabris* (Cope, 1870) |  |  |  | D |  |  |  |  | Lima et al. 2005 | MZUSP- 6798 | Amaz/Neg |
| *Laetacara fulvipinnis* Staeck & Schindler, 2007 |  |  |  |  |  |  |  | H | Staeck and Schindler 2007 | MTD- F 30607 | Neg/Ori |
| *Laetacara thayeri* (Steindachner, 1875) |  |  |  | D | E |  |  | H | Ottoni 2015 | INPA- 28314 | Amaz/Neg |
| *Mesonauta festivus* (Heckel, 1840) |  | B |  | D |  |  |  | H | INPA Fish Collection | INPA- 26449; 26453 | Amaz/Par |
| *Mesonauta insignis* (Heckel, 1840) |  | B |  | D | E | F |  |  | Lima et al. 2005 | INPA- 1396; 36436 | Amaz/Ori |
| *Mesonauta guyanae* Schindler, 1998 |  |  |  |  |  |  |  | H | Reis et al. 2003 | ZMB- 32333 | Amaz/Gui |
| *Oreochromis niloticus* (Linnaeus, 1758) |  |  |  |  | E |  |  |  | INPA Fish Collection | INPA- 28276; 28329 | Non-native (Manaus) |
| *Pterophyllum altum* Pellegrin, 1903 |  |  |  |  |  |  |  | H | Reis et al. 2003 | MNHN- 1887 | Neg/Ori |
| *Pterophyllum leopoldi* (Gosse, 1963) |  | B |  | D |  |  |  |  | Ferreira et al. 2007 | MZUSP- 9590 | Amaz/Gui |
| *Pterophyllum scalare* (Schultze, 1823) |  | B |  | D |  |  |  | H | INPA Fish Collection | INPA- 13434; 22231 | Amaz/Gui |
| *Satanoperca acuticeps* (Heckel, 1840) |  | B |  | D |  |  |  |  | Goulding et al. 1988 | INPA- 7629; 30480 | Amaz/Neg |
| *Satanoperca daemon* (Heckel, 1840) |  | B |  | D |  |  |  |  | Goulding et al. 1988 | INPA- 42903 | Amaz/Ori |
| *Satanoperca leucosticta* (Müller & Troschel, 1849) |  |  |  | D |  |  |  |  | Ferreira et al. 2007 | INPA- 1394; 39088 | Amaz/Gui |
| *Satanoperca lilith* Kullander & Ferreira, 1988 |  | B |  |  | E |  |  |  | Zuanon et al. 2015 | INPA- 1541; 10489 | Amaz/Neg |
| *Satanoperca jurupari* (Heckel, 1840) |  | B |  | D | E | F |  |  | Lima et al. 2005 | INPA- 28298; 28343 | Amaz/Gui |
| *Symphysodon discus* Heckel, 1840 |  |  |  |  |  |  |  | H | Reis et al. 2003 | INPA- 25498 | Neg/Tro |
| *Taeniacara candidi* Myers, 1935 |  | B |  |  | E |  |  |  | Goulding et al. 1988 | INPA- 28345; 37047 | Amaz/Neg |
| *Uaru amphiacanthoides* Heckel, 1840 |  | B |  | D |  | F | G |  | Zuanon et al. 1998 | INPA- 7575; 26530 | Amaz/Neg |
| **INCERTAE SEDIS OVALENTARIA** |  |  |  |  |  |  |  |  |  |  |  |
| **Polycentridae** |  |  |  |  |  |  |  |  |  |  |  |
| *Monocirrhus polyacanthus* Heckel, 1840 |  |  |  | D | E |  |  |  | Zuanon et al. 2015 | INPA- 27759; 29416 | Amaz/Ori |
| *Polycentrus jundia* Coutinho & Wosiacki, 2014 |  |  |  |  |  |  |  | H | Coutinho and Wosiacki 2014 | MZUSP- 54586 | Neg |
| **EUPERCARIA** |  |  |  |  |  |  |  |  |  |  |  |
| **TETRAODONTIFORMES** |  |  |  |  |  |  |  |  |  |  |  |
| **Tetraodontidae** |  |  |  |  |  |  |  |  |  |  |  |
| *Colomesus asellus* (Müller & Troschel, 1849) | A |  |  | D |  |  |  |  | Ferreira et al. 2007 | INPA- 12346; 16753 | Amaz/Ori/Gui |
| **INCERTAE SEDIS EUPERCARIA** |  |  |  |  |  |  |  |  |  |  |  |
| **Sciaenidae** |  |  |  |  |  |  |  |  |  |  |  |
| *Pachypops fourcroi* (Lacepède, 1802) | A |  |  |  |  | F |  |  | Thomé-Souza and Chao 2004 | INPA- 16256; | Amaz/Ori/Gui |
| *Pachypops pigmaeus* Casatti, 2002 |  |  |  | D |  |  |  |  | Reis et al. 2003 | MZUSP- 34105 | Amaz/Neg |
| *Pachypops trifilis* (Müller & Troschel, 1849) | A | B | C |  |  |  |  |  | Saint-Paul et al. 2000 | INPA- 8503; 12364 | Amaz/Gui |
| *Pachyurus calhamazon* Casatti, 2001 | A |  |  | D |  |  |  | H | Reis et al. 2003 | MZUSP- 66684 | Neg (Bra) |
| *Pachyurus gabrielensis* Casatti, 2001 |  |  |  |  |  |  |  | H | Reis et al. 2003 | INPA- 36057 | Amaz/Ori |
| *Pachyurus junki* Soares & Casatti, 2000 | A |  |  |  |  |  |  |  | Ferreira et al. 2007 | INPA- 35749; 36043 | Amaz/Neg |
| *Pachyurus paucirastrus* Aguilera, 1983 | A |  |  |  |  |  |  | H | INPA Fish Collection | INPA- 36073; 36180 | Neg/Toc |
| *Pachyurus schomburgki* Günther, 1860 | A |  |  |  |  | F |  |  | Goulding et al. 1988 | INPA- 614; 35755 | Amaz/Ori |
| *Petilipinnis grunniens* (Jardine, 1843) | A | B |  |  |  |  |  |  | Reis et al. 2003 | INPA- 23031 | Neg/Ori/Gui |
| *Plagioscion auratus* (Castelnau, 1855) | A |  |  | D |  |  |  |  | Zuanon et al. 2008 | INPA- 35943 | Amaz/Ori/Gui |
| *Plagioscion squamosissimus* (Heckel, 1840) | A | B | C | D |  | F |  |  | Zuanon et al. 2008 | INPA- 10262; 12937 | Amaz/Ori/Gui/Par |
| *Plagioscion montei* Soares & Casatti, 2000 | A | B |  | D |  |  |  |  | Goulding et al. 1988 | INPA- 25791; 36805 | Amaz/Neg |
| **DIPNOI** |  |  |  |  |  |  |  |  |  |  |  |
| **LEPIDOSIRENIFORMES** |  |  |  |  |  |  |  |  |  |  |  |
| **Lepidosirenidae** |  |  |  |  |  |  |  |  |  |  |  |
| *Lepidosiren paradoxa* Fitzinger, 1837 |  | B |  |  |  |  |  |  | INPA Fish Collection | INPA- 25961 | Amaz/Ori/Gui/Par |
| **1165** |  |  |  |  |  |  |  |  |  |  |  |
